# Isoxazole‐Based Compounds Targeting the Taxane‐Binding Site of Tubulin

**DOI:** 10.1002/ardp.70031

**Published:** 2025-07-23

**Authors:** Miroslav Peřina, Márton A. Kiss, Jakub Bělíček, Veronika Vojáčková, Denisa Veselá, Renáta Minorics, István Zupko, Éva Frank, Radek Jorda

**Affiliations:** ^1^ Department of Experimental Biology, Faculty of Science Palacký University Olomouc Olomouc Czech Republic; ^2^ Department of Molecular and Analytical Chemistry University of Szeged Szeged Hungary; ^3^ Institute of Pharmacodynamics and Biopharmacy University of Szeged Szeged Hungary

**Keywords:** antiproliferative activity, cytoskeleton, isoxazoles, mitotic block, tubulin

## Abstract

Taxanes and other tubulin‐targeting medications are essential for treating advanced malignancies, especially in patients undergoing less aggressive chemotherapy. However, their clinical efficacy is often limited by significant off‐target toxicity and adverse side effects. In this study, the synthesis and characterisation of novel steroidal A‐ring‐fused isoxazoles, which were obtained through iodine‐mediated oxidative cyclization of dihydrotestosterone (DHT)‐derived α,β‐unsaturated oximes, are reported. According to mechanistic studies, the most potent compounds induced mitotic arrest and disrupted cytoskeletal integrity at low micromolar concentrations. The lead compound, **2j**, notably increased the rate of tubulin polymerisation in vitro and stabilised polymerised tubulin in the cells, leading to a G2/M block of the cell cycle. Molecular docking studies indicated that **2j** is bound preferably to the taxane site on tubulin, forming conserved interactions. MicroScale Thermophoresis was used to further study this binding and showed a nanomolar *K*_D_ for **2j**. The fact that **2j** maintained its activity in docetaxel‐resistant prostate cancer cells, demonstrating its ability to circumvent resistance pathways linked to existing therapies with taxane‐like drugs, supports its clinical relevance. Therefore, our results encourage additional research and development for its potential therapeutic use in cancer treatment, particularly in resistant cases.

## Graphical abstract

Lead compound **2j** targets the taxane site of tubulin with a nanomolar *K*_D_ and induces G2/M block by mitotic arrest at low micromolar doses. Prolonged mitotic arrest led to the induction of apoptosis in cancer cells, also in docetaxel‐resistant prostate cancer cells, suggesting its potential in taxane‐resistant cancer therapy.

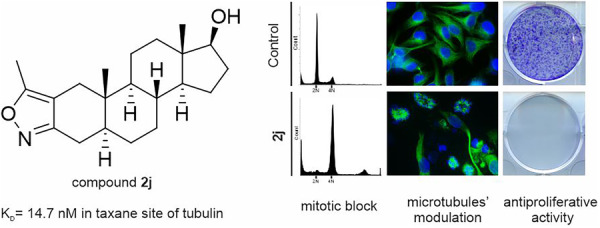

## 1 Introduction

Microtubules are dynamic polymers composed of tubulin protein subunits, essential for various cellular processes, such as maintaining the cellular structure, cell motility, shape and organisation of organelles, mitosis and meiosis as well as for intracellular transport and signalling [1]. Such importance in fundamental biological processes makes them an ideal target for potential therapeutic interventions [2, 3]. The formation of tubulin polymers from heterodimers of α‐ and β‐tubulin is a highly coordinated process involving guanosine triphosphate (GTP) hydrolysis. The resulting microtubules are in a state of dynamic instability [4].

**Figure 1:**
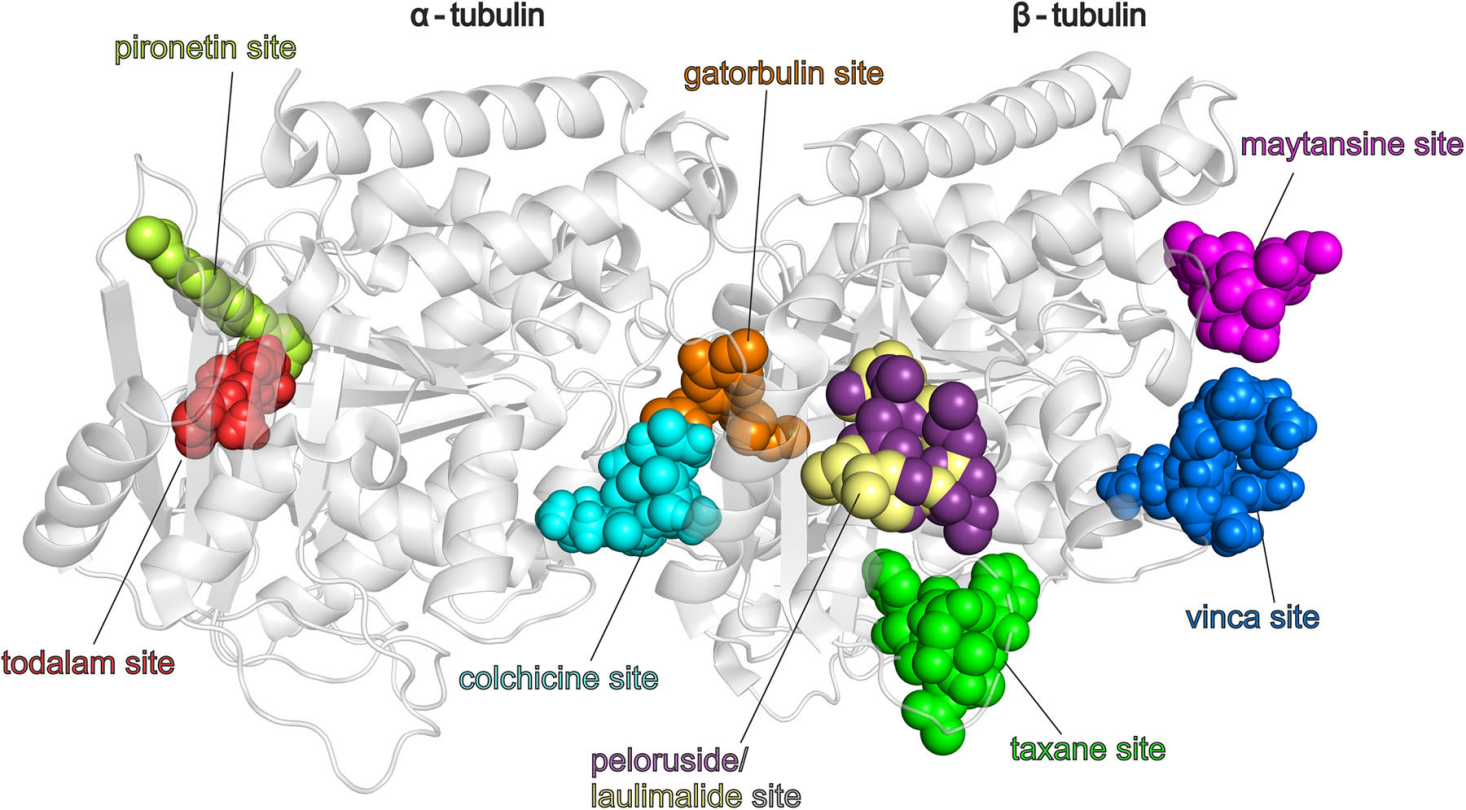
Binding sites identified on the α/β tubulin dimer (shown as grey cartoon) with their representative ligands shown as spheres with consistent coloured descriptions. The visualisation was prepared based on the structural alignment of the α/β tubulin dimer co‐crystallised with colchicine (cyan, PDB: 4O2B) with structures containing laulimalide (wheat, PDB: 4O4H), peloruside (purple, PDB: 4O4J), maytansine (magenta, PDB: 4TV8), vinblastine (blue, PDB: 5JT2), pironetin (lime green, PDB: 5LA6), todalam (red, PDB: 5SB3), paclitaxel (green, PDB: 6I2I) and gatorbulin (orange, PDB: 7ALR).

The GTP‐binding sites and other pockets form eight known targetable binding sites on the tubulin dimer (Figure 1). They were originally named upon the first ligands identified to bind there specifically [5]. These sites include the vinca, taxane, maytansine and peloruside/laulimalide sites on β‐tubulin [6], the colchicine and gatorbulin sites at the α‐ and β‐tubulin interface, and the pironetin and todalam sites on α‐tubulin [7] (Figure 1).

Based on their binding sites and related mechanisms of action, microtubule targeting agents can be categorised into stabilisers and destabilisers. Stabilisers, like paclitaxel, docetaxel and epothilone A, bind to the taxane and laulimalide/peloruside sites, enhancing microtubule stability [8]. Destabilisers, such as colchicine, nocodazole and vinca alkaloids, bind to the colchicine and vinca sites, preventing structural changes during polymerisation and inhibiting microtubule assembly [9, 10].

So far, several microtubule‐targeting agents have been approved by FDA, including stabilisers paclitaxel and docetaxel (for solid and haematological tumours), cabazitaxel (for metastatic prostate cancer in combination with prednisone), nab‐paclitaxel (for metastatic breast, lung, or pancreatic carcinomas), eribulin and ixabepilone (for metastatic breast cancer or liposarcoma). Approved microtubule destabilisers include vinorelbine (for metastatic lung cancer) and vincristine (for relapsed acute lymphoblastic leukaemia) [11]. Several others are under clinical investigation, for example, OXi4503 (combretastatin A1 prodrug) for paediatric acute myeloid leukaemia, lisavanbulin and plinabulin for glioblastoma [11], VERU‐111 (sabizabulin) for several tumour models including castration‐resistant prostate cancer [12, 13].

Despite the success of current tubulin‐targeting agents, there are still serious limitations hampering the effective treatment including off‐target toxicity and resistance development, which underline the need to improve or develop novel tubulin‐targeting compounds, as evidenced by previous preclinical publications [14, 15, 16].

The isoxazole pharmacophore with adjacent oxygen and nitrogen atoms in a five‐membered aromatic ring is a key molecular building block of many bioactive natural compounds and marketed drugs [17]. Its favourable pharmacological activity can be attributed to the 1,2‐position of the two electronegative heteroatoms capable of forming H‐bonding interactions with target proteins hardly accessible by other hetero‐ring systems. Furthermore, isoxazoles are valuable intermediates as masked 1,3‐dicarbonyl equivalents in the chemical syntheses of more complex molecules due to the relative ease of their cleavage under mild reductive conditions [18]. Among the various approaches available for the preparation of functionalised isoxazoles, the 1,3‐dipolar cycloaddition of alkenes and alkynes with nitrile oxides and the condensation reaction of hydroxylamine with 1,3‐dicarbonyl or α,β‐unsaturated carbonyl compounds are the most frequently used. Although the latter transformation usually requires harsh reaction conditions that limit synthetic options, some remarkable methods have recently been reported [19].

**Figure 2:**
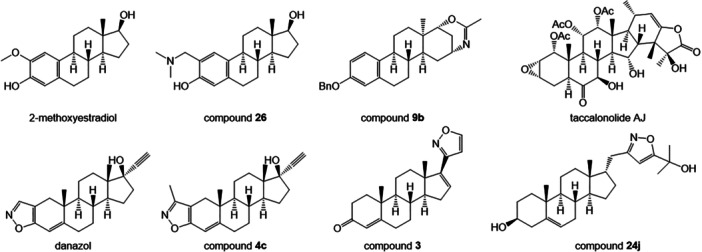
Steroidal compounds known as tubulin modulators (upper lane) and isoxazole‐containing steroidal compounds with diverse biological activity (lower lane).

Isoxazoles garnered attention in medicinal chemistry with many examples of FDA‐approved drugs as anti‐inflammatory, antimicrobial, antifungal, endocrine‐active and antiproliferative agents [20, 21]. A number of different steroidal isoxazole derivatives have been characterised to impact hormone signalling, mainly acting as aromatase inhibitors (compound **3** [22]), androgen receptor antagonists and CYP450 17A1 hydroxylase inhibitors (compound **24j** [23]) as well as apoptosis inductors (compound **4c** [24]) (Figure 2). The best known is danazol (Figure 2), a derivative of ethinyltestosterone [25, 26], which has been approved by the FDA as the hormonal treatment of endometriosis [27], but its impact on microtubules has not been described yet. The anticancer activity of several steroidal derivatives has been described as hormone receptor‐independent [28], but only a few of them act as tubulin modulators. These include tubulin destabilising clinical candidate 2‐methoxyestradiol [29, 30, 31] and its derivatives such as compound **26** [32, 33], which are known to bind to the colchicine site. On the other hand, the taxane site is targeted by stabilising 13α‐d‐homoestrones as compound **9b** [34] or taccalonolide [35] (Figure 2). The limited number of these steroidal modulators indicates an opportunity to explore novel compounds that can modulate tubulin dynamics.

Here, we describe the synthesis of new steroidal A‐ring‐fused isoxazoles by oxidatively cyclizing the DHT‐derived α,β‐unsaturated oximes using iodine. Nuclear magnetic resonance (NMR) spectroscopy of ^1^H and ^13^C and high‐resolution mass spectrometry (HRMS) were used to structurally characterise all of the novel derivatives. A panel of cancer cell lines was used to assess the antiproliferative efficacy of the synthesised compounds. Based on the observed mitotic block and cytoskeleton disruption induced by the most active compounds, novel derivatives were investigated for their direct effect on microtubules. Several compounds were confirmed as tubulin stabilisers, increasing tubulin polymerisation in vitro and in cancer cells.

## 2 Results and Discussion

### 2.1 Synthesis and Characterisation of DHT‐Based Isoxazole Derivatives

To obtain A‐ring‐fused isoxazole analogues structurally similar to the previously synthesised biologically active DHT‐derived pyrazoles [36], (hetero)arylidene (**1a**–**h**) [37], pyridin‐2‐ylidene (**1i**) and ethylidene derivatives (**1j**) [38] were reacted with hydroxylamine hydrochloride (1.5 equiv.) in the presence of sodium acetate in boiling ethanol to furnish the desired α,β‐unsaturated ketoxime intermediates. In all cases, complete conversion was detected within 2 h, with two distinct new spots on the thin layer chromatography (TLC) plate. The more intense one, which eluted first, probably corresponded to the (*Z*)‐isomer, and the other one—which was detectable only to a minor extent—was attributed to the (*E*)‐isomer [39]; however, after work‐up, the mixture was directly subjected to oxidative ring closure without separation. Next, elemental iodine (1.20 equiv.) and potassium carbonate as a base were used in dimethyl sulfoxide for the oxidative cyclisation [40]. The colour change of the reaction mixture from dark brown to yellow as well as TLC monitoring revealed complete conversion at 60°C within 1 h. Both oxime stereoisomers underwent cyclisation and the desired products (**2a**–**j**) were obtained in good yields after the work‐up procedure and chromatographic purification (Table 1).

**Table 1:** Synthesis of DHT‐derived A‐ring‐fused 5′‐substituted isoxazole derivatives.

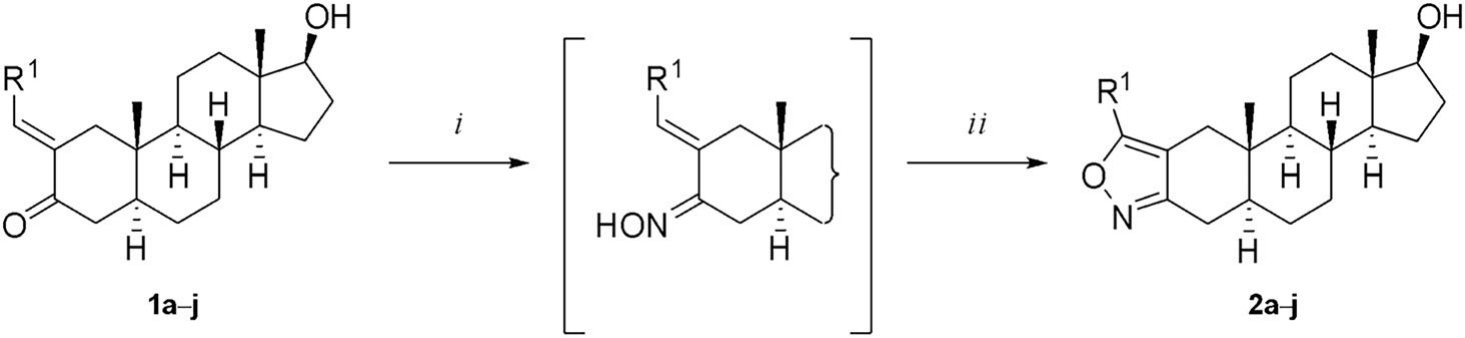				
Entry	Enone^a^	R^1^	Product^b^	Yield (%)^c^
1	**1a**	Ph	**2a**	62
2	**1b**	*p*‐CH_3_‐C_6_H_4_	**2b**	67
3	**1c**	*p*‐MeO‐C_6_H_4_	**2c**	64
4	**1d**	*p*‐F‐C_6_H_4_	**2d**	68
5	**1e**	*p*‐Cl‐C_6_H_4_	**2e**	71
6	**1f**	*p*‐Br‐C_6_H_4_	**2f**	61
7	**1g**	furan‐2‐yl	**2g**	54
8	**1h**	thiophen‐2‐yl	**2h**	67
9	**1i**	pyridin‐2‐yl	**2i**	55
10	**1j**	CH_3_	**2j**	55

*Note: Reagents and conditions*: (*i*) NH_2_OH · HCl, NaOAc, EtOH). (*ii*) I_2_, K_2_CO_3_, DMSO.

^a^ Compounds **1a**–**h**, **1j** were synthesised as described previously [36, 37, 38].

^b^ Heterocyclisation was performed with the crude ketoxime product.

^c^ Calculated for two steps from DHT after column chromatography.

The structures of the heterocyclic products (**2a**–**2j**) were confirmed by NMR spectroscopy (Supporting Information). In addition to the proton peaks characteristic of the sterane skeleton, the ^1^H NMR spectra also feature the 1‐H_2_ (two doublets) and 4‐H_2_ proton signals (two double doublets) of the A‐ring, which supports the formation of the condensed heteroring at the 2,3‐position. In the case of compounds **2a**–**i**, the signals of protons belonging to (hetero)aromatic rings with the appropriate substitution pattern in the 5′‐position of the isoxazole moiety appear in the aromatic region, while in the spectrum of **2j**, the singlet of 5′‐CH_3_ is at 2.27 ppm. On the ^13^C NMR spectra (APT), CH_3_ (C‐18 and C‐19) and CH carbon atoms (C‐5, C‐8, C‐9 and C‐14) can be observed as positive peaks in the aliphatic region, while quaternary (C‐10 and C‐13) and CH_2_ carbon atoms of the androstane core are shown as negative signals. The positive peak of C‐17 can be detected around *δ* = 82.0 ppm, and the signals of C‐2 and C‐3 as being part of the heteroaromatic ring are shifted downfield. For all steroidal isoxazoles, C‐5′ appears as a negative peak at the highest chemical shift.

### 2.2 DHT‐Based Isoxazoles Cause Changes in the Cell‐Cycle Phenotype

The routine analysis of the antiproliferative activity of the novel compounds over a panel of different cancer and noncancerous cell lines demonstrated a moderate effect on the viability of cancer cells (Supporting Information S2: Table 1). The precursor compound **1i** exhibited modest cytotoxicity across all tested cell lines. In contrast, the most active compounds **2g**, **2h** and **2j** displayed single‐digit micromolar GI_50_ values in HeLa, LAPC‐4, 22Rv1 and C4‐2 cancer cells while sparing the noncancerous BJ fibroblasts (Supporting Information S2: Table 2). Remarkably, treatment with the three mentioned compounds resulted in a pronounced alteration in the phenotype, particularly in HeLa cells. This was manifested as an increase in cell size and a round shape (Supporting Information S2: Figure 1), which is typically indicative of a G2/M phase arrest in the cell cycle. The LionheartFX (Biotek) live cell imaging was used to capture the effect in time‐lapse. In Supporting Information S3: Video 1, the vehicle‐treated cells display normal morphology and shape, actively proliferating throughout the 24 h imaging period. In contrast, the **2j**‐treated cells progressively lost their typical morphology (at 8 h of treatment), became rounder and larger, stopped proliferating and ultimately underwent apoptosis (upon 24 h) (Supporting Information S3: Video 2), supporting the G2/M phase arrest. Interestingly, 50 nM paclitaxel and 1 μM colchicine induced a similar cellular phenotype (Supporting Information S2: Figure 1) [41, 42].

**Figure 3:**
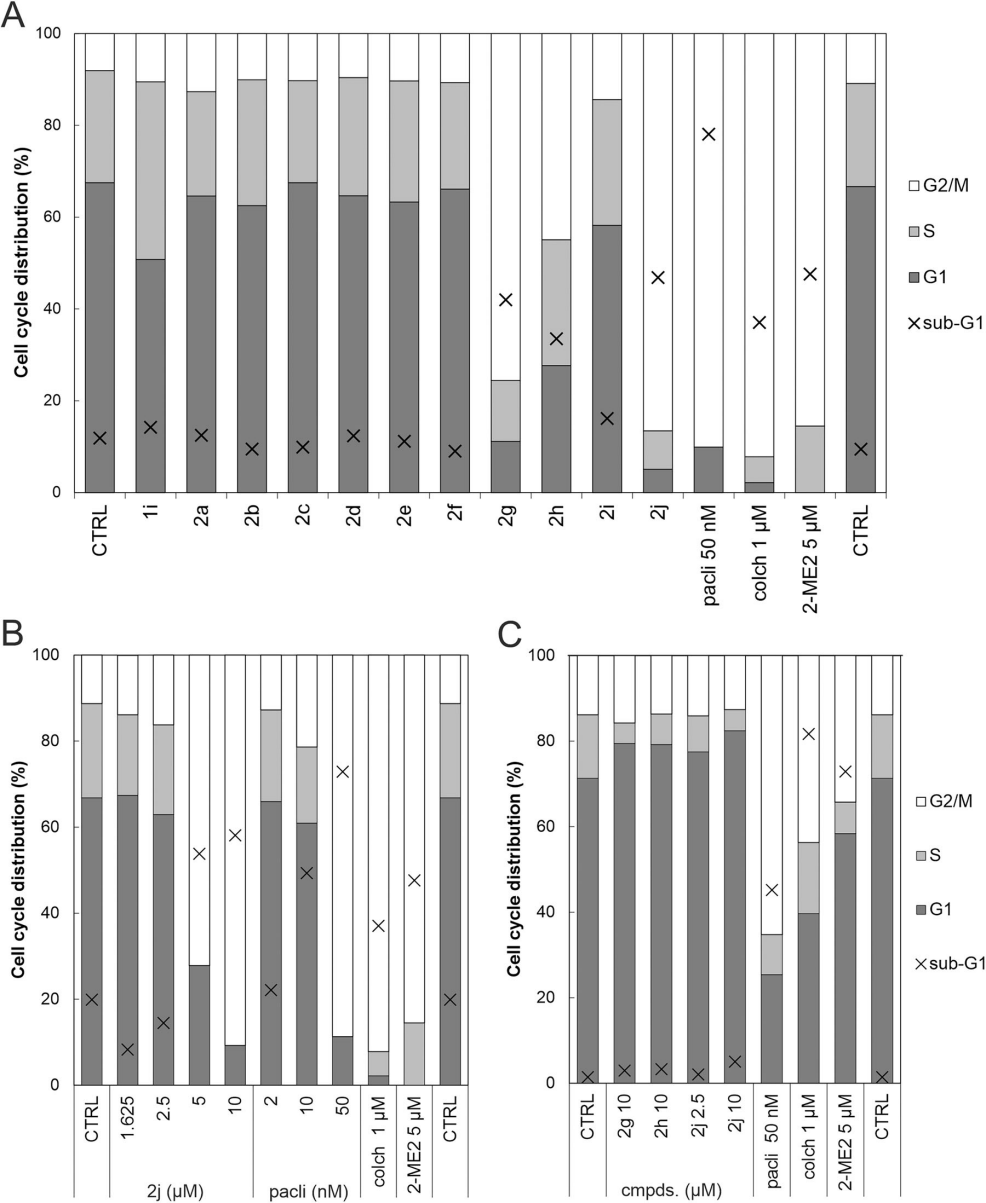
(A) Cell‐cycle analysis of HeLa cells upon 24 h treatment with the entire set of compounds (10 μM), compared with standards 50 nM paclitaxel (pacli), 1 μM colchicine (colch) and 5 μM 2‐methoxyestradiol (2‐ME2). (B) Dose‐dependent effect of **2j** and paclitaxel on HeLa cell‐cycle distribution upon 24 h treatment. (C) Cell‐cycle analysis of BJ treated for 24 h with **2g**, **2h**, **2j**, and standards in depicted concentrations. All plots show the percentage distribution of analysed cells across the phases of the cell cycle (G1, S, G2/M), including a sub‐G1 population representing apoptotic debris. DNA content was measured by propidium iodide staining using flow cytometry.

**Figure 4:**
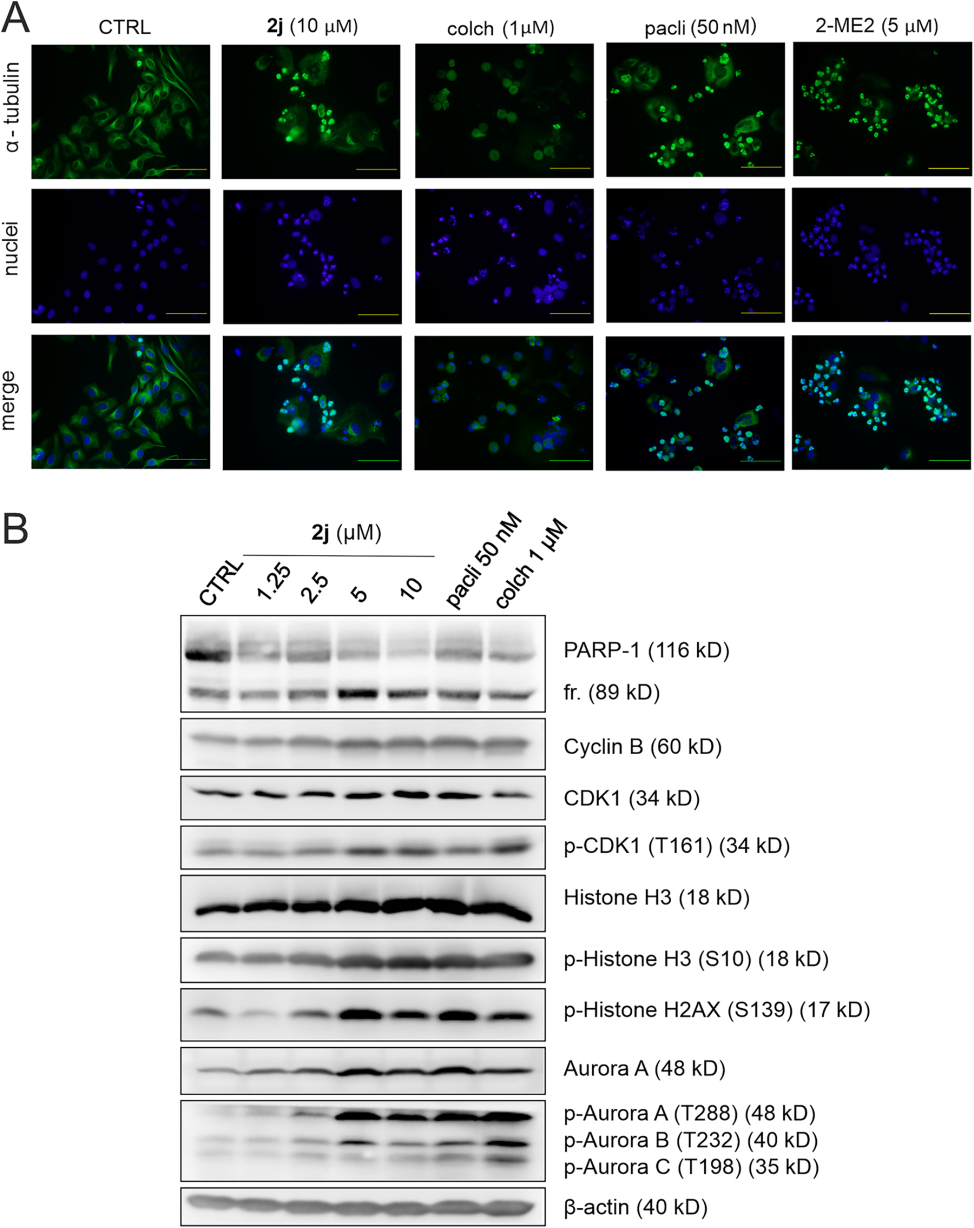
(A) Immunofluorescence images of HeLa cells upon 24 h treatment with 10 µM **2j**, 50 nM paclitaxel (pacli), 1 μM colchicine (colch), or 5 μM 2‐methoxyestradiol (2‐ME2). Alpha‐tubulin was stained by Alexa 488‐conjugated antibody and nuclear DNA by DAPI. The scale bar represents 100 μm, magnification of ×400. A merge of the two channels was performed in ImageJ software. (B) Immunoblotting analysis of HeLa cells upon the 24 h treatment with different concentrations of **2j** or 50 nM paclitaxel (pacli), 1 μM colchicine (colch). The β‐actin level served as a loading control.

The cell cytometry analysis clearly demonstrated that 10 µM concentrations of **2j** and **2g** markedly elevated the proportion of HeLa cells in the G2/M phase, exceeding 75% after 24 h treatment. Derivative **2h** also caused ~45% cell arrest in the G2/M phase of the cell cycle under the same experimental conditions (Figure 3). The only other compound that exhibited a similar effect, albeit at a higher concentration, was **2i**, which reached 60% in the G2/M at 20 µM. No other compound exhibited a comparable effect at concentrations up to 40 µM (Supporting Information S2: Figure 2). The results highlighted that the presence of a small hydrophobic substituent (methyl in the case of **2j**) or a smaller five‐membered heteroring (furan or thiophene) in the C5′ position on the isoxazole resulted in the most pronounced impact on the cell cycle of HeLa cells, in contrast to larger six‐membered (hetero)aromatic substituents. The results further indicated that compound **2j** was the most active derivative within the rest of the cancer cell lines (LAPC‐4, 22Rv1 and C4‐2), leading to at least 40% G2/M arrest in a concentration of 10 µM (Supporting Information S2: Figure 3). Subsequent analysis of the **2j**‐treated HeLa cells confirmed a dose‐dependent block in the G2/M phase accompanied by an increase in the level of sub‐G1 cells (cell debris) (Figure 3). Importantly, the same effect on the cell cycle was observed with low micromolar concentrations of paclitaxel, a known tubulin stabiliser [41], and colchicine with 2‐methoxyestradiol, both known tubulin destabilisers [31, 43, 44, 45] (Figure 3). We further investigated the effect of a structurally similar steroidal compound danazol (FDA‐approved to treat endometriosis), but it did not induce any significant changes in the cell‐cycle distribution up to 40 μM in the tested cell lines (Supporting Information S2: Figure 4).

Most importantly, none of the active compounds **2g**, **2h**, **2j** induced G2/M arrest in the noncancerous BJ fibroblasts, in contrast to 5 μM 2‐methoxyestradiol or 1 μM colchicine, which increased the percentage of G2/M cells to 35% and 45%, respectively. The increase of G2/M cells upon treatment with 50 nM paclitaxel was even more pronounced (Figure 3). Taken together, the novel derivatives induced an interesting cell‐cycle phenotype that had not been previously observed for structurally similar A‐ring fused pyrazoles, pyridines, or quinolines [36, 46].

**Figure 5:**
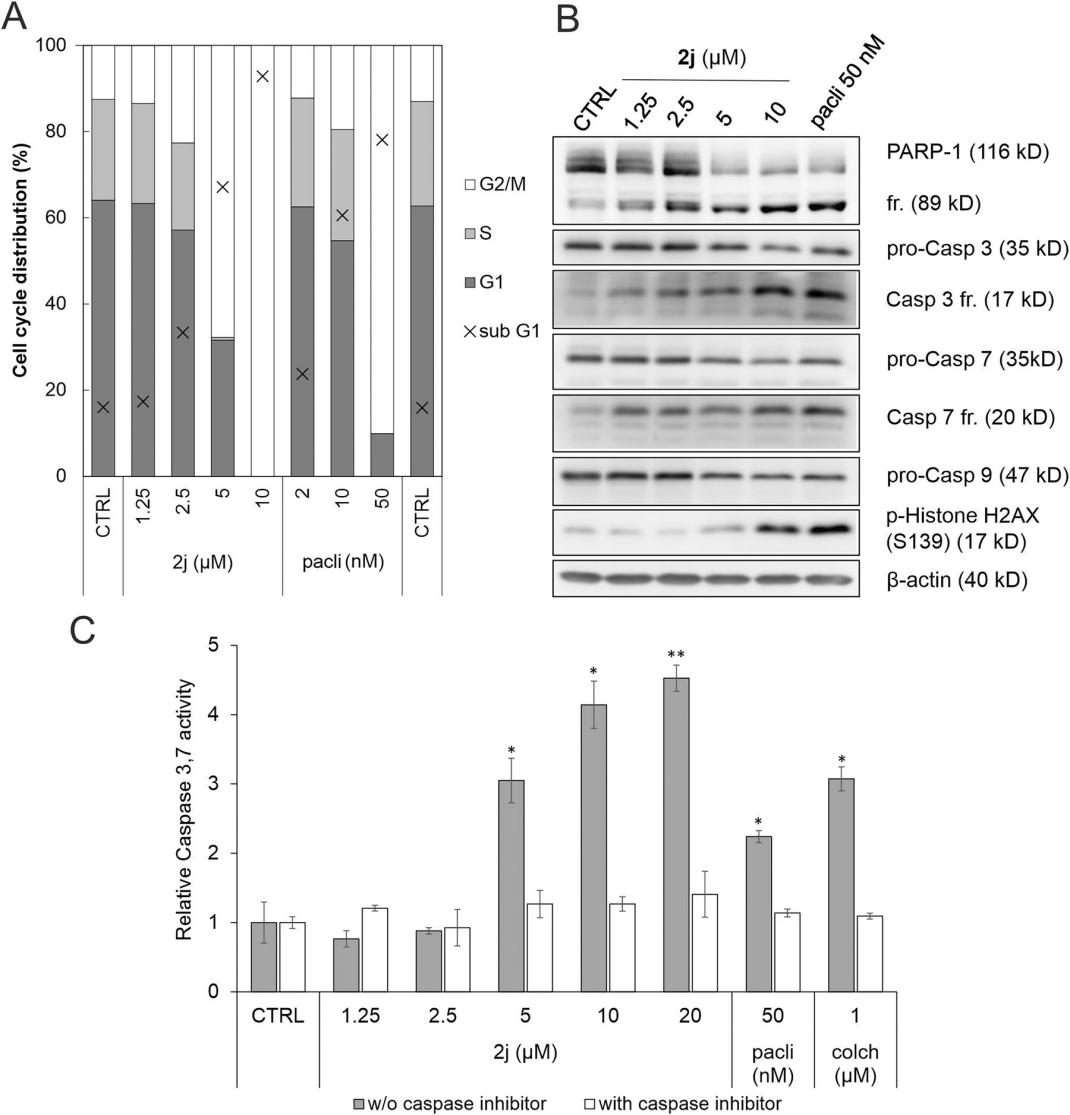
(A) Cell‐cycle analysis of HeLa cells upon 72 h treatment with **2j** or paclitaxel (pacli) in depicted concentrations. Cell‐cycle distribution was calculated from the DNA content measured by fluorescence of propidium iodide using flow cytometry. (B) Immunoblotting analysis of HeLa cells upon 72 h treatment with **2j** or paclitaxel (pacli) at indicated concentrations. The pro‐Casp and Casp fr. represent the pro‐Caspases and Caspase fragments levels, respectively. The β‐actin level served as a loading control. (C) Relative caspase 3/7 activity in HeLa cells treated with **2j**, paclitaxel (pacli), or colchicine (colch) for 24 h. Activities were measured as a fluorescence increase in the presence of the caspase substrate Ac‐DEVD‐AMC only (grey) or combined with the caspase inhibitor Ac‐DEVD‐CHO (white). Data were normalised to an untreated control and statistical analysis was carried out by means of one‐way ANOVA, where * indicates significance at *p* < 0.05 and ** at *p* < 0.01 compared to the untreated control samples. Results are from two independent experiments performed in duplicates.

To evaluate the kinetics of the mitotic blockage, we performed different exposures of HeLa cells to **2j** and paclitaxel, followed by washout of compounds and subsequent cultivation for another 24 h. No observable effect was seen after 1 h treatment by 10 μM **2j** or 50 nM paclitaxel, either after 24 h washout (Supporting Information S2: Figure 5). Upon 4 h treatment, both **2j** and paclitaxel induced an increase in G2/M percentage from 18% to 35%, which remained stable also after washout. Interestingly, subsequent cultivation of washed cells led to increased sub‐G1 percentage indicating induction of apoptosis and DNA fragmentation. Ultimately, 24 h treatment of both compounds induced a strong G2/M block as already observed previously. However, washout of **2j** and further 24 h cultivation seemed to reactivate the cell‐cycle progression compared with massive sub‐G1 percentage in the case of 50 nM paclitaxel. In addition, 48 h treatment showed stable G2/M blockage with high sub‐G1 percentage in both **2j** and paclitaxel in the studied concentration (Supporting Information S2: Figure 5). These findings suggest that **2j** induced a reversible G2/M block of the cell cycle.

### 2.3 Compound 2j Induced Changes on Microtubules, Leading to Mitotic Block Followed by Induction of Apoptosis

To further elucidate the mechanism of action of **2j**, we performed immunofluorescence staining of α‐tubulin in the microtubule network of treated HeLa cells. Several antimitotic agents have been used as controls, namely colchicine, 2‐methoxyestradiol and paclitaxel.

**Figure 6:**
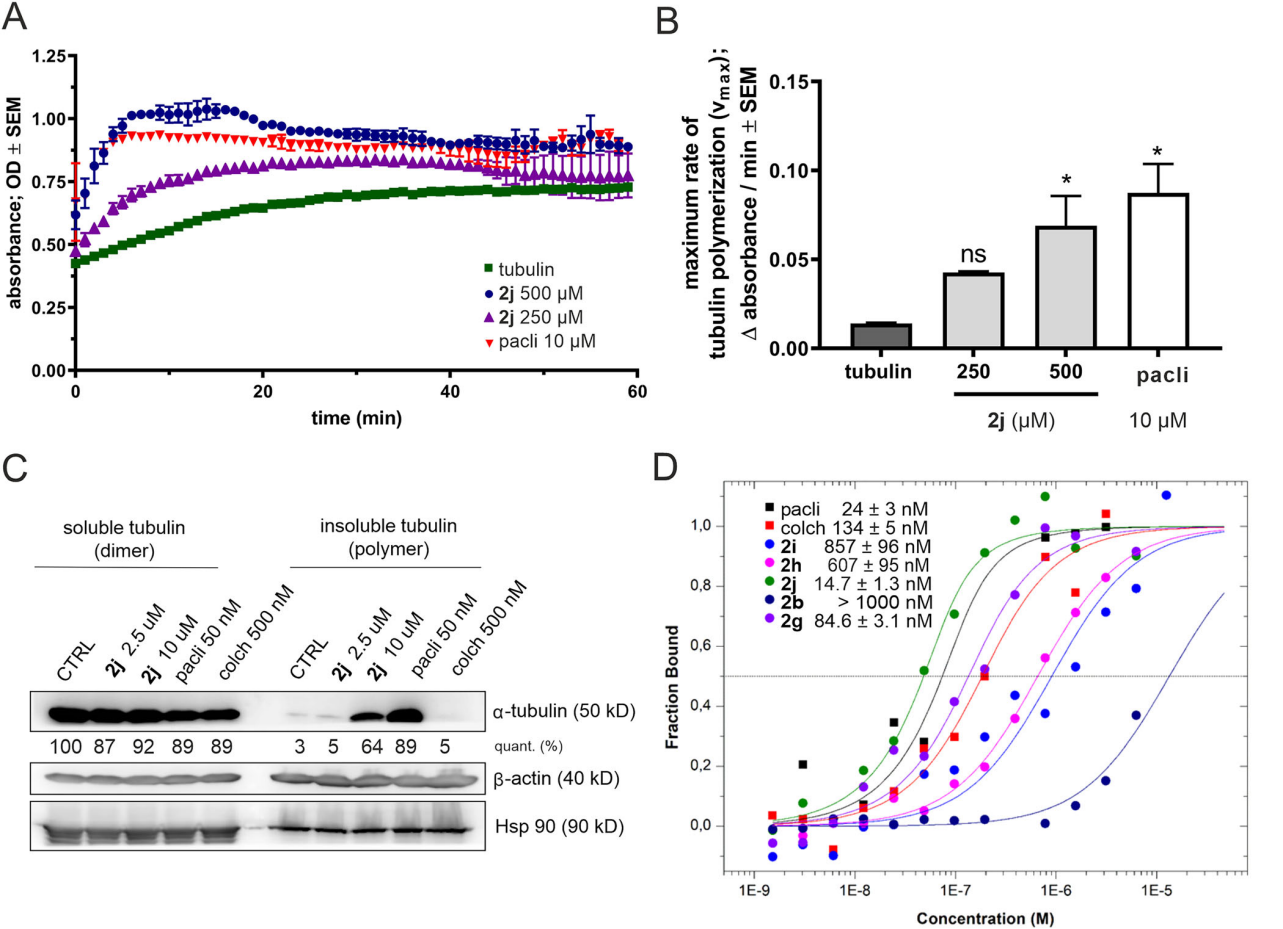
(A, B) Rate of porcine pure tubulin polymerisation in the presence of 250 μM or 500 μM **2j** or 10 μM paclitaxel (pacli) based on the absorbance measurement kit. Statistical analysis was carried out by means of one‐way ANOVA; *indicates significance at *p* < 0.05 compared with the negative control samples treated with vehicle (tubulin). Results are from two independent experiments performed in duplicates. (C) Western blot of α‐tubulin soluble (dimer) and insoluble (polymer) fractions isolated from HeLa cells treated by **2j**, 50 nM paclitaxel (pacli), or 500 nM colchicine (colch) for 12 h. Levels of β‐actin and Hsp 90 served as two independent loading controls. (D) Dissociation constants (*K*_D_) for selected compounds and standards binding into the porcine pure tubulin derived from their binding curves measured by MicroScale Thermophoresis (MST) (mean ± SD from two independent measurements).

HeLa cells showed gradual changes of the tubulin filaments with the final loss of cytoskeleton structure in the presence of 10 μM concentration of **2j** (Supporting Information S2: Figure 6), comparable with 2‐methoxyestradiol and paclitaxel, but opposite to the colchicine phenotype (Figure 4). Interestingly, colchicine and 2‐methoxyestradiol, both being microtubule destabilisers, induced different cellular phenotypes [33, 41, 42].

**Figure 7:**
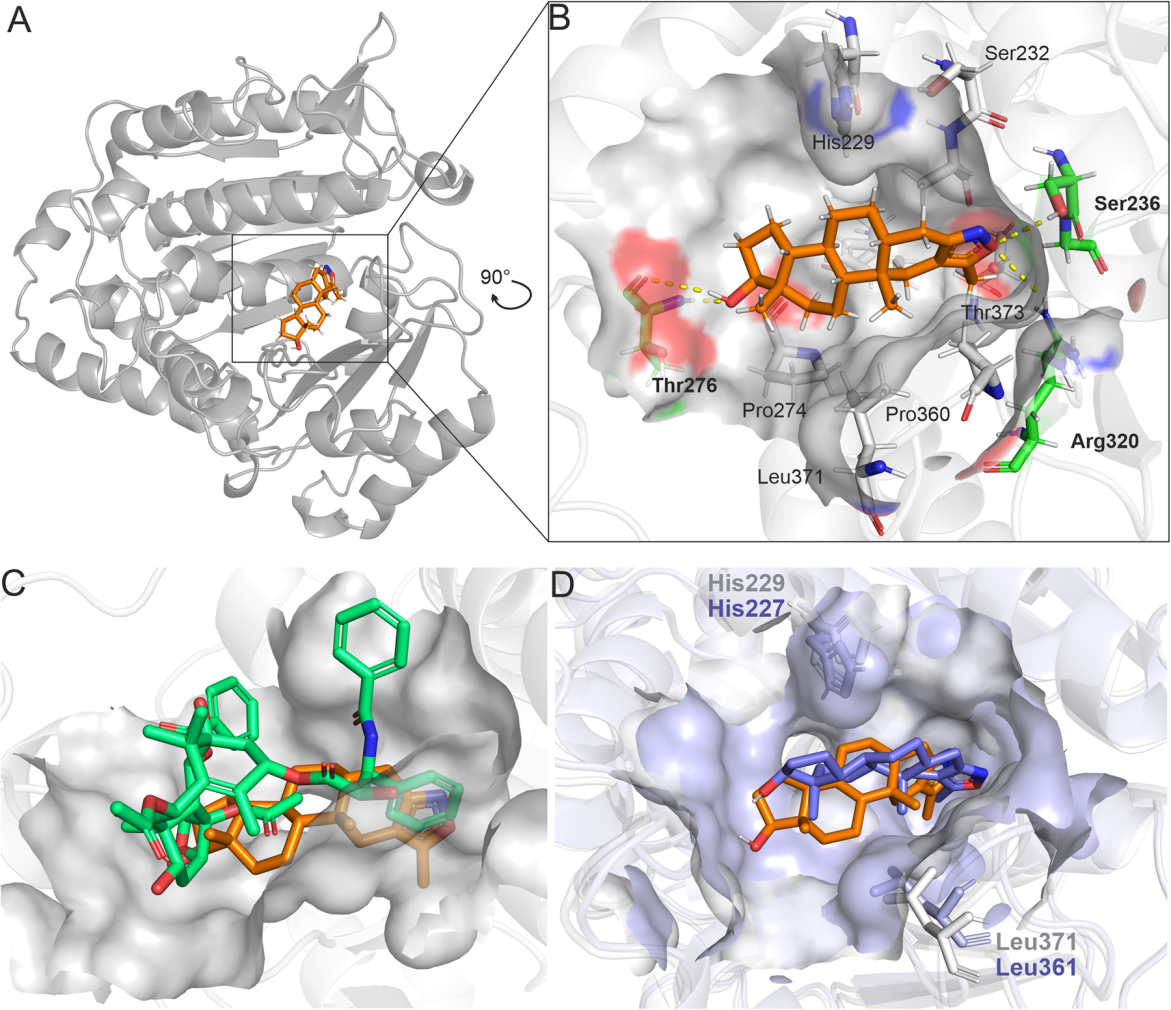
(**A**) Binding mode of **2j** (orange) in the β‐tubulin based on molecular docking into the taxane site of cryo‐EM structure of human HeLa tubulin (grey, PDB: 6I2I). (B) 3D‐interaction network of **2j** (orange) in the taxane site of PDB: 6I2I with the shown surface. Protein is shown in grey, carbons of residues forming the hydrophobic interactions are shown in grey and labelled regularly, residues forming hydrogen bonds (yellow dashed lines) are shown in green with bold labels. (C) Binding mode of **2j** in the taxane site aligned with the original ligand paclitaxel (green) position in PDB: 6I2I. (D) Alignment of the taxane site of human HeLa tubulin (grey, PDB: 6I2I) and porcine tubulin (blue, PDB: 7TQY) with most favoured poses of **2j** shown in orange and blue, respectively. Positions of respective His and Leu influencing the size and shape of the taxane site are shown. Heteroatoms are coloured as usual, N in blue and O in red.

We further analysed the effect of **2j** in C4‐2 and BJ cells. The results indicated that **2j** induced the stabilisation of tubulin filaments (in a paclitaxel‐like manner), leading to the loss of cytoskeleton dynamics and eventual condensation of the cytoskeleton around the nucleus during the mitotic block in the C4‐2 cell line (Supporting Information S2: Figure 7). Importantly, noncancerous BJ cells showed nearly no effect upon treatment with **2j**, but they were sensitive to colchicine and paclitaxel and exhibited their typical phenotype (Supporting Information S2: Figure 7), corresponding to previous results from cytometric analysis. Our observation clearly showed that **2j** targeted microtubules similarly to paclitaxel but led to the selective mitotic blockage of cancer cells.

To support our results, we further explored the changes in the expression of known mitotic markers in response to treatment by western blot analysis. Compound **2j** markedly induced the expression of cyclin B1 and increased the activation phosphorylation of CDK1 (T161) and histone H3 (S10), all known mitotic markers [47], confirming the cell‐cycle arrest in mitosis (Figure 4). Moreover, an observed dose‐dependent increase in the phosphorylation of Aurora proteins was consistent with the onset of mitotic block, as previously published [41]. At concentrations above 2.5 μM of **2j**, we observed the cleavage of PARP‐1 (known caspase substrate), producing its 89 kDa fragment and prominent phosphorylation of histone H2AX (S139), both of which occur during the initiation of apoptosis, allowing DNA fragmentation. The same patterns were also observed in the paclitaxel‐ and colchicine‐treated HeLa samples (Figure 4).

To elucidate the general ability of novel derivatives to induce apoptosis in cancer cells, we performed several analyses to assess this process in HeLa cells. At first, cellular response following a prolonged exposure of the candidate compound **2j** was elucidated, analysing cell cycle and protein markers by immunoblotting upon a 72‐h treatment. After the treatment with **2j** and paclitaxel, the cells remained in the mitotic block consistently with 24 h treatment (Figure 3), but the majority of them were undergoing apoptosis, as displayed by the percentage of sub‐G1 cells (reflecting DNA fragmentation) above 75% (Figure 5). Western blot analysis of HeLa cell lysates post 72‐h exposure to several **2j** concentrations and 50 nM paclitaxel revealed a dose‐dependent elevation in the 89 kDa PARP‐1 fragment level, a well‐established caspase substrate, confirming apoptosis induction. Additionally, we observed dose‐dependent cleavage of Caspases 3, 7 and 9, alongside an increase in Ser‐139 phosphorylation on histone H2AX, a marker essential for DNA fragmentation during apoptosis (Figure 5).

To further confirm caspase activation, we employed fluorimetric caspases 3 and 7 activity assay (with the substrate Ac‐DEVD‐AMC) in HeLa cells treated with **2j** for 24 h. We observed significant dose‐dependent caspase activation starting from 5 µM and reaching a 4.5‐fold increase over controls at 20 µM of **2j**. Similar effects were achieved by 50 nM paclitaxel and 1 µM colchicine treatments [43, 48]. Crucially, the caspase‐specific inhibitor Ac‐DEVD‐CHO completely inhibited this activity, underscoring the specificity of the caspase assay.

### 2.4 Candidate Compound 2j Binds Purified Tubulin and Affects Its Polymerisation In Vitro

The precise mechanism of action of our novel derivatives was elucidated by analysing tubulin polymerisation under different conditions. First, the absorbance‐based in vitro tubulin polymerisation assay [49, 50] was performed. The initial state of the tubulin is the dimer form, and in the presence of GTP, it undergoes polymerisation into filaments through nucleation, growth and steady‐state equilibrium, as described by the curve. The tubulin solution scatters light proportionally with the concentration of tubulin polymers. This assay clearly proved that our candidate compound **2j** increases the rate of tubulin polymerisation in a dose‐dependent manner, similar to paclitaxel (Figure 6).

Further, we followed the published protocol [51] for the isolation of the soluble (depolymerised monomers or dimers) and insoluble (polymer) tubulin fractions in treated cells, followed by their analysis by immunoblotting. Tubulin‐stabilising compounds (e.g., paclitaxel) should increase the amount of polymerised tubulin in contrast to tubulin‐destabilising agents (e.g., colchicine). Our results show that **2j** increased the fraction of polymerised tubulin in treated HeLa cells, suggesting the stabilisation of filaments in cells, as also shown in the case of paclitaxel.

**Figure 8:**
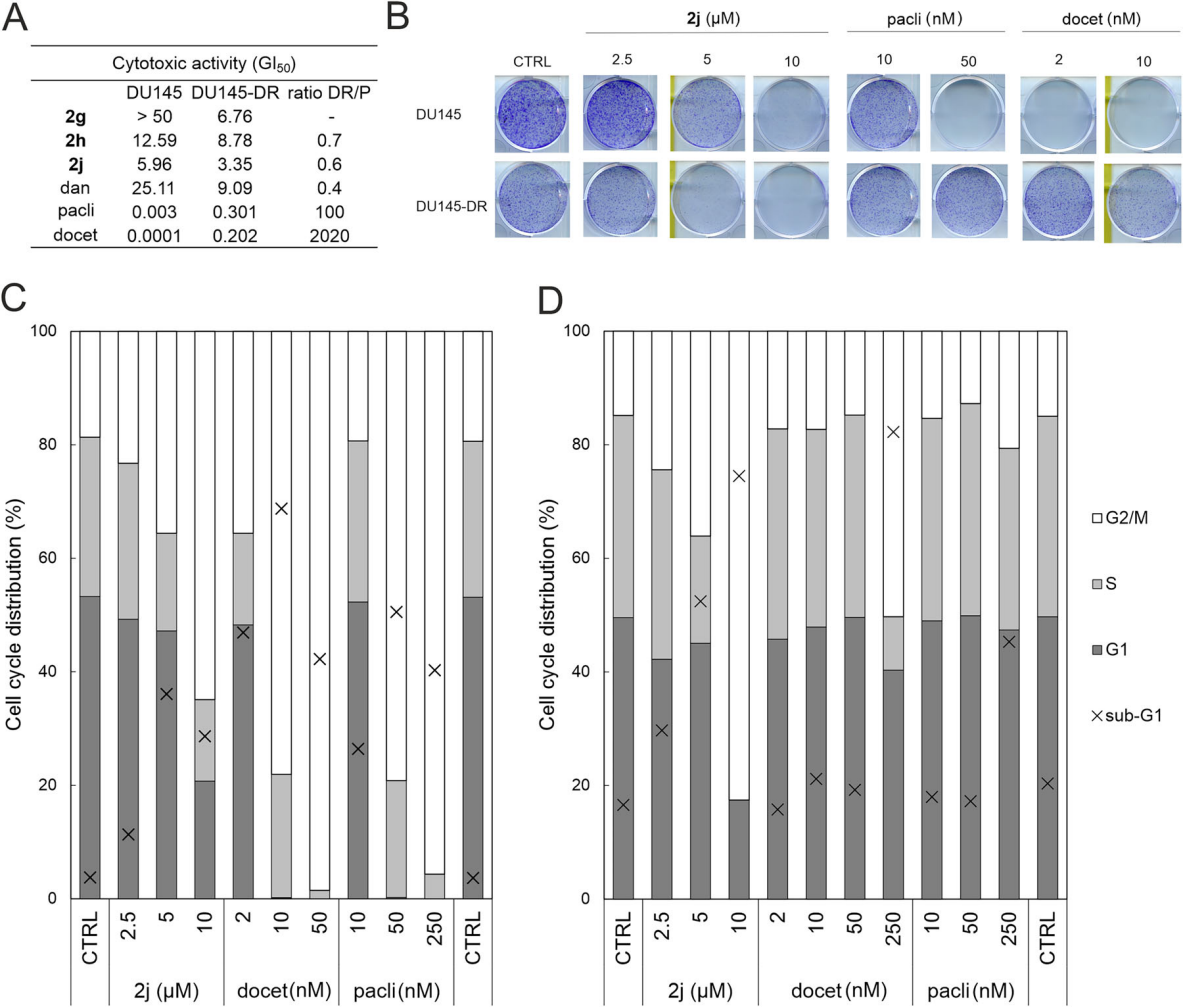
(A) Viability of DU145 and docetaxel‐resistant DU145‐DR upon treatment with selected compounds and standards danazol (dan), paclitaxel (pacli), or docetaxel (docet) for 72 h; mean of GI_50_ from two independent experiments is shown. Standard deviations are shown in Supporting Information S2: Table 2. The ratio DR/P (DU145‐DR:DU145 parental) was calculated from the GI_50_ values. (B) Colony formation assay of DU145 and docetaxel‐resistant DU145‐DR upon 10‐day treatment with candidate compound **2j**, paclitaxel (pacli), and docetaxel (docet) in depicted concentrations. (C) Cell‐cycle analysis of DU145 and (D) DU145‐DR cells upon 72 h treatment with **2j,** docetaxel (docet), or paclitaxel (pacli) in depicted concentrations. Plots show the percentage distribution of analysed cells across the phases of the cell cycle (G1, S, G2/M), including a sub‐G1 population representing apoptotic debris. DNA content was measured by propidium iodide staining using flow cytometry.

To complement our previous results, MicroScale Thermophoresis (MST) was conducted to determine the binding affinities of **2a**–**i** to purified porcine tubulin, isolated based on the previously published protocol [52] (see Supporting information S2: Figure 8 for verification of the purity and the integrity of tubulin). The most potent compounds from the previous assays, **2g** and **2j**, displayed a strong affinity with dissociation constants (*K*_D_) in the low nanomolar range, similar to those observed for paclitaxel and colchicine (Figure 6) with published nanomolar *K*_D_ [53, 54]. Compounds **2h** and **2i** exhibited lower affinity than **2g** or **2j** with *K*_D_ values in the hundreds of nanomolar, which also correlated with their observable activities in cells. However, compound **2b** was unable to saturate the protein in a concentration below 1 μM (Figure 6), similar to the remaining compounds (**2a**, **2c**–**f**). Furthermore, *K*_D_ could not be determined due to increased protein aggregation at high concentrations of these ligands.

### 2.5 Candidate Compound 2j Binds Into the Taxane Site of Tubulin

To prove and describe the interaction of our candidate compounds with tubulin, we first performed the molecular docking of **2j** and paclitaxel into the whole cryo‐EM structure of the tubulin dimer extracted from HeLa cells, stabilised by paclitaxel (PDB: 62I2). This structure displays a reasonable resolution and seems to be an ideal model since HeLa cells were used for the majority of experiments and the binding site occupied by paclitaxel displayed proper conformation for compounds acting in a taxane‐like mechanism [55]. It was shown that **2j** preferentially binds deep into the taxane binding site with a high binding score LF dG (–8.33 kcal/mol), with the C5′ methyl group buried deep in the hydrophobic pocket and the whole steroid core positioned at the base of the binding site (Figure 7). The isoxazole ring of **2j** reached the conserved residues Ser236 and Arg320 to interact via hydrogen bonds, while on the other end of the molecule, the C‐17 hydroxy group formed a hydrogen bond with Thr276. The sterane core was positioned by a wide network of hydrophobic interactions with His229, Ser232, Pro274, Pro360, Leu371 and Leu373 **(**Figure 7, Supporting Information S2: Figure 9). The binding score of the original ligand paclitaxel from docking (–10.30 kcal/mol) is stronger, but comparing the binding modes, the heteroaromatic isoxazole ring of **2j** aligned well with the phenyl ring C’ of the paclitaxel tail, while the d‐ring of **2j** with C17‐OH aligned with the substituted paclitaxel epoxy‐d‐ring and thus resembled a bridge that accessed the entire bottom of the binding pocket (Figure 7). Moreover, the candidate compound shared the hydrogen bond with Thr276 as well as interactions with Ala233, Pro274, Leu275 and Pro360 with paclitaxel (Supporting Information S2: Figure 9) [8, 55].

We also elucidated the binding of **2j** in the porcine beta‐tubulin cryo‐EM structure stabilised by paclitaxel (PDB: 7TQY) [56], to characterise the binding into this model, which was used for in vitro polymerisation assay, DLS, and MST. The docking score to porcine tubulin (7TQY) was analogous, with **2j** binding dG equal to –8.53 kcal/mol (compared with that of paclitaxel from re‐docking –10.78 kcal/mol). The candidate compound bound in a very similar pose into the taxane binding site, with its methyl group pointing to the hydrophobic pocket, but the whole steroid was rotated by approximately 90° compared with the pose in the human tubulin (Figure 7). Based on the comparison of the binding sites, it was concluded that the entrance into the porcine (7TQY) taxane binding site is narrower, caused by the two bulges originating from different orientations of His229/227 and Leu371/361 in human and porcine structure, respectively (Figure 7). Despite these structural differences, the interaction networks were analogous (Supporting Information S2: Figure 9), which supports the use of the porcine tubulin as a suitable model.

Although **2j** was found to favour the taxane site and aligned well with paclitaxel, we also intended to compare its binding with colchicine‐site binders, colchicine and 2‐methoxyestradiol. For this, we performed the docking of these three ligands into the colchicine binding site on tubulin from the co‐crystal structure with colchicine (PDB: 4O2B). It showed the binding of colchicine and 2‐methoxyestradiol with an extensive interaction network [57]. However, for **2j**, the modelled poses in the colchicine site displayed unfavourable binding, with clashes with conserved residues (Supporting Information S2: Figure 10). These findings further confirmed that the novel A‐ring‐fused isoxazoles of DHT preferentially bind into the taxane site.

### 2.6 Candidate Compounds Block the Mitosis and Decrease Viability of Docetaxel‐Resistant Prostate Cancer Cells

The current taxane therapy is limited by a frequent onset of acquired resistance mediated by the upregulation of efflux pumps or taxane‐metabolising enzymes, prosurvival pathways, altered microtubule regulatory proteins, and others [58, 59], which all limit the therapeutic use of taxane tubulin modulators. Moreover, it was found that in PCa, the glucocorticoid receptor is directly involved in the acquisition of docetaxel resistance [60].

Interestingly, all three active compounds (**2j**, **2g**, **2h**) demonstrated retained activity in docetaxel‐resistant DU145 (DU145‐DR), whereas paclitaxel and docetaxel exhibited 100‐fold and more than 2000‐fold reduction in efficacy, respectively (Figure 8). Furthermore, we also analysed cell‐cycle distribution in DU145 and DU145‐DR upon treatment with **2j**, docetaxel and paclitaxel. All compounds showed significant G2/M block only in parental DU145, while DU145‐DR was responsive only to **2j** treatment (see Figure 8), consistent with mitotic markers (cyclin B level, phosphorylation of CDK1 and Histone H3) analysed by immunoblotting (Supporting Information S2: Figure 11). Comparable effects were obtained using the colony formation assay established in both cell lines over a 10‐day period (Figure 8). Two other cancer cell lines, C4‐2 and HeLa, showed marked blockage of colony formation upon the analogous treatment as well (Supporting Information S2: Figure 12). Overall, the described results make our lead compound an ideal scaffold for further development of potential therapeutics for the treatment of cancer, including taxane‐resistant tumours.

## 3 Conclusions

We have previously demonstrated that some A‐ring‐substituted and heterocycle‐fused derivatives of DHT acted as potent AR antagonists [37, 46], selectively blocking AR transcription programme in prostate cancer cells, including castration‐resistant models. In this respect, A‐ring‐fused pyrazoles obtained from 2‐arylidene and 2‐ethylidene DHT derivatives by oxidative heterocyclisation proved to be the most active agents [36]. The current follow‐up project describes the synthesis and characterisation of novel A‐ring‐fused isoxazoles of DHT, achieved through iodine‐mediated oxidative cyclisation of α,β‐unsaturated oximes derived from DHT‐enones. The 10 new steroid‐based compounds are an addition to our library of previously described DHT‐derived A‐ring‐fused heterocycles. The structure of all new derivatives was experimentally confirmed by NMR and HRMS measurements, and the compounds were further evaluated for their biological activity. Novel compounds did not show any AR modulation activity but demonstrated unexpected antiproliferative activity (in prostate cancer and cervix cancer cell lines), with GI_50_ values around 5 µM, selectively inhibiting the growth of fast proliferating cancer cells while sparing noncancerous ones. Mechanistic studies performed on lead compound **2j** revealed that novel derivatives acted as tubulin stabilisers, increasing the rate of tubulin polymerisation and inducing mitotic arrest in low micromolar concentrations. Interestingly, the strong G2/M block induced upon short treatment was reversible but it led to apoptosis upon longer treatment. Molecular docking and interaction studies indicated that the novel compounds bind preferentially to the taxane site on tubulin by conserved interactions with the nanomolar *K*_D_ values. Importantly, the candidate compounds maintained their activity against docetaxel‐resistant prostate cancer cells (DU145), highlighting their potential to overcome resistance mechanisms associated with current taxane therapies. Finally, our findings suggest that A‐ring‐fused isoxazoles of DHT containing a smaller alkyl or five‐membered heteroaryl moiety on their *N*,*O*‐ring are promising tubulin stabilisers worthy of further development and optimisation for potential therapeutic use in cancer treatment, particularly in resistant cases. Further structural optimisation, such as alternative substitutions on the isoxazole ring or scaffold modifications guided by binding data, along with in vivo evaluation in relevant cancer models are possible future next steps.

## 4 Experimental

### 4.1 Chemistry

#### 4.1.1 General

Commercial vendors (Alfa Aesar, Sigma‐Aldrich and TCI) provided all chemicals, reagents and solvents, which were used without additional purification. A CEM Discover SP laboratory MW reactor with a maximum power of 200 W was utilised for MW‐assisted syntheses (with a dynamic control programme running). A PerkinElmer CHN analyzer model 2400 was used to collect data for elementary analysis. Kieselgel‐G plates (Si 254 F, Merck) with a thickness of 0.25 mm were used for TLC monitoring of the reactions. The compound spots were visualised by spraying 5% phosphomolybdic acid in 50% aqueous phosphoric acid. Silica gel 60, 40–63 μm (Merck), was used for column chromatography (CC). The uncorrected melting points (Mp) were measured using an SRS Optimelt digital device. Using the remaining solvent signal as an internal reference, NMR spectra were recorded in CDCl_3_ at room temperature using a Bruker DRX 500 instrument. Coupling constants (*J*) are expressed in Hz, and chemical shifts are reported in ppm (*δ* scale). Singlets (s), doublets (d), double doublets (dd), triplets (t), or multiplets (m) are used to represent the ^1^H signals’ multiplicities. The APT pulse sequence was utilised for multiplicity editing (positive signals are produced by CH_3_ and CH carbons, while negative signals are produced by CH_2_ and C carbons), and the ^13^C NMR spectra are ^1^H‐decoupled.

LC/MS analyses were carried out using the UPLC‐MS system comprising an Acquity UPLC chromatograph with a photodiode array detector and a single quadrupole mass spectrometer (Waters), equipped with a C18 X‐Select HSS T3 column (2.5 µm, 3.0 mm × 50 mm), operated at 30°C with a flow rate of 0.6 mL/min. The mobile phase was (A) 0.01 M ammonium acetate (AmAc) in H2O and (B) CH3CN, with a linearly programmed gradient elution. HRMS analysis was performed using LC‐MS (Dionex Ultimate 3000, Thermo Fischer Scientific, USA) with an Exactive Plus Orbitrap high‐resolution mass spectrometer (Thermo Exactive plus, Thermo Fischer Scientific, USA) operating at positive or negative full scan mode (120,000 FWMH) in the range of 100–1000 *m/z* with electrospray ionisation operating at 150°C and the source voltage of 3.6 kV.

The InChI codes of the investigated compounds, together with some biological activity data, are provided as Supporting Information.

#### 4.1.2 Synthesis of the A‐Ring‐Modified α,β‐Enones

Compounds **1a**–**h**, **1j** were synthesised as described previously [36, 37, 38]. The preparation and chemical characterisation of the compounds **2a**–**2j** were simultaneously described in the PhD thesis of the first author [61].

17β‐Hydroxy‐2‐(pyridin‐2‐ylidene)‐5α‐androstan‐3‐one (**1i**): According to the general method described previously [37], pyridine‐2‐carbaldehyde (342 µL) was used for the reaction. The solution was stirred at room temperature for 3 h. The crude product was purified by CC (silica gel, EtOAc/CH_2_Cl_2_ = 10:90 to EtOAc/CH_2_Cl_2_ = 20:80 using gradient elution). Beige solid. Yield: 785 mg (69%); Mp 179°C–182°C; ^1^H NMR (CDCl_3_, 500 MHz): *δ*_H_ 0.75 (s, 3H, 18‐H_3_), 0.84 (s, 3H, 19‐H_3_), 0.87–1.03 (overlapping m, 3H), 1.13 (m, 1H), 1.23–1.31 (overlapping m, 2H), 1.36–1.49 (overlapping m, 5H), 1.62 (m, 1H), 1.72–1.87 (overlapping m, 4H), 2.07 (m, 1H), 2.21–2.30 (overlapping d and dd, 2H, one of 1‐H_2_ and one of 4‐H_2_), 2.47 (dd, 1H, *J* = 18.5 Hz, *J* = 5.2 Hz, the other of 4‐H_2_), 3.66 (t, 1H, *J* = 8.4 Hz, 17‐H), 3.88 (d, 1H, J = 16.7 Hz, the other of 1‐H_2_), 7.17 (t‐like m, 1H, 5′‐H), 7.36 (d, 1H, *J* = 7.8 Hz, 3′‐H), 7.42 (s, 1H, 2a‐H), 7.68 (t‐like m, 1H, 4′‐H), 8.69 (d, 1H, *J* = 4.4 Hz, 6′‐H); ^13^C NMR (CDCl_3_, 125 MHz): *δ*_C_ 11.2 (C‐18), 12.2 (C‐19), 21.2 (CH_2_), 23.6 (CH_2_), 28.8 (CH_2_), 30.8 (CH_2_), 31.3 (CH_2_), 35.7 (CH), 36.2 (C‐10), 36.9 (CH_2_), 42.0 (CH_2_), 42.8 (CH), 43.1 (CH_2_), 43.2 (C‐13), 51.2 (CH), 54.0 (CH), 82.1 (C‐17), 122.5 (C‐3′), 127.0 (C‐5′), 134.3 (C‐2a), 136.2 (C‐4′), 139.4 (C‐2), 149.7 (C‐6′), 155.6 (C‐2′), 202.0 (C‐3); Exact *m/z* calculated for [M + H]^+^ C_25_H_34_NO_2_: 380.2584, HRMS (*m/z*) [M + H]^+^ measured: 380.2584; Anal. Calcd. for C_25_H_33_NO_2_ C 79.11; H 8.76. Found C 79.25; H 8.74.

#### 4.1.3 General Procedure for the Synthesis of DHT‐Derived A‐Ring‐Fused Isoxazoles

The appropriate arylidene (**1a**–**f**), heteroarylidene (**1g**–**i**), or ethylidene derivative (**1j**) (0.50 mmol) and hydroxylamine hydrochloride (0.75 mmol, 1.50 equiv.) were dissolved in abs. EtOH (10 mL), and sodium acetate (1.00 mmol, 2.00 equiv.) was added. The mixture was heated at reflux temperature for 2 h. Then, the reaction mixture was cooled to room temperature, poured into water (20 mL), and the aqueous phase was extracted with EtOAc (3 × 5 mL). The combined organic layer was washed with water (2 × 10 mL) and brine (10 mL), dried over anhydrous Na_2_SO_4_ and the solvent was evaporated under reduced pressure to yield a yellow solid. Then, the solid was dissolved in DMSO (5 mL), and I_2_ (0.60 mmol, 1.20 equiv.) and K_2_CO_3_ (3 equiv.) were added. The reaction mixture was heated to 60°C for 1 h. After the completion of the reaction (TLC), it was cooled to room temperature, poured into a saturated solution of Na_2_S_2_O_3_ (20 mL) and then extracted with EtOAc (3 × 5 mL). The combined organic layer was washed with water (2 × 10 mL) and brine (10 mL), dried over anhydrous Na_2_SO_4_ and the solvent was evaporated under reduced pressure to yield a brown solid, which was then purified by CC with a solvent mixture described in each subchapter, followed by recrystallisation from MeOH.

17β‐Hydroxy‐5′‐phenylizoxazolo[3′,4′:3,2]‐5α‐androstane (**2a**): According to Section 4.1.2, 189 mg of **1a** was used. The crude product was purified by CC (EtOAc/CH_2_Cl_2_ = 2:98). White solid. Yield: 122 mg (62%). Mp 276°C–279°C; ^1^H NMR (CDCl_3_, 500 MHz): *δ*_H_ 0.77 (s, 3H, 18‐H_3_), 0.78 (s, 3H, 19‐H_3_), 0.89–1.03 (overlapping m, 3H, 9α‐H, 7α‐H and 14α‐H), 1.15 (m, 1H, 12α‐H), 1.25–1.51 (overlapping m, 5H, 15β‐H, 11β‐H, 6β‐H, 8β‐H and 16β‐H), 1.56–1.78 (overlapping m, 5H, 11α‐H, 5α‐H, 15α‐H, 6α‐H and 7β‐H), 1.89 (m, 1H, 12β‐H), 2.08 (m, 1H, 16α‐H), 2.29 (d, 1H, *J* = 15.6 Hz, 1α‐H), 2.39 (dd, 1H, *J* = 17.3 Hz, *J* = 12.6 Hz, 4β‐H), 2.79 (dd, 1H, *J* = 17.3 Hz, *J* = 5.2 Hz, 4α‐H), 2.86 (d, 1H, *J* = 15.6 Hz, 1β‐H), 3.67 (t, 1H, *J* = 8.6 Hz, 17α‐H), 7.40 (t‐like m, 1H, 4″‐H), 7.47 (t‐like m, 2H, 3″‐H and 5″‐H), 7.81 (d‐like m, 2H, 2″‐H and 6″‐H); ^13^C NMR (CDCl_3_, 125 MHz): *δ*_C_ 11.2 (C‐18), 11.9 (C‐19), 21.0 (C‐11), 23.6 (C‐15), 25.7 (C‐4), 29.2 (C‐6), 30.7 (C‐16), 31.3 (C‐7), 35.3 (C‐1), 35.9 (C‐8), 36.4 (C‐10), 36.8 (C‐12), 41.4 (C‐5), 43.0 (C‐13), 51.0 (C‐14), 54.0 (C‐9), 82.0 (C‐17), 110.5 (C‐2), 126.2 (2 C, C‐2″ and C‐6″), 128.9 (C‐1″), 129.0 (2 C, C‐3″ and C‐5″), 129.3 (C‐4″), 161.1 (C‐3), 163.3 (C‐5′); Exact *m/z* calculated *for* [M + H]^+^ C_26_H_34_NO_2_ *:* 392.2585, HRMS *(m/z)* [M + H]^+^ measured*:* 392.2584; Anal. Calcd. for C_26_H_33_NO_2_ C 79.76; H 8.50. Found C 79.89; H 8.42.

17β‐Hydroxy‐5′‐(4″‐tolyl)‐izoxazolo[3′,4′:3,2]‐5α‐androstane (**2b**): According to Section 4.2.2, 196 mg of **1b** was used. The crude product was purified by CC (CH_2_Cl_2_). White solid. Yield: 135 mg (67%). Mp 231°C–233°C; ^1^H NMR (CDCl_3_, 500 MHz): *δ*_H_ 0.77 (s, 3H, 18‐H_3_), 0.78 (s, 3H, 19‐H_3_), 0.88–1.03 (overlapping m, 3H, 9α‐H, 7α‐H and 14α‐H), 1.15 (m, 1H, 12α‐H), 1.25–1.51 (overlapping m, 5H, 15β‐H, 11β‐H, 6β‐H, 8β‐H and 16β‐H), 1.55–1.78 (overlapping m, 5H, 11α‐H, 5α‐H, 15α‐H, 6α‐H and 7β‐H), 1.89 (m, 1H, 12β‐H), 2.08 (m, 1H, 16α‐H), 2.27 (d, 1H, *J* = 15.6 Hz, 1α‐H), 2.38 (dd, 1H, *J* = 17.1 Hz, *J* = 12.4 Hz, 4β‐H), 2.40 (s, 3H, 4″‐CH_3_), 2.78 (dd, 1H, *J* = 17.3 Hz, *J* = 5.2 Hz, 4α‐H), 2.83 (d, 1H, *J* = 15.6 Hz, 1β‐H), 3.67 (t, 1H, *J* = 8.6 Hz, 17α‐H), 7.28 (d, 2H, *J* = 8.0 Hz, 3″‐H and 5″‐H), 7.61 (d, 2H, *J* = 8.2 Hz, 2″‐H and 6″‐H); ^13^C NMR (CDCl_3_, 125 MHz): *δ*_C_ 11.2 (C‐18), 11.9 (C‐19), 21.0 (C‐11), 21.6 (4″‐CH_3_), 23.6 (C‐15), 25.7 (C‐4), 29.2 (C‐6), 30.7 (C‐16), 31.3 (C‐7), 35.3 (C‐1), 35.9 (C‐8), 36.4 (C‐10), 36.9 (C‐12), 41.4 (C‐5), 43.0 (C‐13), 51.1 (C‐14), 54.1 (C‐9), 82.0 (C‐17), 109.9 (C‐2), 126.1 (2 C, C‐2″ and C‐6″), 126.2 (C‐1″), 129.7 (2 C, C‐3″ and C‐5″), 139.5 (C‐4″), 161.0 (C‐3), 163.6 (C‐5′); Exact *m/z* calculated *for* [M + H]^+^ C_27_H_36_NO_2_ *:* 406.2741, HRMS *(m/z)* [M + H]^+^ measured*:* 406.2741; Anal. Calcd. for C_27_H_35_NO_2_ C 79.96; H 8.70. Found C 80.09; H 8.62.

17β‐Hydroxy‐5′‐(4″‐methoxyphenyl)‐izoxazolo[3′,4′:3,2]‐5α‐androstane (**2c**): According to Section 4.2.2, 204 mg of **1c** was used. The crude product was purified by CC (EtOAc/CH_2_Cl_2_ = 2:98). White solid. Yield: 134 mg (64%). Mp 222°C–224°C; ^1^H NMR (CDCl_3_, 500 MHz): *δ*_H_ 0.77 (s, 3H, 18‐H_3_), 0.78 (s, 3H, 19‐H_3_), 0.88–1.03 (overlapping m, 3H, 9α‐H, 7α‐H and 14α‐H), 1.15 (m, 1H, 12α‐H), 1.25–1.48 (overlapping m, 5H, 15β‐H, 11β‐H, 6β‐H, 8β‐H and 16β‐H), 1.57–1.77 (overlapping m, 5H, 11α‐H, 5α‐H, 15α‐H, 6α‐H and 7β‐H), 1.89 (m, 1H, 12β‐H), 2.08 (m, 1H, 16α‐H), 2.26 (d, 1H, *J* = 15.5 Hz, 1α‐H), 2.37 (dd, 1H, *J* = 17.3 Hz, *J* = 12.6 Hz, 4β‐H), 2.75–2.83 (overlapping dd and d, 2H, 4α‐H and 1β‐H), 3.67 (t, 1H, *J* = 8.6 Hz, 17α‐H), 3.85 (s, 3H, 4″‐OMe), 7.00 (d, 2H, *J* = 8.8 Hz, 3″‐H and 5″‐H), 7.67 (d, 2H, *J* = 8.8 Hz, 2″‐H and 6″‐H); ^13^C NMR (CDCl_3_, 125 MHz): *δ*_C_ 11.2 (C‐18), 11.9 (C‐19), 21.0 (C‐11), 23.6 (C‐15), 25.7 (C‐4), 29.2 (C‐6), 30.7 (C‐16), 31.3 (C‐7), 35.3 (C‐1), 35.9 (C‐8), 36.4 (C‐10), 36.9 (C‐12), 41.4 (C‐5), 43.0 (C‐13), 51.1 (C‐14), 54.1 (C‐9), 55.5 (4″‐OMe), 82.0 (C‐17), 109.1 (C‐2), 114.5 (2 C, C‐3″ and C‐5″), 121.8 (C‐1″), 127.7 (2 C, C‐2″ and C‐6″), 160.4 (C‐4″), 161.0 (C‐3), 163.4 (C‐5′); Exact *m/z* calculated *for* [M + H]^+^ C_27_H_36_NO_3_ *:* 422.2690, HRMS *(m/z)* [M + H]^+^ measured*:* 422.2690; Anal. Calcd. for C_27_H_35_NO_3_ C 76.92; H 8.37. Found C 76.98; H 8.36.

17β‐Hydroxy‐5′‐(4″‐fluorophenyl)‐izoxazolo[3′,4′:3,2]‐5α‐androstane (**2d**): According to Section 4.2.2, 198 mg of **1d** was used. The crude product was purified by CC (EtOAc/CH_2_Cl_2_ = 2:98). White solid. Yield: 140 mg (68%). Mp 209°C–211°C; ^1^H NMR (CDCl_3_, 500 MHz): *δ*_H_ 0.77 (s, 3H, 18‐H_3_), 0.79 (s, 3H, 19‐H_3_), 0.89–1.03 (overlapping m, 3H, 9α‐H, 7α‐H and 14α‐H), 1.15 (m, 1H, 12α‐H), 1.25–1.52 (overlapping m, 5H, 15β‐H, 11β‐H, 6β‐H, 8β‐H and 16β‐H), 1.56–1.79 (overlapping m, 5H, 11α‐H, 5α‐H, 15α‐H, 6α‐H and 7β‐H), 1.89 (m, 1H, 12β‐H), 2.08 (m, 1H, 16α‐H), 2.27 (d, 1H, *J* = 15.6 Hz, 1α‐H), 2.39 (dd, 1H, *J* = 17.3 Hz, *J* = 12.6 Hz, 4β‐H), 2.79 (dd, 1H, *J* = 17.3 Hz, *J* = 5.3 Hz, 4α‐H), 2.81 (d, 1H, *J* = 15.5 Hz, 1β‐H), 3.67 (t, 1H, *J* = 8.6 Hz, 17α‐H), 7.17 (t, 2H, *J* = 8.7 Hz, 3″‐H, 5″‐H), 7.71 (dd, 2H, *J* = 8.8 Hz, *J* = 5.3 Hz, 2″‐H and 6″‐H); ^13^C NMR (CDCl_3_, 125 MHz): *δ*_C_ 11.2 (C‐18), 12.0 (C‐19), 21.0 (C‐11), 23.6 (C‐15), 25.6 (C‐4), 29.2 (C‐6), 30.7 (C‐16), 31.3 (C‐7), 35.3 (C‐1), 35.9 (C‐8), 36.4 (C‐10), 36.8 (C‐12), 41.4 (C‐5), 43.0 (C‐13), 51.0 (C‐14), 54.0 (C‐9), 82.0 (C‐17), 110.2 (C‐2), 116.2 (d, 2 C, *J* = 21.9 Hz, C‐3″ and C‐5″), 125.2 (d, 1 C, *J* = 3.4 Hz, C‐1″), 128.2 (d, 2 C, *J* = 8.4 Hz, C‐2″ and C‐6″), 161.2 (C‐3), 162.5 (C‐5′), 163.2 (d, *J* = 250.3 Hz, C‐4″); Exact *m/z* calculated *for* [M + H]^+^ C_26_H_33_FNO_2_ *:* 410.2490, HRMS *(m/z)* [M + H]^+^ measured*:* 410.2490; Anal. Calcd. for C_26_H_32_FNO_2_ C 76.25; H 7.88. Found C 76.32; H 7.87.

17β‐Hydroxy‐5′‐(4″‐chlorophenyl)‐izoxazolo[3′,4′:3,2]‐5α‐androstane (**2e**): According to Section 4.2.2, 207 mg of **1e** was used. The crude product was purified by CC (EtOAc/CH_2_Cl_2_ = 5:95). White solid. Yield: 152 mg (71%). Mp 241°C–243°C; ^1^H NMR (CDCl_3_, 500 MHz): *δ*_H_ 0.78 (s, 3H, 18‐H_3_), 0.79 (s, 3H, 19‐H_3_), 0.89–1.04 (overlapping m, 3H, 9α‐H, 7α‐H and 14α‐H), 1.15 (m, 1H, 12α‐H), 1.26–1.50 (overlapping m, 5H, 15β‐H, 11β‐H, 6β‐H, 8β‐H and 16β‐H), 1.56–1.79 (overlapping m, 5H, 11α‐H, 5α‐H, 15α‐H, 6α‐H and 7β‐H), 1.90 (m, 1H, 12β‐H), 2.08 (m, 1H, 16α‐H), 2.27 (d, 1H, *J* = 15.6 Hz, 1α‐H), 2.40 (dd, 1H, *J* = 17.3 Hz, *J* = 12.6 Hz, 4β‐H), 2.77–2.83 (overlapping dd and d, 2H, 4α‐H and 1β‐H), 3.67 (m, 1H, 17α‐H), 7.45 (d, 2H, *J* = 8.4 Hz, 3″‐H and 5″‐H), 7.65 (d, 2H, *J* = 8.4 Hz, 2″‐H and 6″‐H); ^13^C NMR (CDCl_3_, 125 MHz): *δ*_C_ 11.2 (C‐18), 12.0 (C‐19), 21.1 (C‐11), 23.6 (C‐15), 25.7 (C‐4), 29.2 (C‐6), 30.8 (C‐16), 31.3 (C‐7), 35.4 (C‐1), 36.0 (C‐8), 36.4 (C‐10), 36.9 (C‐12), 41.5 (C‐5), 43.0 (C‐13), 51.1 (C‐14), 54.1 (C‐9), 82.0 (C‐17), 110.9 (C‐2), 127.4 (C‐1″),127.4 (2 C, C‐2″ and C‐6″), 129.3 (2 C, C‐3″ and C‐5″), 135.4 (C‐4″), 161.2 (C‐3), 162.4 (C‐5′); Exact *m/z* calculated *for* [M + H]^+^ C_26_H_33_ClNO_2_ *:* 426.2194, HRMS *(m/z)* [M + H]^+^ measured*:* 426.2194; Anal. Calcd. for C_26_H_32_ClNO_2_ C 73.31; H 7.57. Found C 73.45; H 7.54.

17β‐Hydroxy‐5′‐(4″‐bromophenyl)‐izoxazolo[3′,4′:3,2]‐5α‐androstane (**2f**): According to Section 4.2.2, 229 mg of **1 f** was used. The crude product was purified by CC (EtOAc/CH_2_Cl_2_ = 2:98). White solid. Yield: 143 mg (61%). Mp 253°C–255°C; ^1^H NMR (CDCl_3_, 500 MHz): *δ*_H_ 0.78 (s, 3H, 18‐H_3_), 0.78 (s, 3H, 19‐H_3_), 0.88–1.03 (overlapping m, 3H, 9α‐H, 7α‐H and 14α‐H), 1.15 (m, 1H, 12α‐H), 1.26–1.49 (overlapping m, 5H, 15β‐H, 11β‐H, 6β‐H, 8β‐H and 16β‐H), 1.56–1.72 (overlapping m, 4H, 11α‐H, 5α‐H, 15α‐H and 6α‐H), 1.77 (m, 1H, 7β‐H), 1.90 (m, 1H, 12β‐H), 2.09 (m, 1H, 16α‐H), 2.26 (d, 1H, *J* = 15.6 Hz, 1α‐H), 2.39 (dd, 1H, *J* = 17.3 Hz, *J* = 12.6 Hz, 4β‐H), 2.77–2.82 (overlapping dd and d, 2H, 4α‐H and 1β‐H), 3.67 (t, 1H, *J* = 8.5 Hz, 17α‐H), 7.60 (overlapping m, 4H, 3″‐H, 5″‐H, 2″‐H and 6″‐H); ^13^C NMR (CDCl_3_, 125 MHz): *δ*_C_ 11.2 (C‐18), 12.0 (C‐19), 21.1 (C‐11), 23.6 (C‐15), 25.7 (C‐4), 29.2 (C‐6), 30.8 (C‐16), 31.3 (C‐7), 35.4 (C‐1), 36.0 (C‐8), 36.4 (C‐10), 36.9 (C‐12), 41.5 (C‐5), 43.0 (C‐13), 51.1 (C‐14), 54.1 (C‐9), 82.1 (C‐17), 111.0 (C‐2), 123.7 (C‐4″), 127.7 (2 C, C‐2″ and C‐6″), 127.8 (C‐1″), 132.3 (2 C, C‐3″ and C‐5″), 161.2 (C‐3), 162.4 (C‐5′); Exact *m/z* calculated *for* [M + H]^+^ C_26_H_33_BrNO_2_ *:* 470.1690, HRMS *(m/z)* [M + H]^+^ measured*:* 470.1689; Anal. Calcd. for C_26_H_32_BrNO_2_ C 66.38; H 6.86. Found C 66.49; H 6.83.

17β‐Hydroxy‐5′‐(furan‐2″‐yl)‐izoxazolo[3′,4′:3,2]‐5α‐androstane (**2 g**): According to Section 4.2.2, 184 mg of **1 g** was used. The crude product was purified by CC (EtOAc/CH_2_Cl_2_ = 2:98). White solid. Yield: 103 mg (54%). Mp > 220°C (decomp.); ^1^H NMR (CDCl_3_, 500 MHz): *δ*_H_ 0.78 (s, 3H, 18‐H_3_), 0.79 (s, 3H, 19‐H_3_), 0.85–1.02 (overlapping m, 3H, 9α‐H, 7α‐H and 14α‐H), 1.14 (m, 1H, 12α‐H), 1.25–1.52 (overlapping m, 5H, 15β‐H, 11β‐H, 6β‐H, 8β‐H and 16β‐H), 1.54–1.67 (overlapping m, 3H, 11α‐H, 5α‐H and 15α‐H), 1.73–1.76 (overlapping m, 2H, 6α‐H and 7β‐H), 1.89 (m, 1H, 12β‐H), 2.08 (m, 1H, 16α‐H), 2.20 (d, 1H, *J* = 16.2 Hz, 1α‐H), 2.36 (dd, 1H, *J* = 17.2 Hz, *J* = 12.6 Hz, 4β‐H), 2.76 (dd, 1H, *J* = 17.3 Hz, *J* = 5.1 Hz, 4α‐H), 2.93 (d, 1H, *J* = 16.2 Hz, 1β‐H), 3.66 (t, 1H, *J* = 8.4 Hz, 17α‐H), 6.52 (m, 1H, 4″‐H), 6.78 (d, 1H, *J* = 3.2 Hz, 3″‐H), 7.55 (s, 1H, 5″‐H); ^13^C NMR (CDCl_3_, 125 MHz): *δ*_C_ 11.2 (C‐18), 12.0 (C‐19), 21.0 (C‐11), 23.6 (C‐15), 25.5 (C‐4), 29.2 (C‐6), 30.7 (C‐16), 31.3 (C‐7), 34.0 (C‐1), 35.9 (C‐8), 36.1 (C‐10), 36.8 (C‐12), 41.7 (C‐5), 43.0 (C‐13), 51.0 (C‐14), 54.0 (C‐9), 82.0 (C‐17), 109.6 (C‐4″), 110.3 (C‐2), 111.8 (C‐3″), 143.7 (C‐5″), 144.7 (C‐2″), 156.1 (C‐3), 160.7 (C‐5′); Exact *m/z* calculated *for* [M + H]^+^ C_24_H_32_NO_3_ *:* 382.2377, HRMS *(m/z)* [M + H]^+^ measured*:* 382.2377; Anal. Calcd. for C_24_H_31_NO_3_ C 75.56; H 8.19. Found C 75.62; H 8.17.

17β‐Hydroxy‐5′‐(tiophen‐2″‐yl)‐izoxazolo[3′,4′:3,2]‐5α‐androstane (**2h**): According to Section 4.2.2, 192 mg of **1 h** was used. The crude product was purified by CC (EtOAc/CH_2_Cl_2_ = 5:95). White solid. Yield: 134 mg (67%). Mp 247°C–249°C; ^1^H NMR (CDCl_3_, 500 MHz): *δ*_H_ 0.78 (s, 3H, 18‐H_3_), 0.80 (s, 3H, 19‐H_3_), 0.88–1.03 (overlapping m, 3H, 9α‐H, 7α‐H and 14α‐H), 1.15 (m, 1H, 12α‐H), 1.25–1.50 (overlapping m, 5H, 15β‐H, 11β‐H, 6β‐H, 8β‐H and 16β‐H), 1.55–1.78 (overlapping m, 5H, 11α‐H, 5α‐H, 15α‐H, 6α‐H and 7β‐H), 1.89 (m, 1H, 12β‐H), 2.08 (m, 1H, 16α‐H), 2.18 (d, 1H, *J* = 15.8 Hz, 1α‐H), 2.37 (dd, 1H, *J* = 17.3 Hz, *J* = 12.6 Hz, 4β‐H), 2.74–2.81 (overlapping dd and d, 2H, 4α‐H and 1β‐H), 3.67 (t, 1H, *J* = 8.6 Hz, 17α‐H), 7.15 (dd, 1H, *J* = 4.9 Hz, *J* = 3.8 Hz, 4″‐H), 7.45 (overlapping m, 2H, 3″‐H and 5″‐H); ^13^C NMR (CDCl_3_, 125 MHz): *δ*_C_ 11.2 (C‐18), 12.1 (C‐19), 21.0 (C‐11), 23.6 (C‐15), 25.6 (C‐4), 29.2 (C‐6), 30.7 (C‐16), 31.3 (C‐7), 34.7 (C‐1), 35.9 (C‐8), 36.4 (C‐10), 36.8 (C‐12), 41.6 (C‐5), 43.0 (C‐13), 51.0 (C‐14), 54.0 (C‐9), 82.0 (C‐17), 109.9 (C‐2), 125.9 (C‐4″), 127.2 (C‐5″), 127.9 (C‐3″), 130.4 (C‐2″), 159.4 (C‐3), 161.0 (C‐5′); Exact *m/z* calculated *for* [M + H]^+^ C_24_H_32_NO_2_S 398.2149, HRMS *(m/z)* [M + H]^+^ measured*:* 398.2148; Anal. Calcd. for C_24_H_31_NO_2_S C 72.51; H 7.86. Found C 72.63; H 7.81.

17β‐Hydroxy‐5′‐(pyridin‐2″‐yl)‐izoxazolo[3′,4′:3,2]‐5α‐androstane (**2i**): According to Section 4.2.2, 190 mg of **1i** was used. The crude product was purified by CC (EtOAc/CH_2_Cl_2_ = 10:90). White solid. Yield: 108 mg (55%). Mp 242°C–245°C; ^1^H NMR (CDCl_3_, 500 MHz): *δ*_H_ 0.78 (s, 3H, 18‐H_3_), 0.80 (s, 3H, 19‐H_3_), 0.89–1.04 (overlapping m, 3H, 9α‐H, 7α‐H and 14α‐H), 1.15 (m, 1H, 12α‐H), 1.25–1.52 (overlapping m, 5H, 15β‐H, 11β‐H, 6β‐H, 8β‐H and 16β‐H), 1.56–1.69 (overlapping m, 3H, 11α‐H, 5α‐H and 15α‐H), 1.73–1.84 (overlapping m, 2H, 6α‐H and 7β‐H), 1.88 (m, 1H, 12β‐H), 2.08 (m, 1H, 16α‐H), 2.32 (d, 1H, *J* = 16.8 Hz, 1α‐H), 2.41 (dd, 1H, *J* = 17.3 Hz, *J* = 12.6 Hz, 4β‐H), 2.80 (dd, 1H, *J* = 17.3 Hz, *J* = 5.2 Hz, 4α‐H), 3.27 (d, 1H, *J* = 16.8 Hz, 1β‐H), 3.67 (t, 1H, *J* = 8.6 Hz, 17α‐H), 7.25 (m, 1H, 5″‐H), 7.78 (t‐like m, 1H, 4″‐H), 7.85 (d, 1H, *J* = 7.9 Hz, 3″‐H), 8.69 (d, 1H, *J* = 5.4 Hz, 6″‐H); ^13^C NMR (CDCl_3_, 125 MHz): *δ*_C_ 11.2 (C‐18), 12.0 (C‐19), 21.0 (C‐11), 23.6 (C‐15), 25.7 (C‐4), 29.2 (C‐6), 30.7 (C‐16), 31.3 (C‐7), 35.4 (C‐1), 35.9 (C‐8), 36.1 (C‐10), 36.9 (C‐12), 41.5 (C‐5), 43.0 (C‐13), 51.1 (C‐14), 54.0 (C‐9), 82.0 (C‐17), 114.1 (C‐2), 121.4 (C‐3″), 123.3 (C‐5″), 136.8 (C‐4″), 148.6 (C‐2″), 149.9 (C‐6″), 161.6 (C‐3), 162.1 (C‐5′); Exact *m/z* calculated *for* [M + H]^+^ C_25_H_33_N_2_O_2_:393.2537, HRMS *(m/z)* [M + H]^+^ measured: 393.2537; Anal. Calcd. for C_25_H_32_N_2_O_2_ C 76.49; H 8.22. Found C 76.62; H 8.18.

17β‐Hydroxy‐5′‐methylizoxazolo[3′,4′:3,2]‐5α‐androstane (**2j**): According to Section 4.2.2, 158 mg of **1j** was used. The crude product was purified by CC (EtOAc/CH_2_Cl_2_ = 2:98). White solid. Yield: 90 mg (55%). Mp 210°C–213°C; ^1^H NMR (CDCl_3_, 500 MHz): *δ*_H_ 0.74 (s, 3H, 18‐H_3_), 0.77 (s, 3H, 19‐H_3_), 0.82–1.01 (overlapping m, 3H, 9α‐H, 7α‐H and 14α‐H), 1.11 (m, 1H, 12α‐H), 1.24–1.53 (overlapping m, 5H, 15β‐H, 11β‐H, 6β‐H, 8β‐H and 16β‐H), 1.57–1.67 (overlapping m, 4H, 11α‐H, 5α‐H, 15α‐H and 6α‐H), 1.74 (m, 1H, 7β‐H), 1.86 (m, 1H, 12β‐H), 1.94 (d, 1H, *J* = 15.3 Hz, 1α‐H), 2.08 (m, 1H, 16α‐H), 2.26–2.32 (overlapping dd and s, 4H, 4β‐H and 5′‐CH_3_), 2.49 (d, 1H, *J* = 15.2 Hz, 1β‐H), 2.70 (dd, 1H, *J* = 17.3 Hz, *J* = 5.1 Hz, 4α‐H), 3.65 (t, 1H, *J* = 8.2 Hz, 17α‐H); ^13^C NMR (CDCl_3_, 125 MHz): *δ*_C_ 11.1 (5′‐CH_3_), 11.2 (C‐18), 11.8 (C‐19), 20.9 (C‐11), 23.6 (C‐15), 25.6 (C‐4), 29.2 (C‐6), 30.7 (C‐16), 31.3 (C‐7), 33.2 (C‐1), 35.9 (C‐8), 36.2 (C‐10), 36.8 (C‐12), 41.8 (C‐5), 43.0 (C‐13), 51.1 (C‐14), 54.0 (C‐9), 82.0 (C‐17), 110.2 (C‐2), 161.4 (C‐3), 163.8 (C‐5′); Exact *m/z* calculated *for* [M + H]^+^ C_21_H_32_NO_2_: 330.2428, HRMS *(m/z)* [M + H]^+^ measured: 330.2428; Anal. Calcd. for C_21_H_31_NO_2_ C 76.55; H 9.48. Found C 76.65; H 9.45.

### 4.2 Biological Assays

#### 4.2.1 Cell Lines

The 22Rv1, C4‐2 and DU145 cells (all purchased from ECACC) and the docetaxel‐resistant DU145‐DR (kind gift from prof. Zoran Culig, Medical University Innsbruck, Austria) were grown in RPMI‐1640 medium. The HeLa (ECACC) and BJ cells (ATCC) were grown in DMEM. All media were supplemented with 10% foetal bovine serum, 100 µg/mL streptomycin, 100 IU/mL penicillin, 1 mM sodium pyruvate and 4 mM glutamine. Cells were cultivated in a 5% CO_2_ atmosphere, at 37°C in a humidified incubator.

#### 4.2.2 Cell Viability Assay

Cells were seeded in their appropriate media into the 96‐well tissue culture plates. The next day, compounds were added for 72 h. At the end of the treatment, the resazurin (Sigma Aldrich) was added for 4 h, with subsequent measurement of the fluorescence of resorufin at 544 nm/590 nm (excitation/emission) using a Fluoroskan Ascent microplate reader (Labsystems). The fluorescence signal was used to calculate the percentual viability.

Alternatively, a crystal violet‐based viability assay was recruited to analyse cell viability. Upon the treatment, cells undergoing death loose their adhesion and are washed away. The remaining adherent cells were stained by crystal violet dye (Merck, 5% solution in ethanol), which bound to proteins and DNA of living cells. After washing to remove excess dye, the crystal violet was solubilised in 1% SDS (Merck) and its absorbance was measured at 570 nm using (Tecan M200‐Pro, Biotek). The intensity of the colour directly correlated with the number of viable cells; hence, from the measured dose responses, GI_50_ values were calculated using GraphPad Prism 8.

#### 4.2.3 Immunoblotting

Cells were pelleted after treatments and washed with PBS and kept frozen at –80°C. Lysis in ice‐cold radioimmunoprecipitation assay (RIPA) buffer with additional protease and phosphatase inhibitors was performed using ultrasound sonication (10 s, 30% amplitude). Supernatants were centrifuged at 14,000 *g* for 30 min. Proteins in supernatants were measured, balanced and denatured in an SDS‐loading buffer by heating at 95°C. SDS‐PAGE‐separated proteins were electro‐blotted onto the nitrocellulose membrane. Membranes were blocked in 4% BSA and 0.1% Tween 20 in TBS and incubated overnight with primary antibodies, subsequently washed and incubated with secondary antibodies conjugated with peroxidase. Peroxidase activity was detected by SuperSignal West Pico reagents (Thermo Scientific) using a CCD camera RIPALAS‐4000 (FujiFilm). Primary antibodies were purchased from Cell Signaling Technology (PARP, clone 46D11; Cyclin B1, clone V152; CDK1 (cdc2), clone POH1; Histone H3, clone D1H2; Aurora A, clone 1G4; Aurora A pThr288/Aurora B pThr232/Aurora C pThr198, clone D13A11; polyclonal Caspase 7; Caspase 9, clone 9502; HSP 90, clone C45G5), Santa Cruz Biotechnology (β‐actin, clone C4; polyclonal CDK1 pThr161; Caspase 3, clone 31A1067) and Merck (α‐tubulin, clone DM1A; polyclonal Histone H3 pSer10; Histone H2A.X pSer139, clone JBW301). Secondary antibodies were purchased from Cell Signaling Technology as anti‐rabbit secondary antibodies (porcine anti‐rabbit immunoglobulin serum); anti‐mouse secondary antibodies (rabbit anti‐mouse IgG, clone D3V2A). Antibodies were diluted in 4% BSA and 0.1% Tween 20 in TBS.

#### 4.2.4 Cell‐Cycle Analysis

Cells were treated with compounds for the designed time, they were detached by trypsinisation, washed with PBS and fixed with 70% ethanol for storage. Subsequently, cells were rehydrated and permeabilised by 2 M HCl, 0.5% Triton X‐100. Neutralisation and washing with PBS were followed by staining the cellular DNA with propidium iodide and analysis by flow cytometry with a 488 nm laser (BD FACS Verse with BD FACSuite software, version 1.0.6.). Cell‐cycle distribution was analysed by ModFit LT (Verity Software House, version 5.0).

#### 4.2.5 Live Cell Imaging

Cells were cultivated in an 8‐well chamber µ‐Slide (IBIDI) for 24 h before treatment with compounds. The LionheartFX (Biotek) live cell imaging instrument was pre‐warmed to 37°C, and the proper conditions (humidification, 5% CO_2_ atmosphere) were ensured. Imaging was set into the brightfield mode with phase contrast (in the Gen5 software) into the centre of each well, and it was set to capture a photo every hour during the 24 h treatment. Treatments were captured in duplicate and then, representative replicates in particular time points were shown.

### 4.3 Molecular Docking

The molecular docking of the candidate compound **2j** and standards was conducted into different tubulin structures (cryo‐EM structure of the tubulin dimer extracted from HeLa cells, stabilised by paclitaxel (PDB: 62I2), porcine beta‐tubulin cryo‐EM structure stabilised by paclitaxel (PDB: 7TQY), co‐crystal structure with bound colchicine (PDB: 4O2B)). The 3D structures of compounds **2j** or 2‐methoxyestradiol were obtained, and their energy was minimised by molecular mechanics with Avogadro 1.90.0, while 3D structures of paclitaxel and colchicine were extracted from their original PDB entries. Polar hydrogens were added to ligands and proteins before docking, all using Flare ver 8.0.0 (Cresset Ltd.). The settings for the docking were as follows: for the PDB: 6I2I, the docking active site grid was created from (30.779, 44.713, 76.523) to (52.979, 66.913, 98.723) with the volume of the box equal to 10941 Å^3^. For the PDB: 7TQY, the docking active site grid was created from (140.407, 166.868, 185.516) to (163.587, 190.048, 208.696) with a volume of 12454.9 Å^3^ and for the PDB: 4O2B from (142.980, 145.890, 178.119) to (176.963, 201.486, 217.228) with volume of 73889 Å^3^.

For all the docking runs, the process configuration was set as ‘Accurate, Slow’, all H‐bond donor groups were allowed to rotate, and all hydrogens were set as flexible. Interactions of the candidate compound with the protein and the figures were generated in PyMOL ver. 2.0.4 (Schrödinger LLC).

### 4.4 Immunofluorescence Microscopy

Cells were cultivated in an 8‐well chamber µ‐Slide (IBIDI) for 24 h before treatment with compounds described in particular experiments. After the treatment, cells were washed with PBS and fixed with ice‐cold methanol:acetone (1:1) for 10 min. The slide was dried for 10 min and stored at –20°C. Subsequently, cells were rehydrated with PBS‐T (PBS with 1% Tween), blocked in 1% BSA in PBS‐T for 1 h and incubated with primary antibody against α‐tubulin (clone DM1A, Merck) for 4 h. Slides were then washed with PBS‐T and incubated with secondary antibody (goat anti‐mouse IgG conjugated with Alexa Fluor 488, ThermoFisher) for 1 h. Next, cells were washed twice with PBS‐T and once with PBS, then they were stained with DAPI (Merck) for 10 min, washed with PBS, covered with Mowiol (Merck) and analysed using a fluorescence microscope (Olympus IX51, Japan).

### 4.5 Tubulin Polymerisation Assay

Tubulin polymerisation assay was performed using the HTS assay based on cell‐free turbidimetric measurement using > 97% pure porcine tubulin (BK004P, Cytoskeleton). Compound **2j** or paclitaxel was added to tubulin dimers in general tubulin buffer (80 mM PIPES pH 6.9, 0.5 mM EGTA, 2.0 mM MgCl_2_, Cat. # BST01), and the polymerisation was initiated by the addition of GTP. Measurements of absorbance at 340 nm were performed at 37°C within a 60 min‐long kinetic assay (1 read per 60 s). Data obtained from two independent experiments were analysed using GraphPad Prism.

### 4.6 Tubulin Isolation and Dynamic Light Scattering (DLS)

Tubulin was purified from the soluble porcine brain homogenate by ammonium sulphate fractionation and ion exchange chromatography analogous to the published method [52]. The protein was stored in liquid nitrogen. Dynamic light scattering measurements were performed using Zetasizer Nano ZEN 3600 ZS (Malvern Instrument Ltd, Malvern, UK) equipped with a He–Ne laser. Solution of 1 mg/mL pure tubulin in 10 mM phosphate‐buffered saline (PBS) with 0.5 mM MgCl_2_, pH 7.0 was measured in the following setting (173° angle measurement, 25°C, triplicate). The hydrodynamic radius was calculated in the Zetasizer Software v. 7.13 (approximation fit to the sphere).

### 4.7 Microscale Thermophoresis (MST)

The MST method was performed to determine the binding affinity. Pure swine tubulin was fluorescently labelled using the BODIPY 630/650 NHS‐esther in a 1:1 dye/protein molar ratio. The labelled protein was diluted in 10 mM PBS with 0.5 mM MgCl_2_, pH 7.0. Measurements were performed in standard capillaries on a Monolith NT.115 instrument (NanoTemper Technologies) at 25°C with 5 s/20 s/5 s laser off/on/off times, with the excitation power set to 80%. Dose responses to the addition of the binders were analysed in duplicate and from binding curves, *K*_D_ values were calculated using Origin 8.0 (OriginLab).

### 4.8 Colony‐Formation Assay (CFA)

Low‐density cell suspension was seeded into six‐well plates and cultivated for 2 days, then the medium was replaced and fresh medium with tested compounds was added. The treated cells were further cultivated for 10 days. Then, the colonies were washed with PBS and fixed with 70% ethanol and stained with crystal violet (1% solution in 96% ethanol). Finally, colonies were washed and photographs were captured.

### 4.9 One‐Step Caspase 3/7 Activity Assay

HeLa cells were seeded in 96‐well tissue culture plates and allowed to adhere overnight. Increasing concentrations of tested compounds were added, and the cells were incubated for 24 h. After treatment, caspase assay buffer (30 mM MgCl_2_, 1.2 mM EGTA, 1.5% Nonidet P40, 0.3% CHAPS, 30% sucrose, 30 mM DTT, 3 mM PMSF) containing Ac‐DEVD‐AMC (Enzo Life Sciences) as a caspase 3/7 substrate was added to the wells. An inhibitor of caspase 3/7, Ac‐DEVD‐CHO (MedChemExpress), was used to analyse potential substrate autolysis in the samples. Culture plates were incubated for 4 h at 37°C and caspase 3/7 activity was measured using a TECAN microplate reader at 346 nm/442 nm (excitation/emission). The obtained data were normalised against an untreated control.

## Conflicts of Interest

The authors declare no conflicts of interest.

## Supplementary Data

^1^H and ^13^C NMR as well as UHPLC and HRMS spectra for all compounds and additional experimental results (cell cycle analyses, colony formation assay, immunofluorescence staining or further docking results) are included as a part of the Electronic Supplementary Information.

## Supporting information

ArchPharm_SupplMat_InChI.

Supplementary 120525.

Supporting Video 1_HeLa_cmpd 2j 10 uM 0‐24 h.

Supporting Video 2_HeLa CTRL 0‐24 h_1.

## Data Availability

The authors declare that the data supporting this article have been included as part of the Electronic Supplementary Information.

## References

[1] F. Pellegrini and D. R. Budman , “Review: Tubulin Function, Action of Antitubulin Drugs, and New Drug Development,” Cancer Investigation 23, no. 3 (2005): 264–273, 10.1081/CNV-200055970. 15948296

[2] M. A. Jordan and L. Wilson , “Microtubules as a Target for Anticancer Drugs,” Nature Reviews Cancer 4 (2004): 253–265, 10.1038/nrc1317. 15057285

[3] C. Dumontet and M. A. Jordan , “Microtubule‐Binding Agents: A Dynamic Field of Cancer Therapeutics,” Nature Reviews Drug Discovery 9 (2010): 790–803, 10.1038/nrd3253. 20885410 PMC3194401

[4] G. M. Alushin , G. C. Lander , E. H. Kellogg , R. Zhang , D. Baker , and E. Nogales , “High‐Resolution Microtubule Structures Reveal the Structural Transitions in αβ‐Tubulin Upon GTP Hydrolysis,” Cell 157, no. 5 (May 2014): 1117–1129, 10.1016/j.cell.2014.03.053. 24855948 PMC4054694

[5] H. Pérez‐Peña , A. C. Abel , M. Shevelev , A. E. Prota , S. Pieraccini , and D. Horvath , “Computational Approaches to the Rational Design of Tubulin‐Targeting Agents,” Biomolecules 13, no. 2 (February 2023): 285, 10.3390/biom13020285. 36830654 PMC9952983

[6] A. E. Prota , K. Bargsten , P. T. Northcote , et al., “Structural Basis of Microtubule Stabilization by Laulimalide and Peloruside A,” Angewandte Chemie International Edition 53, no. 6 (February 2014): 1621–1625, 10.1002/anie.201307749. 24470331

[7] M. O. Steinmetz and A. E. Prota , “Microtubule‐Targeting Agents: Strategies to Hijack the Cytoskeleton,” Trends in Cell Biology 28, no. 10 (October 2018): 776–792, 10.1016/j.tcb.2018.05.001. 29871823

[8] A. E. Prota , D. Lucena‐Agell , Y. Ma , et al., “Structural Insight Into the Stabilization of Microtubules by Taxanes,” eLife 12 (March 2023): e84791, 10.7554/eLife.84791. 36876916 PMC10049219

[9] E. H. Kellogg , N. M. A. Hejab , S. Howes , et al., “Insights Into the Distinct Mechanisms of Action of Taxane and Non‐Taxane Microtubule Stabilizers From Cryo‐EM Structures,” Journal of Molecular Biology 429, no. 5 (March 2017): 633–646, 10.1016/j.jmb.2017.01.001. 28104363 PMC5325780

[10] R. B. G. Ravelli , B. Gigant , P. A. Curmi , et al., “Insight Into Tubulin Regulation From a Complex With Colchicine and a Stathmin‐Like Domain,” Nature 428, no. 6979 (March 2004): 198–202, 10.1038/nature02393. 15014504

[11] X. Wang , B. Gigant , X. Zheng , and Q. Chen , “Microtubule‐Targeting Agents for Cancer Treatment: Seven Binding Sites and Three Strategies,” MedComm – Oncology 2 (2023): e46, 10.1002/mog2.46.

[12] F. Mahmud , S. Deng , H. Chen , D. D. Miller , and W. Li , “Orally Available Tubulin Inhibitor VERU‐111 Enhances Antitumor Efficacy in Paclitaxel‐Resistant Lung Cancer,” Cancer Letters 495 (December 2020): 76–88, 10.1016/j.canlet.2020.09.004. 32920198 PMC7669640

[13] R. Dreicer , F. Chu , D. J. Cahn , et al., “Phase 3 VERACITY Clinical Study of Sabizabulin in Men With Metastatic Castrate‐Resistant Prostate Cancer Who Have Progressed on an Androgen Receptor Targeting Agent,” Journal of Clinical Oncology 40, no. 6_suppl (February 2022): TPS217, 10.1200/JCO.2022.40.6_suppl.TPS217.

[14] T. Kalnins , V. Vitkovska , M. Kazak , et al., “Development of Potent Microtubule Targeting Agent by Structural Simplification of Natural Diazonamide,” Journal of Medicinal Chemistry 67, no. 11 (June 2024): 9227–9259, 10.1021/acs.jmedchem.4c00388. 38833507

[15] C. Wu , L. Zhang , Z. Zhou , et al., “Discovery and Mechanistic Insights Into Thieno[3,2‐d]pyrimidine and Heterocyclic Fused Pyrimidines Inhibitors Targeting Tubulin for Cancer Therapy,” European Journal of Medicinal Chemistry 276 (October 2024): 116649, 10.1016/j.ejmech.2024.116649. 38972078

[16] W. Zhao , R. Shen , H. M. Li , J. J. Zhong , and Y. J. Tang , “Podophyllotoxin Derivatives‐Tubulin Complex Reveals a Potential Binding Site of Tubulin Polymerization Inhibitors in α‐Tubulin Adjacent to Colchicine Site,” International Journal of Biological Macromolecules 276, no. Pt 1 (September 2024): 133678, 10.1016/j.ijbiomac.2024.133678. 38971286

[17] A. Sysak and B. Obmińska‐Mrukowicz , “Isoxazole Ring as a Useful Scaffold in a Search for New Therapeutic Agents,” European Journal of Medicinal Chemistry 137 (September 2017): 292–309, 10.1016/j.ejmech.2017.06.002. 28605676

[18] F. Hu and M. Szostak , “Recent Developments in the Synthesis and Reactivity of Isoxazoles: Metal Catalysis and Beyond,” Advanced Synthesis & Catalysis 357, no. 15 (2015): 2771–2777, 10.1002/adsc.201500319.

[19] T. Morita , S. Yugandar , S. Fuse , and H. Nakamura , “Recent Progresses in the Synthesis of Functionalized Isoxazoles,” Tetrahedron Letters 59, no. 13 (2018): 1159–1171, 10.1016/j.tetlet.2018.02.020.

[20] J. Zhu , J. Mo , H. Lin , Y. Chen , and H. Sun , “The Recent Progress of Isoxazole in Medicinal Chemistry,” Bioorganic & Medicinal Chemistry 26, no. 12 (July 2018): 3065–3075, 10.1016/j.bmc.2018.05.013. 29853341

[21] U. Malik and D. Pal , “Isoxazole Compounds: Unveiling the Synthetic Strategy, In‐Silico Sar & Toxicity Studies and Future Perspective as PARP Inhibitor in Cancer Therapy,” European Journal of Medicinal Chemistry 279 (November 2024): 116898, 10.1016/j.ejmech.2024.116898. 39353240

[22] G. C. Arya , K. Kaur , and V. Jaitak , “Isoxazole Derivatives as Anticancer Agent: A Review on Synthetic Strategies, Mechanism of Action and SAR Studies,” European Journal of Medicinal Chemistry 221 (October 2021): 113511, 10.1016/j.ejmech.2021.113511. 34000484

[23] A. S. Rudovich , M. Peřina , A. V. Krech , et al., “Synthesis and Biological Evaluation of New Isoxazolyl Steroids as Anti‐Prostate Cancer Agents,” International Journal of Molecular Sciences 23, no. 21 (November 2022): 13534, 10.3390/ijms232113534. 36362320 PMC9656436

[24] F. Kovács , D. I. Adamecz , F. I. Nagy , B. Papp , M. Kiricsi , and É. Frank , “Substitutional Diversity‐Oriented Synthesis and In Vitro Anticancer Activity of Framework‐Integrated Estradiol‐Benzisoxazole Chimeras,” Molecules 27, no. 21 (November 2022): 7456, 10.3390/molecules27217456. 36364293 PMC9654004

[25] M. Podhorecka , A. Macheta , S. Chocholska , et al., “Danazol Induces Apoptosis and Cytotoxicity of Leukemic Cells Alone and in Combination With Purine Nucleoside Analogs in Chronic Lymphocytic Leukemia,” Annals of Hematology 95, no. 3 (February 2016): 425–435, 10.1007/s00277-015-2579-5. 26692089 PMC4742499

[26] S. J. Deka , A. Roy , V. Ramakrishnan , D. Manna , and V. Trivedi , “Danazol Has Potential to Cause PKC Translocation, Cell Cycle Dysregulation, and Apoptosis in Breast Cancer Cells,” Chemical Biology & Drug Design 89, no. 6 (June 2017): 953–963, 10.1111/cbdd.12921. 27933735

[27] T. Capezzuoli , M. Rossi , F. La Torre , S. Vannuccini , and F. Petraglia , “Hormonal Drugs for the Treatment of Endometriosis,” Current Opinion in Pharmacology 67 (December 2022): 102311, 10.1016/j.coph.2022.102311. 36279764

[28] R. Minorics and I. Zupko , “Steroidal Anticancer Agents: An Overview of Estradiol‐Related Compounds,” Anti‐Cancer Agents in Medicinal Chemistry 18, no. 5 (2018): 652–666, 10.2174/1871520617666171114111721. 29141561

[29] R. J. D'Amato , C. M. Lin , E. Flynn , J. Folkman , and E. Hamel , “2‐Methoxyestradiol, an Endogenous Mammalian Metabolite, Inhibits Tubulin Polymerization by Interacting at the Colchicine Site,” Proceedings of the National Academy of Sciences 91, no. 9 (April 1994): 3964–3968, 10.1073/pnas.91.9.3964. PMC43703 8171020

[30] N. J. Lakhani , M. A. Sarkar , J. Venitz , and W. D. Figg , “2‐Methoxyestradiol, a Promising Anticancer Agent,” Pharmacotherapy: The Journal of Human Pharmacology and Drug Therapy 23, no. 2 (February 2003): 165–172, 10.1592/phco.23.2.165.32088. 12587805

[31] K. Kamath , T. Okouneva , G. Larson , D. Panda , L. Wilson , and M. A. Jordan , “2‐Methoxyestradiol Suppresses Microtubule Dynamics and Arrests Mitosis Without Depolymerizing Microtubules,” Molecular Cancer Therapeutics 5, no. 9 (September 2006): 2225–2233, 10.1158/1535-7163.MCT-06-0113. 16985056

[32] M. Sun , Y. Zhang , J. Qin , et al., “Synthesis and Biological Evaluation of New 2‐Methoxyestradiol Derivatives: Potent Inhibitors of Angiogenesis and Tubulin Polymerization,” Bioorganic Chemistry 113 (August 2021): 104988, 10.1016/j.bioorg.2021.104988. 34034135

[33] I. K. Njangiru , N. Bózsity‐Faragó , V. E. Resch , et al., “A Novel 2‐Methoxyestradiol Derivative: Disrupting Mitosis Inhibiting Cell Motility and Inducing Apoptosis in Hela Cells In Vitro,” Pharmaceutics 16, no. 5 (May 2024): 622, 10.3390/pharmaceutics16050622. 38794284 PMC11125453

[34] J. Huber , J. Wölfling , G. Schneider , et al., “Synthesis of Antiproliferative 13α‐D‐homoestrones via Lewis Acid‐Promoted One‐Pot Prins‐Ritter Reactions of D‐Secosteroidal Δ‐Alkenyl‐Aldehydes,” Steroids 102 (October 2015): 76–84, 10.1016/j.steroids.2015.07.004. 26210211

[35] L. Du , S. S. Yee , K. Ramachandran , and A. L. Risinger , “Elucidating Target Specificity of the Taccalonolide Covalent Microtubule Stabilizers Employing a Combinatorial Chemical Approach,” Nature Communications 11, no. 1 (January 2020): 654, 10.1038/s41467-019-14277-w. PMC6994698 32005831

[36] M. Peřina , M. A. Kiss , G. Mótyán , et al., “A‐Ring‐Fused Pyrazoles of Dihydrotestosterone Targeting Prostate Cancer Cells via the Downregulation of the Androgen Receptor,” European Journal of Medicinal Chemistry 249 (March 2023): 115086, 10.1016/j.ejmech.2023.115086. 36682291

[37] M. A. Kiss , M. Peřina , V. Bazgier , et al., “Synthesis of Dihydrotestosterone Derivatives Modified in the A‐Ring With (Hetero)Arylidene, Pyrazolo[1,5‐a]Pyrimidine and Triazolo[1,5‐a]Pyrimidine Moieties and Their Targeting of the Androgen Receptor in Prostate Cancer,” Journal of Steroid Biochemistry and Molecular Biology 211 (July 2021): 105904, 10.1016/j.jsbmb.2021.105904. 33933576

[38] G. Mótyán , M. K. Gopisetty , R. E. Kiss‐Faludy , et al., “Anti‐Cancer Activity of Novel Dihydrotestosterone‐Derived Ring A‐Condensed Pyrazoles on Androgen Non‐Responsive Prostate Cancer Cell Lines,” International Journal of Molecular Sciences 20, no. 9 (May 2019): 2170, 10.3390/ijms20092170. 31052484 PMC6539495

[39] É. Frank , Z. Mucsi , M. Szécsi , I. Zupkó , J. Wölfling , and G. Schneider , “Intramolecular Approach to Some New D‐Ring‐Fused Steroidal Isoxazolidines by 1,3‐Dipolar Cycloaddition: Synthesis, Theoretical and In Vitro Pharmacological Studies,” New Journal of Chemistry 34, no. 12 (2010): 2671–2681, 10.1039/C0NJ00150C.

[40] J. Hou , X. Zhang , W. Yu , and J. Chang , “I2‐Mediated Oxidative C‐O Bond Formation for the Synthesis of Isoxazoles,” Chinese Journal of Organic Chemistry 38, no. 12 (2018): 3236–3241, 10.6023/cjoc201806026.

[41] Y. Tsuda , M. Iimori , Y. Nakashima , et al., “Mitotic Slippage and the Subsequent Cell Fates After Inhibition of Aurora B During Tubulin‐Binding Agent–Induced Mitotic Arrest,” Scientific Reports 7 (2017): 16762, 10.1038/s41598-017-17002-z. 29196757 PMC5711930

[42] C. Hu , X. Zhu , G. H. Wang , et al., “Design, Synthesis and Anti‐Cancer Evaluation of Novel Podophyllotoxin Derivatives as Potent Tubulin‐Targeting Agents,” Medicinal Chemistry Research 27 (2018): 351–365, 10.1007/s00044-017-1992-9.

[43] S. Senese , Y. C. Lo , A. A. Gholkar , et al., “Microtubins: A Novel Class of Small Synthetic Microtubule Targeting Drugs That Inhibit Cancer Cell Proliferation,” Oncotarget 8, no. 61 (October 2017): 104007–104021, 10.18632/oncotarget.21945. 29262617 PMC5732783

[44] S. K. Kim , S. M. Cho , H. Kim , et al., “The Colchicine Derivative CT20126 Shows a Novel Microtubule‐Modulating Activity With Apoptosis,” Experimental & Molecular Medicine 45, no. 4 (April 2013): e19, 10.1038/emm.2013.38. 23598593 PMC3641401

[45] X. Zhang , H. Huang , Z. Xu , and R. Zhan , “2‐Methoxyestradiol Blocks Cell‐Cycle Progression at the G_2_/M Phase and Induces Apoptosis in Human Acute T Lymphoblastic Leukemia CEM Cells,” Acta Biochimica et Biophysica Sinica 42, no. 9 (September 2010): 615–622, 10.1093/abbs/gmq065. 20732853

[46] M. A. Kiss , M. Peřina , L. Bereczki , et al., “Dihydrotestosterone‐Based A‐Ring‐Fused Pyridines: Microwave‐Assisted Synthesis and Biological Evaluation in Prostate Cancer Cells Compared to Structurally Related Quinolines,” Journal of Steroid Biochemistry and Molecular Biology 231 (July 2023): 106315, 10.1016/j.jsbmb.2023.106315. 37086925

[47] D. T. Terrano , M. Upreti , and T. C. Chambers , “Cyclin‐Dependent Kinase 1‐Mediated Bcl‐xL/Bcl‐2 Phosphorylation Acts as a Functional Link Coupling Mitotic Arrest and Apoptosis,” Molecular and Cellular Biology 30, no. 3 (February 2010): 640–656, 10.1128/MCB.00882-09. 19917720 PMC2812246

[48] X. X. Guo , H. Kim , Y. Li , H. Yim , S. K. Lee , and Y. H. Jin , “Cdk2 Acts Upstream of Mitochondrial Permeability Transition During Paclitaxel‐Induced Apoptosis,” Protein & Cell 2, no. 7 (July 2011): 543–553, 10.1007/s13238-011-1071-9. 21822799 PMC4875236

[49] M. L. Shelanski , F. Gaskin , and C. R. Cantor , “Microtubule Assembly in the Absence of Added Nucleotides,” Proceedings of the National Academy of Sciences 70, no. 3 (March 1973): 765–768, 10.1073/pnas.70.3.765. PMC433354 4514990

[50] J. C. Lee and S. N. Timasheff , “In Vitro Reconstitution of Calf Brain Microtubules: Effects of Solution Variables,” Biochemistry 16, no. 8 (April 1977): 1754–1764, 10.1021/bi00627a037. 856260

[51] C. Wittmann , A. S. Sivchenko , F. Bacher , et al., “Inhibition of Microtubule Dynamics in Cancer Cells by Indole‐Modified Latonduine Derivatives and Their Metal Complexes,” Inorganic Chemistry 61, no. 3 (January 2022): 1456–1470, 10.1021/acs.inorgchem.1c03154. 34995063 PMC8790753

[52] V. Kryštof , D. Moravcová , M. Paprskářová , et al., “Synthesis and Biological Activity of 8‐Azapurine and Pyrazolo[4,3‐d]Pyrimidine Analogues of Myoseverin,” European Journal of Medicinal Chemistry 41, no. 12 (December 2006): 1405–1411, 10.1016/j.ejmech.2006.07.004. 16996651

[53] S. Sharma , C. Lagisetti , B. Poliks , R. M. Coates , D. G. I. Kingston , and S. Bane , “Dissecting Paclitaxel‐Microtubule Association: Quantitative Assessment of the 2’‐OH Group,” Biochemistry 52, no. 13 (April 2013): 2328–2336, 10.1021/bi400014t. 23473345 PMC3685414

[54] P. Sherline , J. T. Leung , and D. M. Kipnis , “Binding of Colchicine to Purified Microtubule Protein,” Journal of Biological Chemistry 250, no. 14 (July 1975): 5481–5486, 10.1016/S0021-9258(19)41207-6. 1141240

[55] R. Adib , J. M. Montgomery , J. Atherton , et al., “Mitotic Phosphorylation by NEK6 and NEK7 Reduces the Microtubule Affinity of EML4 to Promote Chromosome Congression,” Science Signaling 12, no. 594 (August 2019): eaaw2939, 10.1126/scisignal.aaw2939. 31409757

[56] B. Hunter , M. P. M. H. Benoit , A. B. Asenjo , et al., “Kinesin‐8‐Specific Loop‐2 Controls the Dual Activities of the Motor Domain According to Tubulin Protofilament Shape,” Nature Communications 13, no. 1 (July 2022): 4198, 10.1038/s41467-022-31794-3. PMC9300613 35859148

[57] A. E. Prota , F. Danel , F. Bachmann , et al., “The Novel Microtubule‐Destabilizing Drug BAL27862 Binds to the Colchicine Site of Tubulin With Distinct Effects on Microtubule Organization,” Journal of Molecular Biology 426, no. 8 (April 2014): 1848–1860, 10.1016/j.jmb.2014.02.005. 24530796

[58] A. Gjyrezi , F. Xie , O. Voznesensky , et al., “Taxane Resistance in Prostate Cancer Is Mediated by Decreased Drug‐Target Engagement,” Journal of Clinical Investigation 130, no. 6 (June 2020): 3287–3298, 10.1172/JCI132184. 32478682 PMC7259995

[59] B. Cevatemre , I. Bulut , B. Dedeoglu , et al., “Exploiting Epigenetic Targets to Overcome Taxane Resistance in Prostate Cancer,” Cell Death & Disease 15, no. 2 (February 2024): 132, 10.1038/s41419-024-06422-1. 38346967 PMC10861560

[60] J. Kroon , M. Puhr , J. T. Buijs , et al., “Glucocorticoid Receptor Antagonism Reverts Docetaxel Resistance in Human Prostate Cancer,” Endocrine‐Related Cancer 23, no. 1 (January 2016): 35–45, 10.1530/ERC-15-0343. 26483423 PMC4657186

[61] M. A. Kiss , “Potenciális Antiandrogének Előállítása a Dihidrotesztoszteron A‐Gyűrűjének Szerkezetmódosításaival [Production of Potential Antiandrogens by Structural Modifications of the A‐Ring of Dihydrotestosterone],” (Doctoral dissertation, University of Szeged, Hungary, ProQuest Dissertations & Theses, 2023): 31357234, 10.14232/phd.11867.

