# Supplementary material for: Isoxazole‐Based Compounds Targeting the Taxane‐Binding Site of Tubulin
Source: Arch Pharm (Weinheim). 2025 Jul 23;358(7):e70031. doi: 10.1002/ardp.70031 (PMC12287683; doi:10.1002/ardp.70031)
Supplement: Supplementary file 1 — ArchPharm_SupplMat_InChI. [file ARDP-358-e70031-s003.doc]

**Supplemental Material: Novel Compounds and Biological Screening Results**

**Isoxazole-based compounds targeting the taxane-binding site of tubulin**

Miroslav Peřina^1,*^, Márton A. Kiss^2,*^, Jakub Bělíček^1^, Veronika Vojáčková^1^, Denisa Veselá^1^, Renáta Minorics^3^, István Zupko^3^, Éva Frank^2,#^, Radek Jorda^1,#^

^1^*Department of Experimental Biology, Faculty of Science, Palacký University Olomouc, Šlechtitelů 27, 77900 Olomouc, Czech Republic*

^2^*Department of Molecular and Analytical Chemistry, University of Szeged, Dóm Tér 7-8, H-6720 Szeged, Hungary*

^3^*Institute of Pharmacodynamics and Biopharmacy, University of Szeged, Eötvös u. 6, H-6720 Szeged, Hungary*

*these authors contributed equally

^#^Corresponding authors: [frank@chem.u-szeged.hu](mailto:frank@chem.u-szeged.hu); Tel.: +36 62 544 275;

[radek.jorda@upol.cz](mailto:radek.jorda@upol.cz); Tel.: +420 585 634 854

| **Cmpd No.** | **InChI** | **Residual viability of HeLa upon 20 μM treatment for 72 h (mean ± SD, %)^a^** | **Dissoc. Constant (K_D_) for binding into the tubulin (mean ± SD, nM)^b^** |
| --- | --- | --- | --- |
| 1i | InChI=1S/C25H33NO2/c1-24-11-10-21-19(20(24)8-9-23(24)28)7-6-17-14-22(27)16(15-25(17,21)2)13-18-5-3-4-12-26-18/h3-5,12-13,17,19-21,23,28H,6-11,14-15H2,1-2H3/b16-13+/t17-,19-,20-,21-,23-,24-,25-/m0/s1 | 47.8 ± 0.8 | n.m. |
| 2a | InChI=1S/C26H33NO2/c1-25-13-12-21-18(20(25)10-11-23(25)28)9-8-17-14-22-19(15-26(17,21)2)24(29-27-22)16-6-4-3-5-7-16/h3-7,17-18,20-21,23,28H,8-15H2,1-2H3/t17-,18-,20-,21-,23-,25-,26-/m0/s1 | 92.6 ± 0.7 | n.m. |
| 2b | InChI=1S/C27H35NO2/c1-16-4-6-17(7-5-16)25-20-15-27(3)18(14-23(20)28-30-25)8-9-19-21-10-11-24(29)26(21,2)13-12-22(19)27/h4-7,18-19,21-22,24,29H,8-15H2,1-3H3/t18-,19-,21-,22-,24-,26-,27-/m0/s1 | 56.3 ± 2.4 | > 1000 |
| 2c | InChI=1S/C27H35NO3/c1-26-13-12-22-19(21(26)10-11-24(26)29)9-6-17-14-23-20(15-27(17,22)2)25(31-28-23)16-4-7-18(30-3)8-5-16/h4-5,7-8,17,19,21-22,24,29H,6,9-15H2,1-3H3/t17-,19-,21-,22-,24-,26-,27-/m0/s1 | 62.1 ± 6.1 | n.m. |
| 2d | InChI=1S/C26H32FNO2/c1-25-12-11-21-18(20(25)9-10-23(25)29)8-5-16-13-22-19(14-26(16,21)2)24(30-28-22)15-3-6-17(27)7-4-15/h3-4,6-7,16,18,20-21,23,29H,5,8-14H2,1-2H3/t16-,18-,20-,21-,23-,25-,26-/m0/s1 | 65.6 ± 0.7 | n.m. |
| 2e | InChI=1S/C26H32ClNO2/c1-25-12-11-21-18(20(25)9-10-23(25)29)8-5-16-13-22-19(14-26(16,21)2)24(30-28-22)15-3-6-17(27)7-4-15/h3-4,6-7,16,18,20-21,23,29H,5,8-14H2,1-2H3/t16-,18-,20-,21-,23-,25-,26-/m0/s1 | 67.0 ± 1.2 | n.m. |
| 2f | InChI=1S/C26H32BrNO2/c1-25-12-11-21-18(20(25)9-10-23(25)29)8-5-16-13-22-19(14-26(16,21)2)24(30-28-22)15-3-6-17(27)7-4-15/h3-4,6-7,16,18,20-21,23,29H,5,8-14H2,1-2H3/t16-,18-,20-,21-,23-,25-,26-/m0/s1 | 73.3 ± 0.4 | n.m. |
| 2g | InChI=1S/C24H31NO3/c1-23-10-9-18-15(17(23)7-8-21(23)26)6-5-14-12-19-16(13-24(14,18)2)22(28-25-19)20-4-3-11-27-20/h3-4,11,14-15,17-18,21,26H,5-10,12-13H2,1-2H3/t14-,15-,17-,18-,21-,23-,24-/m0/s1 | 55.7 ± 0.6 | 84.6 ± 3.1 |
| 2h | InChI=1S/C24H31NO2S/c1-23-10-9-18-15(17(23)7-8-21(23)26)6-5-14-12-19-16(13-24(14,18)2)22(27-25-19)20-4-3-11-28-20/h3-4,11,14-15,17-18,21,26H,5-10,12-13H2,1-2H3/t14-,15-,17-,18-,21-,23-,24-/m0/s1 | 47.0 ± 1.2 | 607 ± 95 |
| 2i | InChI=1S/C25H32N2O2/c1-24-11-10-19-16(18(24)8-9-22(24)28)7-6-15-13-21-17(14-25(15,19)2)23(29-27-21)20-5-3-4-12-26-20/h3-5,12,15-16,18-19,22,28H,6-11,13-14H2,1-2H3/t15-,16-,18-,19-,22-,24-,25-/m0/s1 | 63.4 ± 4.9 | 857 ± 96 |
| 2j | InChI=1S/C21H31NO2/c1-12-15-11-21(3)13(10-18(15)22-24-12)4-5-14-16-6-7-19(23)20(16,2)9-8-17(14)21/h13-14,16-17,19,23H,4-11H2,1-3H3/t13-,14-,16-,17-,19-,20-,21-/m0/s1 | 48.8 ± 0.6 | 14.7 ± 1.3 |

^a^ Cells were seeded and then treated in 96-well tissue culture plates for 72 h. Upon treatment, the resazurin solution (Sigma Aldrich) was added for 4 h, and then the fluorescence of resorufin was measured at 544 nm/590 nm (excitation/emission) using a Fluoroskan Ascent microplate reader (Labsystems). Percentual viability from replicates was calculated [1].

^b^ The MST method was performed to determine the binding affinity. Pure swine tubulin was fluorescently labelled using the BODIPY 630/650 NHS-esther in 1:1 dye/protein molar ratio. The labelled protein was diluted in the 10 mM PBS with 0.5 mM MgCl_2_, pH 7.0. Measurements were performed in standard capillaries on a Monolith NT.115 instrument (NanoTemper Technologies) at 25 °C with 5 sec/20 sec/5 sec laser off/on/off times, with the excitation power set to 80% [2]. Dose-responses to addition of the binders were analysed in duplicate and from binding curves, K_D_ values were calculated using Origin 8.0 (OriginLab).

n.m. – not measurable

[1] Kumar P, Nagarajan A, Uchil PD. Analysis of Cell Viability by the alamarBlue Assay. Cold Spring Harb Protoc. 2018 Jun 1;2018(6). doi: 10.1101/pdb.prot095489. PMID: 29858336.

[2] Jerabek-Willemsen M, Wienken CJ, Braun D, Baaske P, Duhr S. Molecular interaction studies using microscale thermophoresis. Assay Drug Dev Technol. 2011 Aug;9(4):342-53. doi: 10.1089/adt.2011.0380. PMID: 21812660; PMCID: PMC3148787.
