# Supplementary material for: Isoxazole‐Based Compounds Targeting the Taxane‐Binding Site of Tubulin
Source: Arch Pharm (Weinheim). 2025 Jul 23;358(7):e70031. doi: 10.1002/ardp.70031 (PMC12287683; doi:10.1002/ardp.70031)
Supplement: Supplementary file 2 — Supplementary 120525. [file ARDP-358-e70031-s002.docx]

**SUPPLEMENTARY INFORMATION**

for

**Isoxazole-based compounds targeting the taxane-binding site of tubulin**

Miroslav Peřina^1,*^, Márton A. Kiss^2,*^, Jakub Bělíček^1^, Veronika Vojáčková^1^, Denisa Veselá^1^, Renáta Minorics^3^, István Zupko^3^, Éva Frank^2,#^, Radek Jorda^1,#^

^1^*Department of Experimental Biology, Faculty of Science, Palacký University Olomouc, Šlechtitelů 27, 77900 Olomouc, Czech Republic*

^2^*Department of Molecular and Analytical Chemistry, University of Szeged, Dóm Tér 7-8, H-6720 Szeged, Hungary*

^3^*Institute of Pharmacodynamics and Biopharmacy, University of Szeged, Eötvös u. 6, H-6720 Szeged, Hungary*

*these authors contributed equally

#Corresponding authors: [frank@chem.u-szeged.hu](mailto:frank@chem.u-szeged.hu); Tel.: +36 62 544 275;

[radek.jorda@upol.cz](mailto:radek.jorda@upol.cz); Tel.: +420 585 634 854

**Video caption**

The LionheartFX (Biotek) live cell imaging time-lapse capture of control (**Video 1**) or 10 uM **2j** (**Video 2**)-treated cells in the brightfield mode with phase contrast with 1 photo per hour within the 24 h treatment (treatments were captured in duplicate and representative replicate was shown).

**Supplementary Table 1.** Residual viability of the selected cell lines upon 72 h treatment with 20 μM compounds.

| Residual viability (%, norm)^a^ | | | | | | | | | | | | |
| --- | --- | --- | --- | --- | --- | --- | --- | --- | --- | --- | --- | --- |
|  | Hela | | 22Rv1 | | C4-2 | | LAPC-4 | | DU145 | | BJ | |
|  | Mean | SD | Mean | SD | Mean | SD | Mean | SD | Mean | SD | Mean | SD |
| **1i** | 47.8 | 0.8 | 42.7 | 14.4 | 40.1 | 4.5 | 86.4 | 5.9 | 45.0 | 13.7 | 4.2 | 0.1 |
| **2a** | 92.6 | 0.7 | 89.9 | 5.1 | 89.0 | 14.0 | 85.7 | 4.0 | 101.7 | 3.0 | 119.7 | 0.7 |
| **2b** | 56.3 | 2.4 | 80.4 | 6.0 | 81.0 | 18.3 | 88.2 | 1.0 | 108.0 | 4.2 | 122.0 | 3.3 |
| **2c** | 62.1 | 6.1 | 92.7 | 6.6 | 86.0 | 17.0 | 91.7 | 2.9 | 110.0 | 1.5 | 135.8 | 2.3 |
| **2d** | 65.6 | 0.7 | 102.1 | 13.7 | 90.0 | 13.8 | 84.7 | 5.8 | 113.4 | 8.8 | 144.4 | 7.2 |
| **2e** | 67.0 | 1.2 | 82.0 | 6.4 | 85.0 | 18.4 | 97.8 | 2.0 | 108.4 | 2.4 | 99.5 | 10.5 |
| **2f** | 73.3 | 0.4 | 77.2 | 12.6 | 84.7 | 21.5 | 105.4 | 2.6 | 107.4 | 0.2 | 65.7 | 0.2 |
| **2g** | 55.7 | 0.6 | 47.1 | 6.1 | 52.6 | 5.3 | 70.8 | 6.1 | 106.2 | 9.7 | 119.0 | 8.6 |
| **2h** | 47.0 | 1.2 | 48.6 | 3.4 | 50.0 | 22.6 | 72.9 | 8.2 | 110.4 | 2.7 | 113.5 | 12.1 |
| **2i** | 63.4 | 4.9 | 76.5 | 6.6 | 80.4 | 4.4 | 70.9 | 4.3 | 108.8 | 1.1 | 125.1 | 7.2 |
| **2j** | 48.8 | 0.6 | 41.3 | 2.9 | 47.3 | 8.8 | 63.5 | 6.6 | 65.4 | 7.9 | 123.4 | 1.4 |

^a^Cytotoxic effect of compounds was evaluated by resazurin-based viability assay with a single dose of 20 µM compounds upon 72h treatment. Measured in duplicate and repeated at least twice, mean and standard deviation (SD) is shown.

**Supplementary Table 2.** Viability of selected cell lines upon 72 h treatment with most potent compounds and standards.

| Cytotoxic activity (GI_50_) | | | | | | | | | | |
| --- | --- | --- | --- | --- | --- | --- | --- | --- | --- | --- |
|  | HeLa | | C4-2 | | BJ | | DU145 | | DU145-DR | |
|  | Mean | SD | Mean | SD | Mean | SD | Mean | SD | Mean | SD |
| **2g** | 4.64 | 0.05 | 8.58 | 1.09 | 48.80 | 1.41 | > 50 |  | 6.76 | 0.90 |
| **2h** | 6.34 | 0.81 | 5.64 | 0.35 | > 50 |  | 12.59 | 0.73 | 8.78 | 0.38 |
| **2j** | 3.23 | 0.52 | 6.07 | 0.25 | > 50 |  | 5.96 | 0.13 | 3.35 | 0.08 |
| danazol | n.a. | | 9.86 | 1.69 | 27.00 | 2.39 | 25.11 | 4.02 | 9.09 | 0.49 |
| paclitaxel | 0.0134 | 0.0054 | 0.0024 | 0.0002 | 0.141 | 0.083 | 0.0030 | 0.0002 | 0.301 | 0.009 |
| docetaxel | n.a. | | n.a. | | n.a. | | 0.0001 | 0.00004 | 0.202 | 0.019 |

^a^Measured by crystal violet-based viability assay upon 72 h treatment. Mean of GI_50_ calculated from at least two independent experiments is shown; n.a., not analysed.


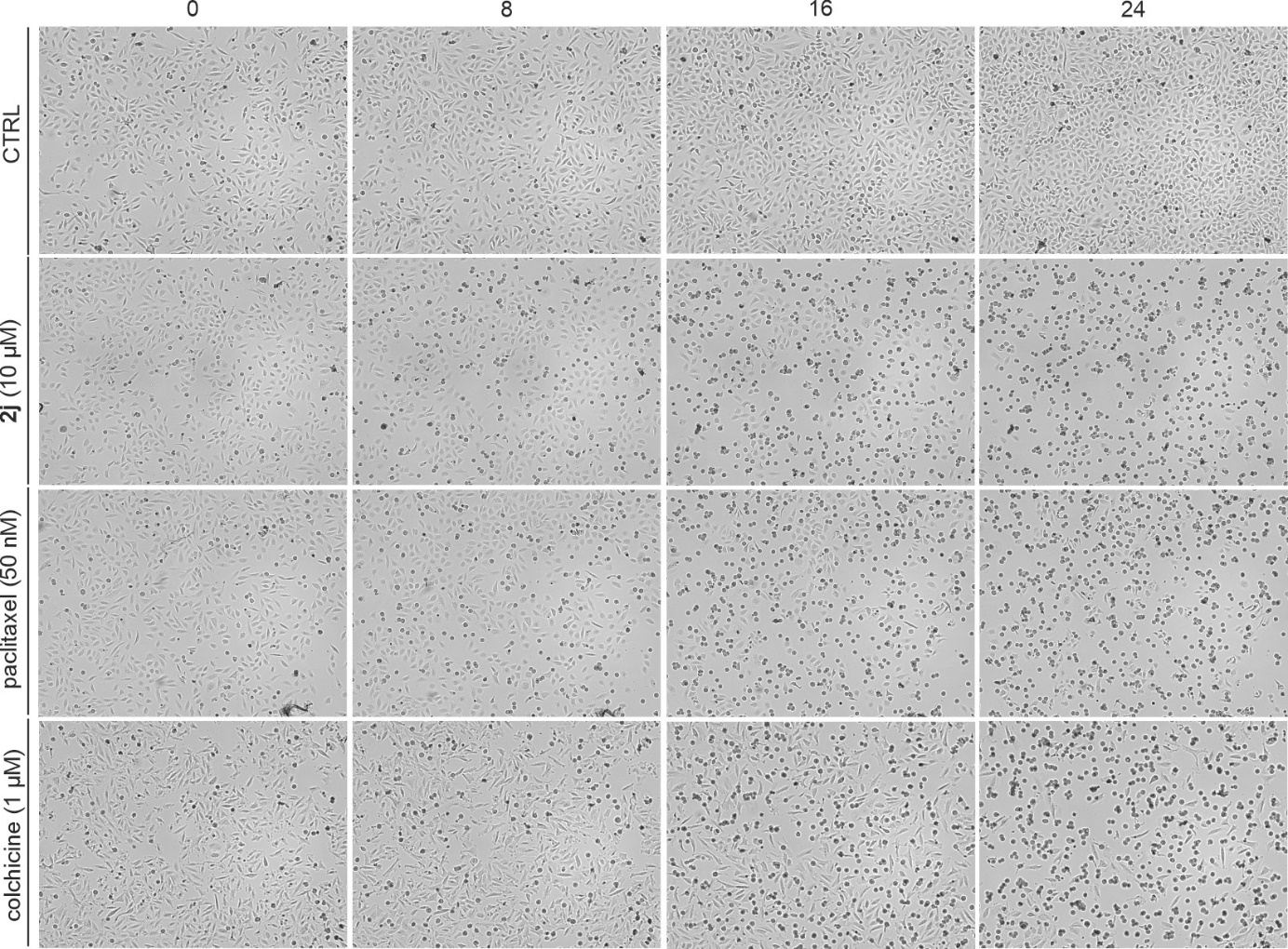


**Supplementary Figure 1.** Brightfield live cell imaging microscopy of the HeLa cells shape after the treatment with compound **2j** (10 μM) or paclitaxel (50 nM) or colchicine (1 μM) shown in particular time points. Magnification 40x.


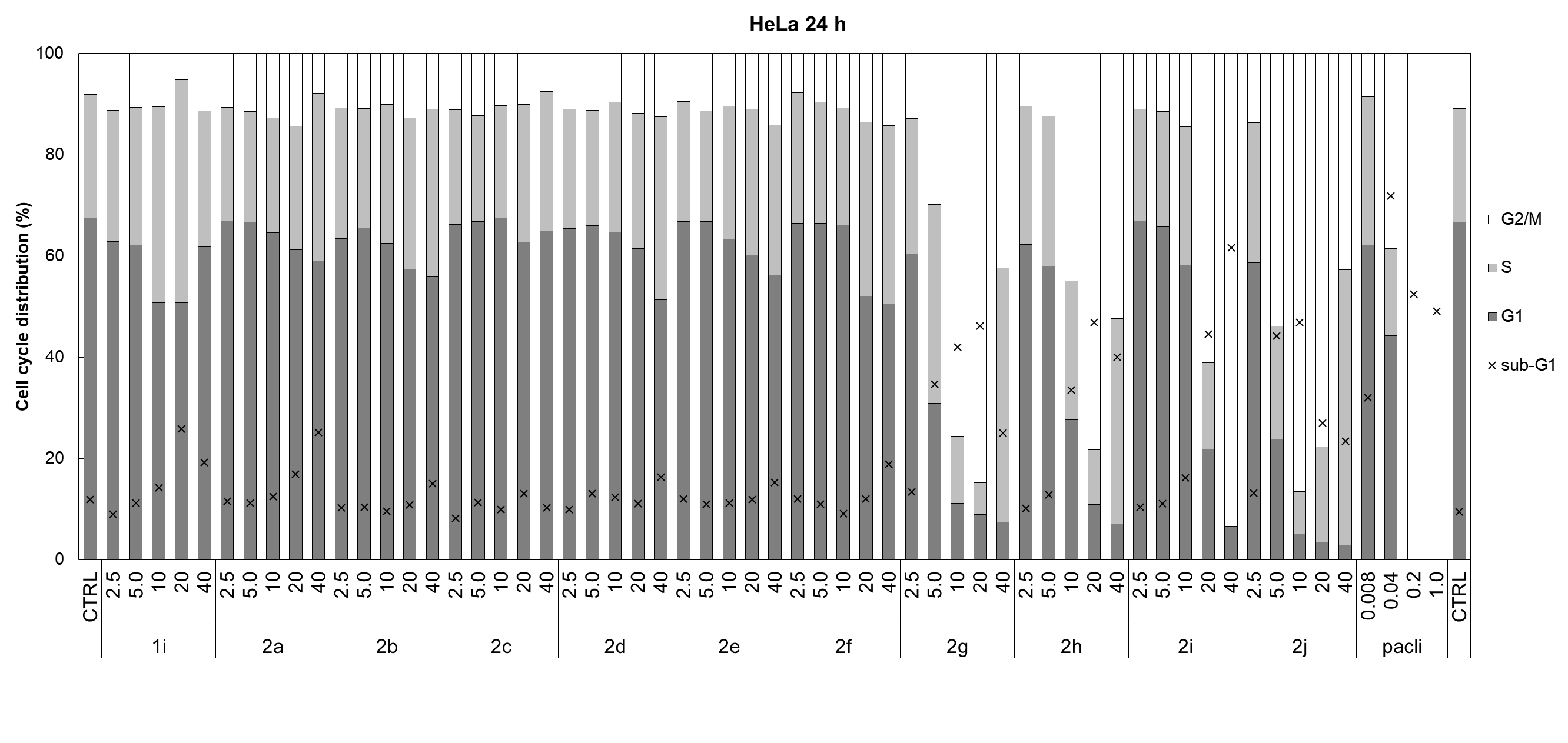


**Supplementary Figure 2.** Cell cycle distribution of HeLa cells upon 24 h treatment with the entire set of compounds and paclitaxel (pacli) in selected concentrations (μM).


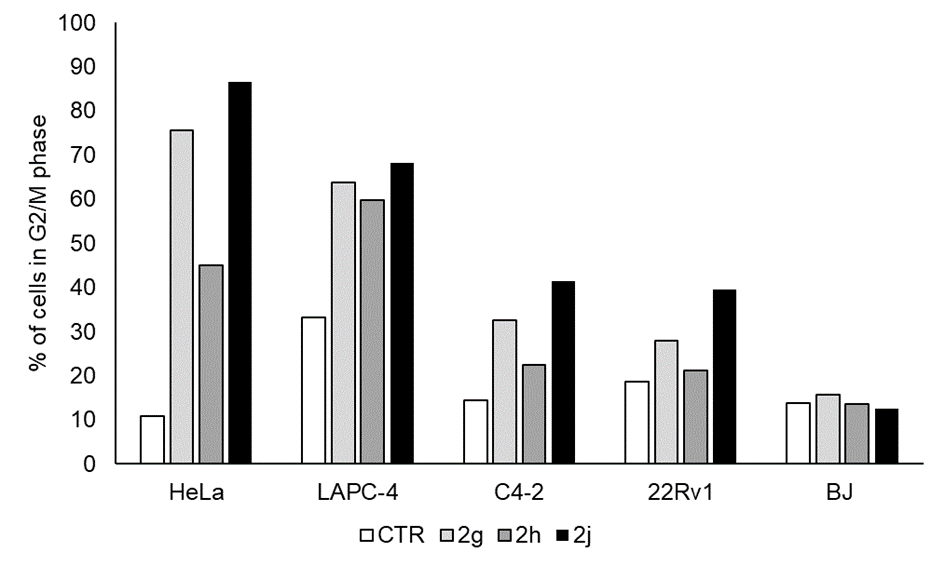


**Supplementary Figure 3.** Cell cycle distribution of HeLa, LAPC-4, C4-2 and 22Rv1 cells upon 24 h treatment with the most potent compounds in 10 µM concentrations.


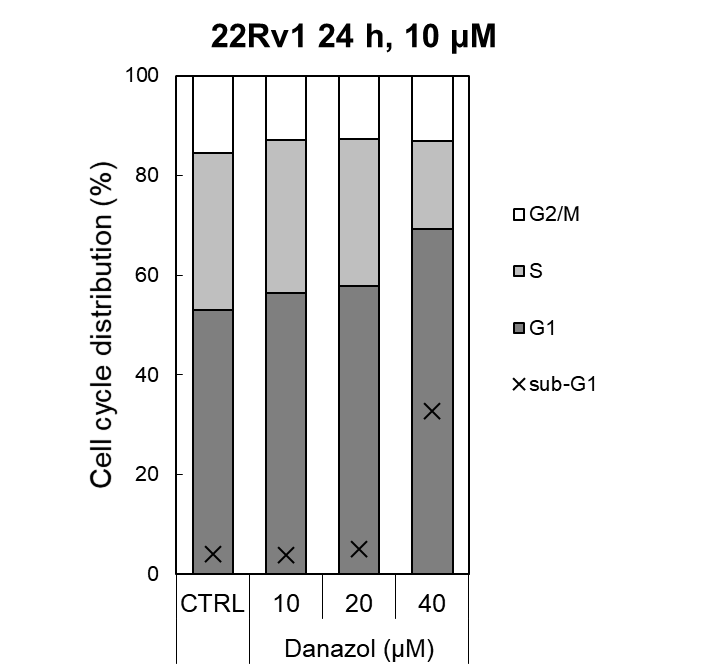

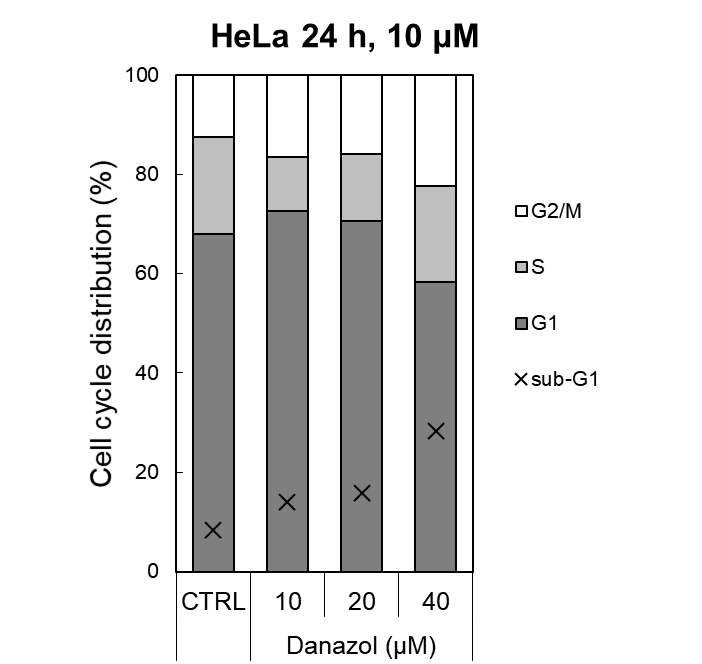


**Supplementary Figure 4.** Cell cycle distribution of 22Rv1(left) and HeLa (right) cells upon 24 h treatment with danazol.

**
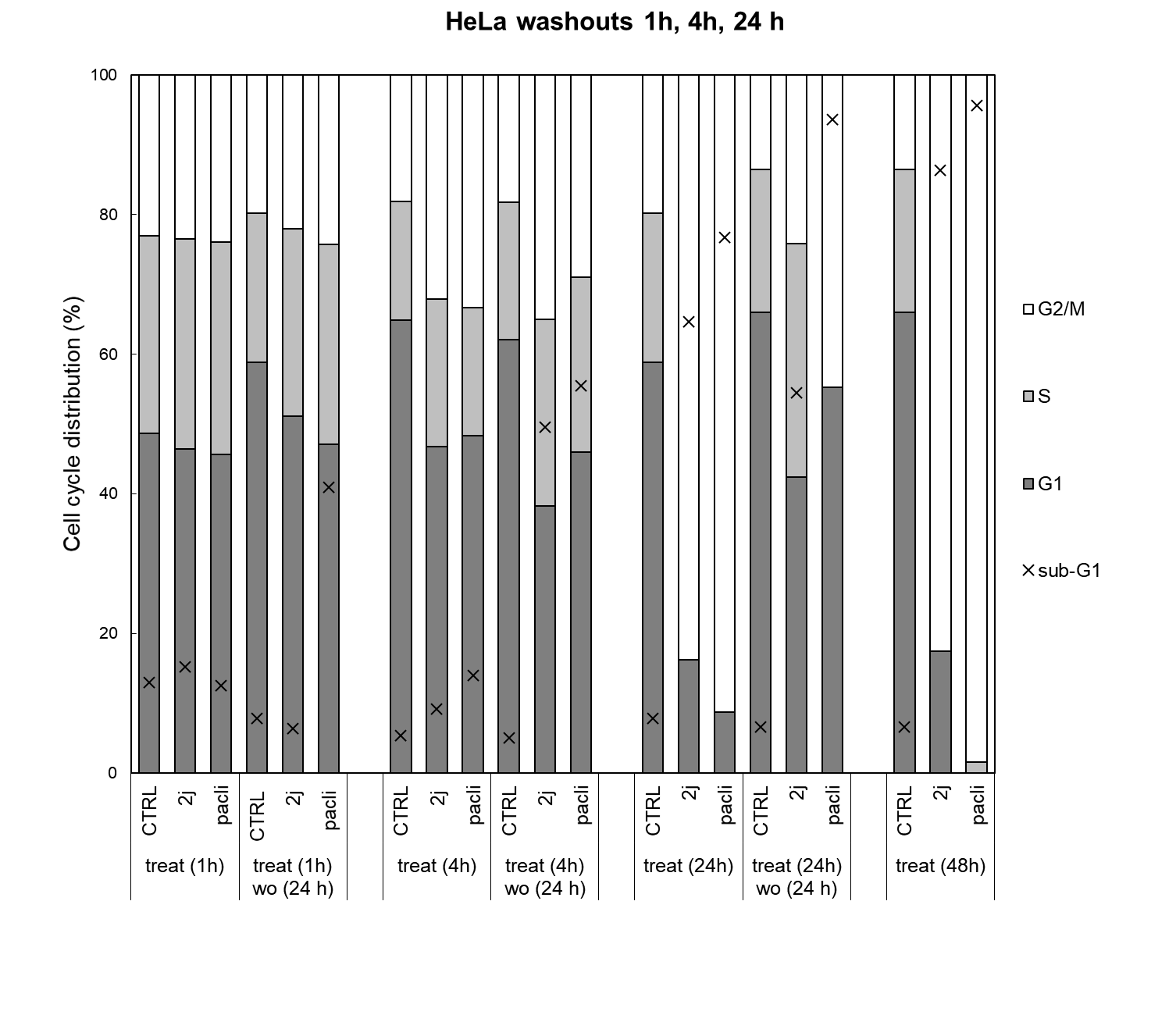
**

**Supplementary Figure 5.** Cell cycle distribution of HeLa cells upon different time treatments with lead compound **2j** (10 μM) or paclitaxel (50 nM) and following washouts. Treat, treatment; wo, washout.


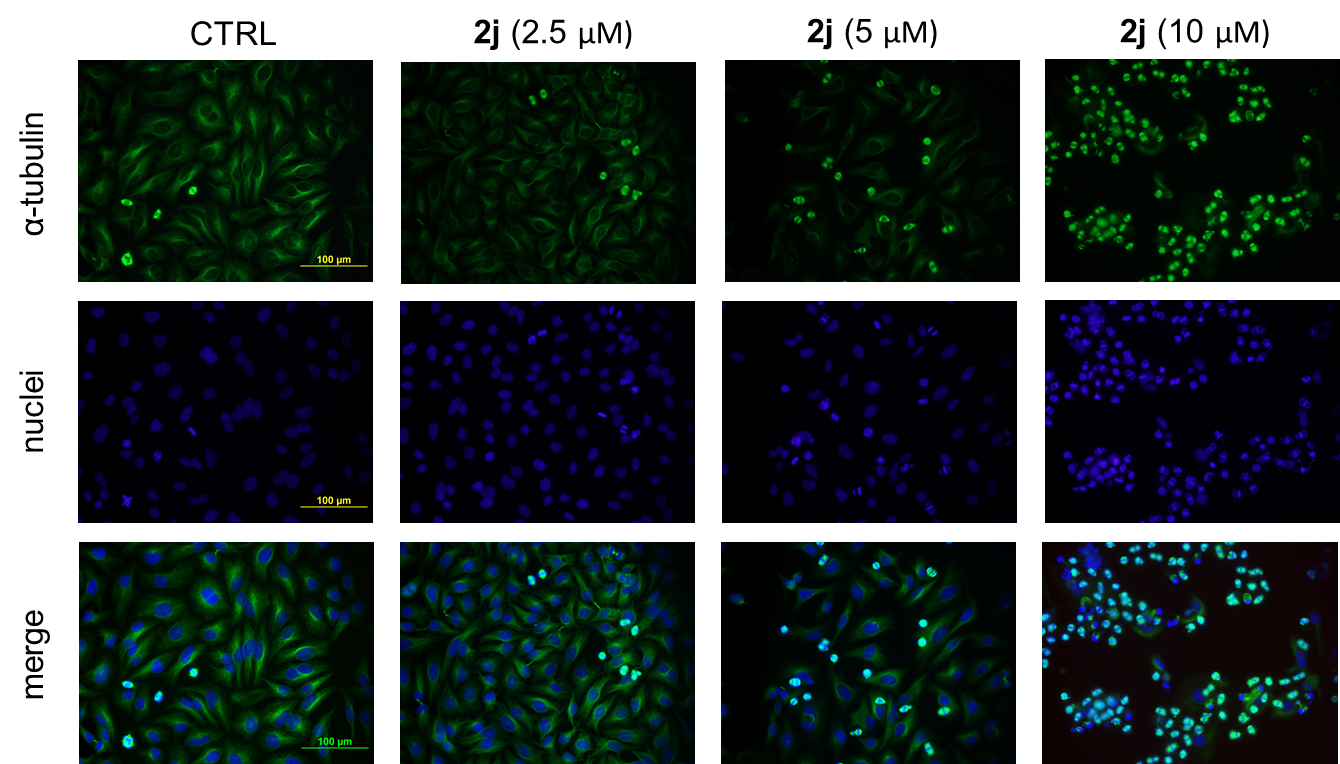


**Supplementary Figure 6.** Immunofluorescence images of HeLa upon 24 h treatment with different doses of **2j.** Upon the treatment, cells were fixed and α-tubulin was stained by Alexa 488-conjugated antibody and nuclear DNA by DAPI. The scale bar represents 100 μm, magnification 400x.


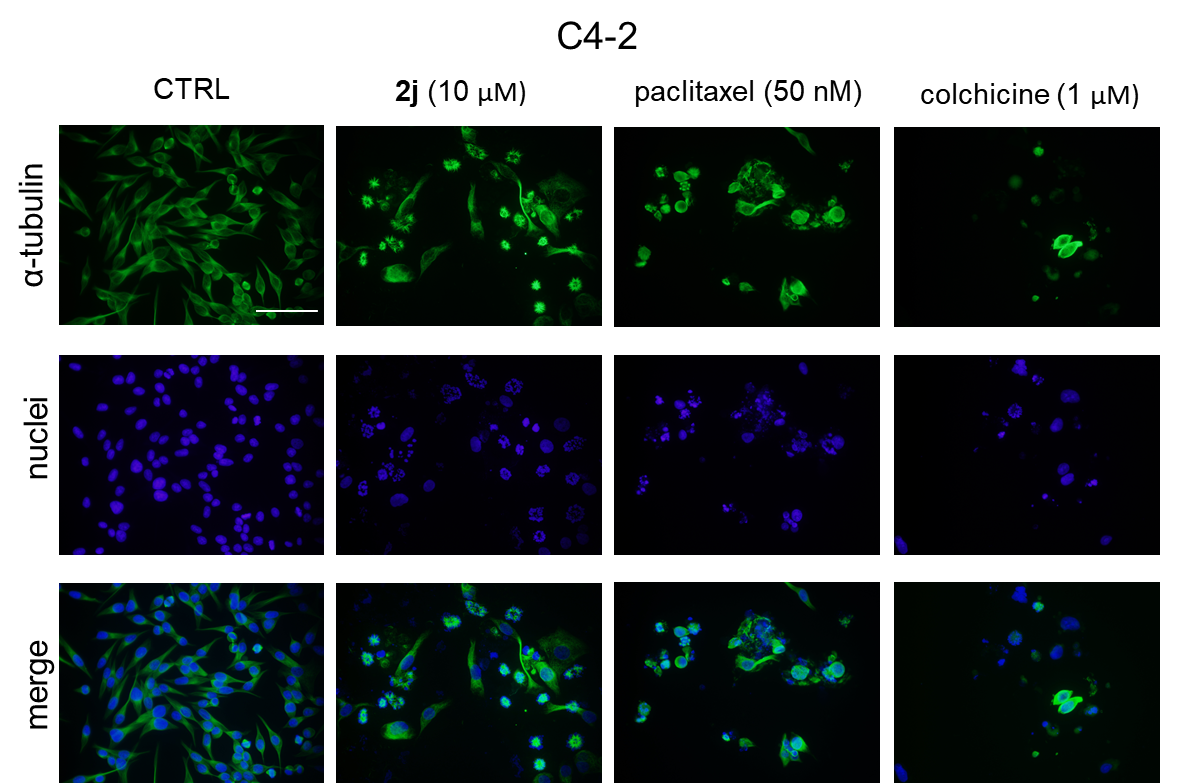


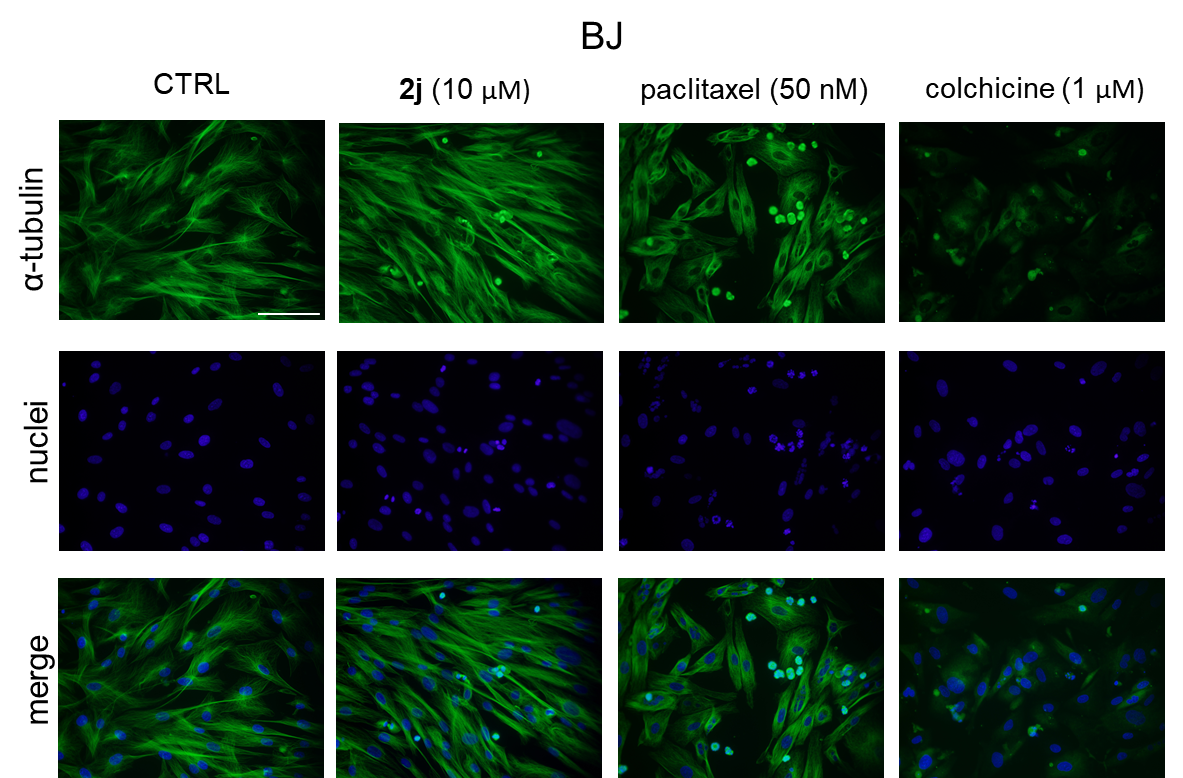


**Supplementary Figure 7.** Immunofluorescence images of C4-2 and BJ upon 24 h treatment with **2j** (10 μM) or paclitaxel (50 nM) and colchicine (1 μM). Upon the treatment, cells were fixed and α-tubulin was stained by Alexa 488-conjugated antibody and nuclear DNA by DAPI. The scale bar represents 100 μm, magnification 400x.


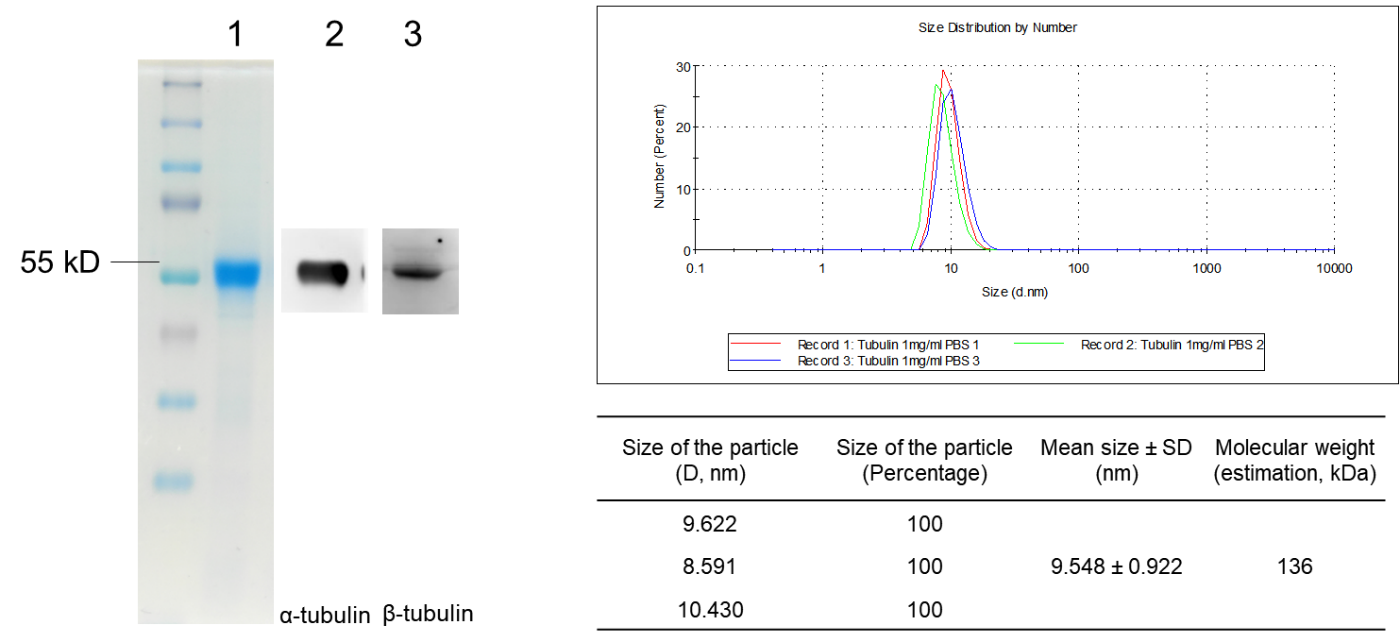


**Supplementary Figure 8. (A)** Purity of isolated tubulin dimer assessed by SDS-PAGE and identification of both protein monomers by western blot. **(B)** DLS measurements (*n* = 3) of isolated tubulin in 1 mg/ml with calculated mean size and estimation of molecular weight.


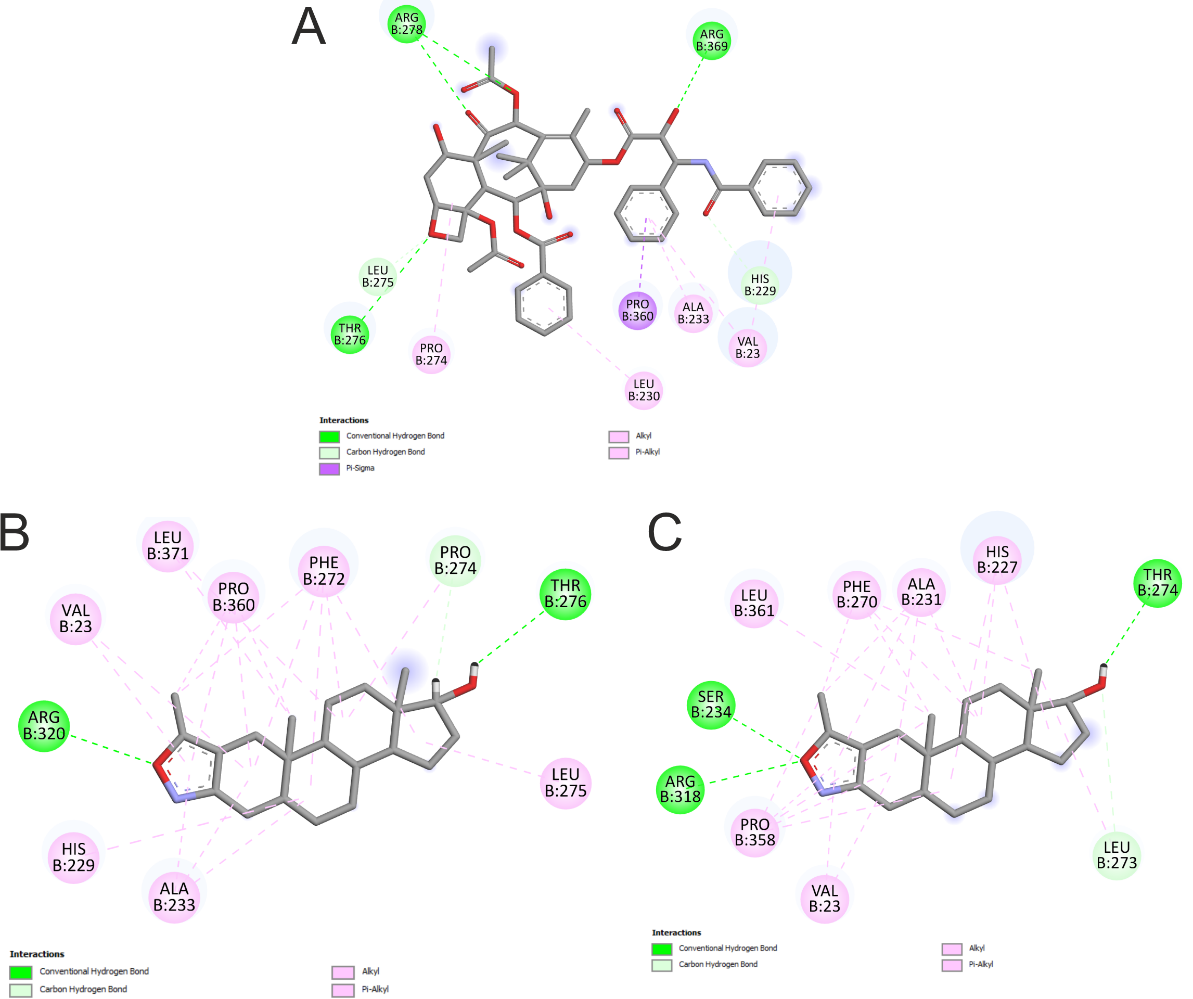


**Supplementary Figure 9. (A)** Paclitaxel 2D-interaction map in the cryo-EM structure of human HeLa tubulin (PDB: 6I2I). **(B)** Best pose **2j** 2D**-**interaction map in the taxane binding site of the cryo-EM structure of human HeLa tubulin (PDB: 6I2I). (**C**) Best pose **2j** 2D interaction map in the taxane binding site of the porcine tubulin (PDB: 7TQY)

**
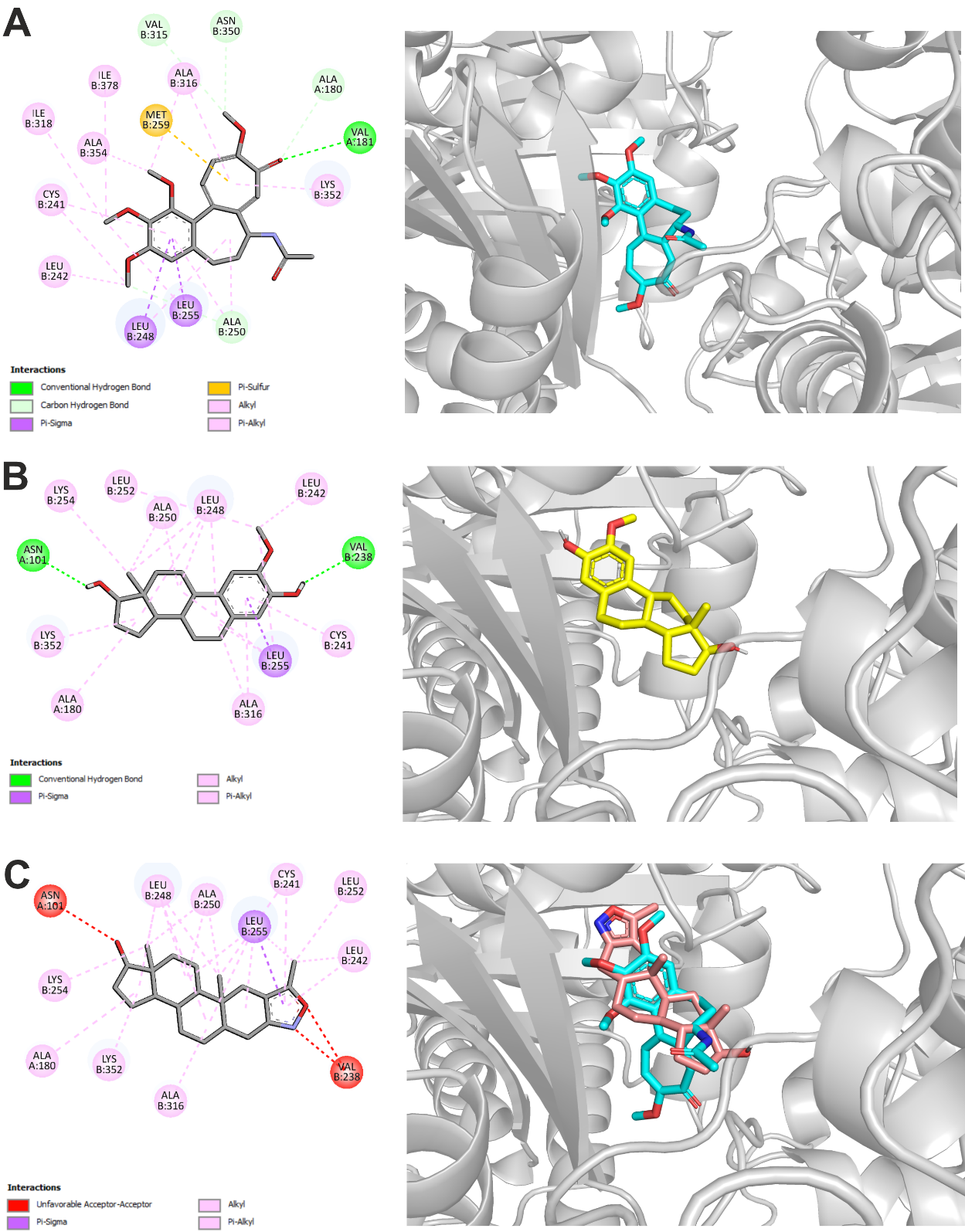
**

**Supplementary Figure 10. (A)** Interaction map and binding mode of colchicine (cyan) in the colchicine binding site from the co-crystal structure with tubulin (PDB: 4O2B). **(B)** Interaction map and binding mode of 2-methoxyestradiol (yellow) modelled into the colchicine binding site of tubulin (PDB: 4O2B). (**C**) Interaction map and binding mode of **2j** (pink) aligned to colchicine (cyan) into the colchicine binding site of tubulin (PDB: 4O2B).

*
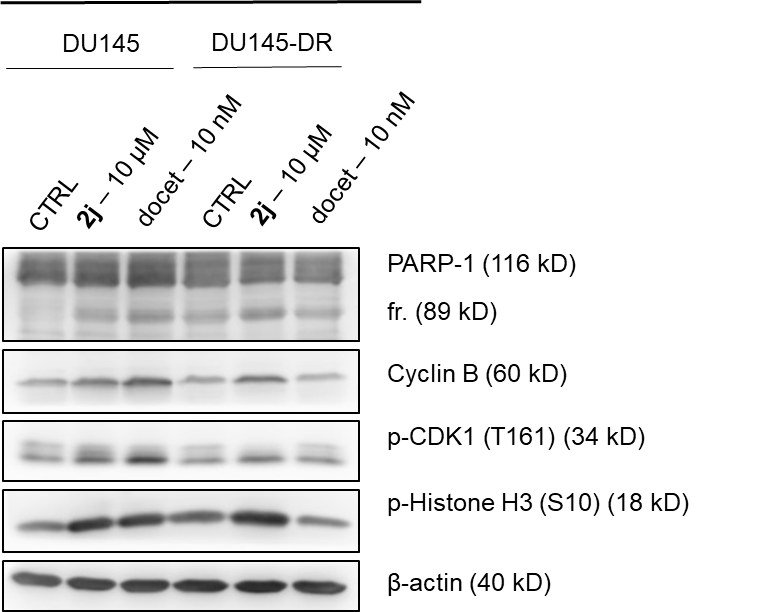
*

**Supplementary Figure 11.** Markers of the mitotic block in DU145 and DU145-DR cells upon the treatment with **2j** or docetaxel**.**


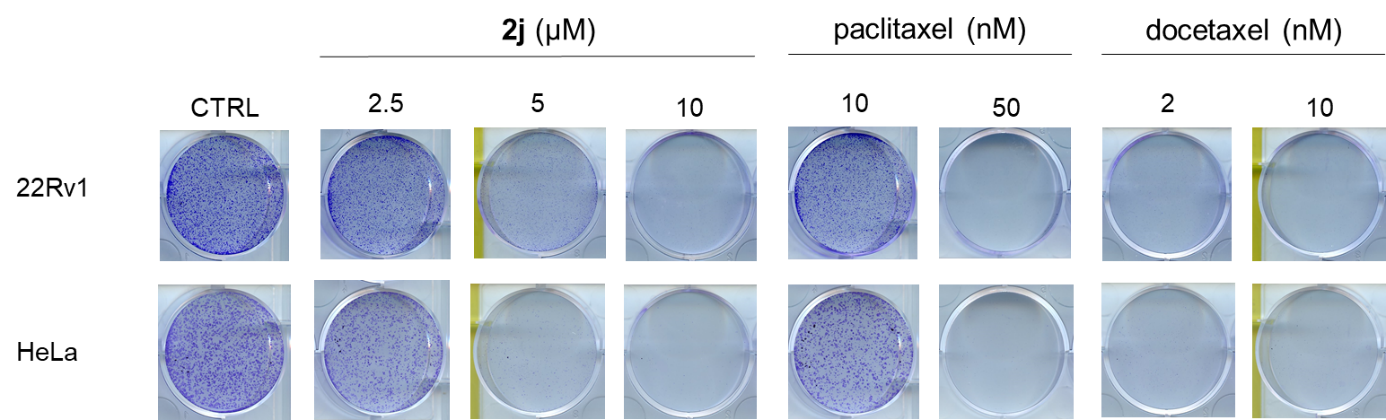


**Supplementary Figure 12.** Colony formation assay of 22Rv1 and HeLa cells upon 10-day treatment with candidate compound **2j**, paclitaxel and docetaxel.

**^1^H and ^13^C NMR spectra of the synthesised compounds**
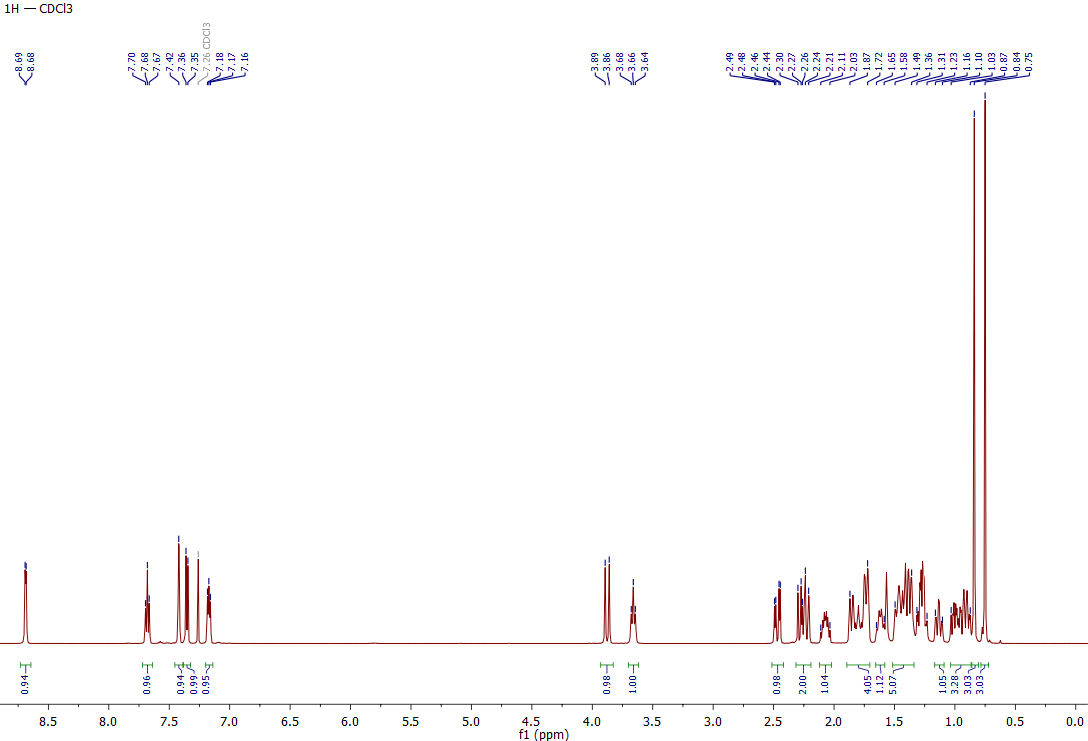

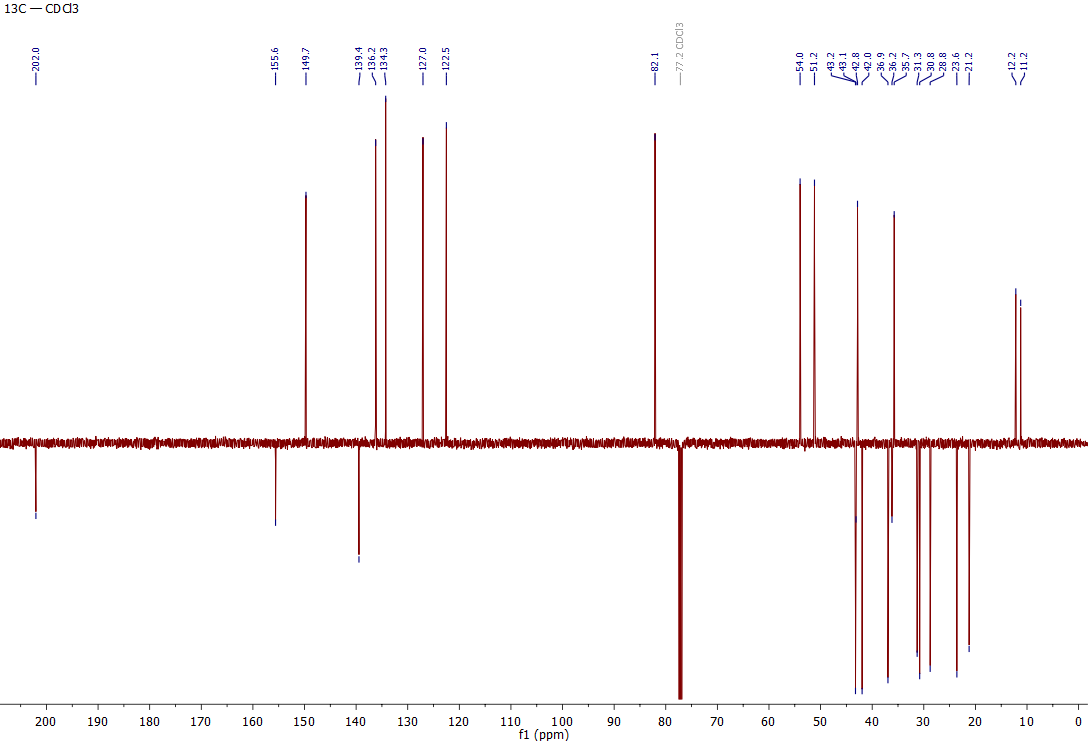


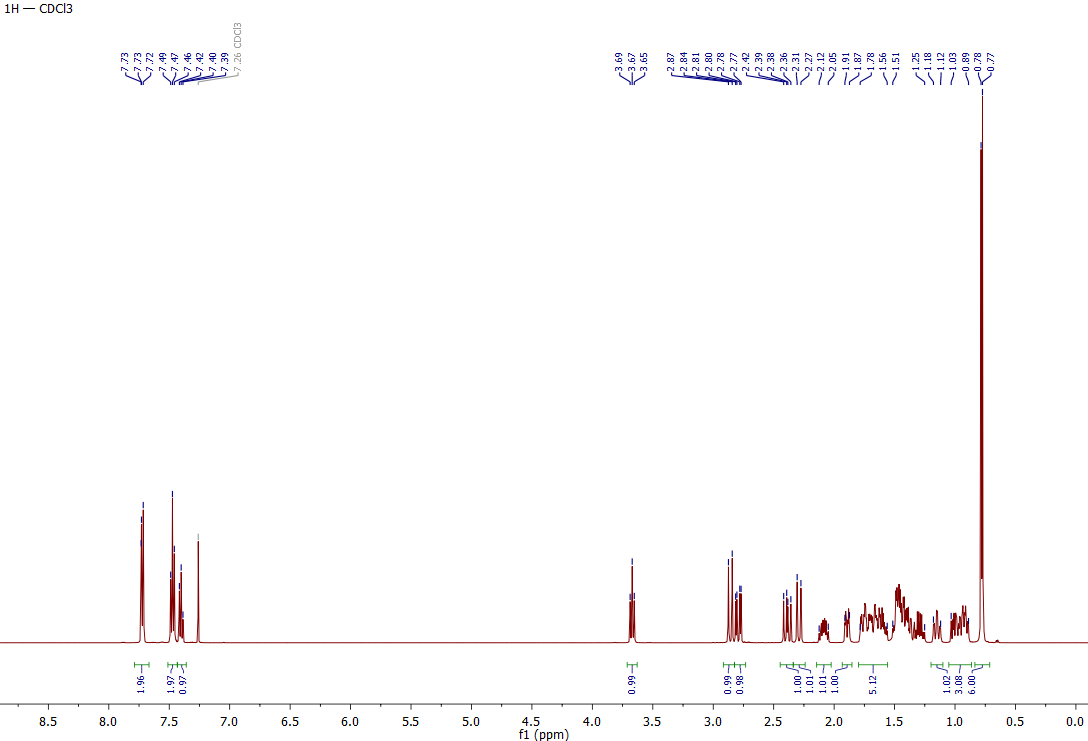

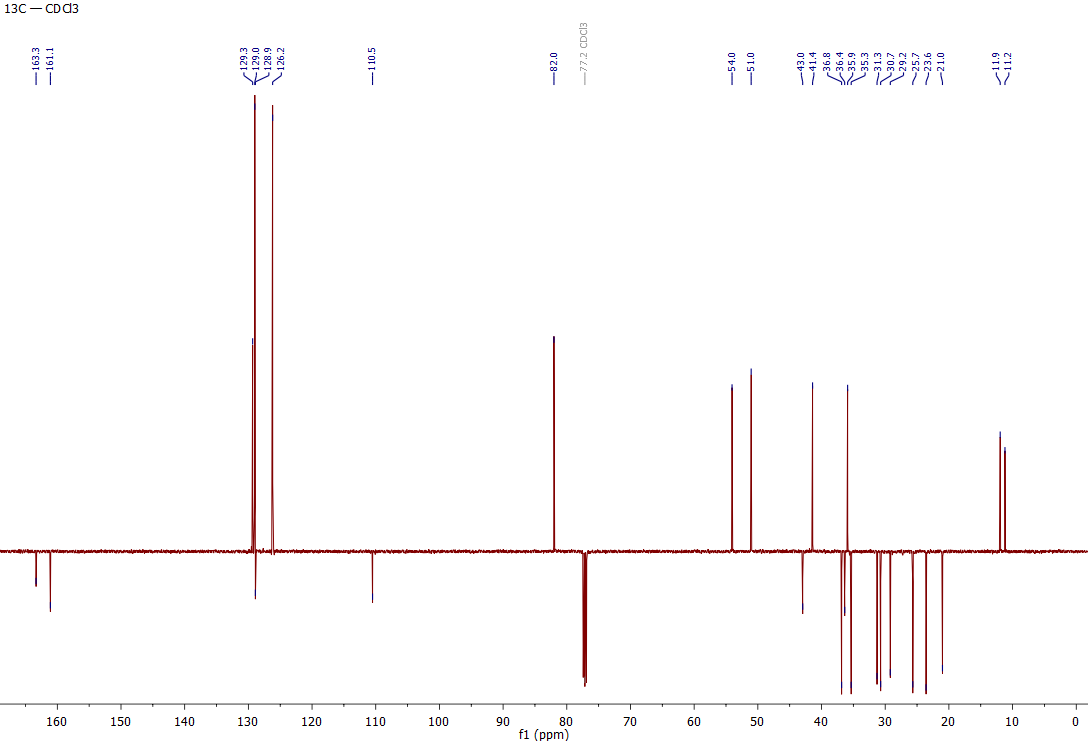


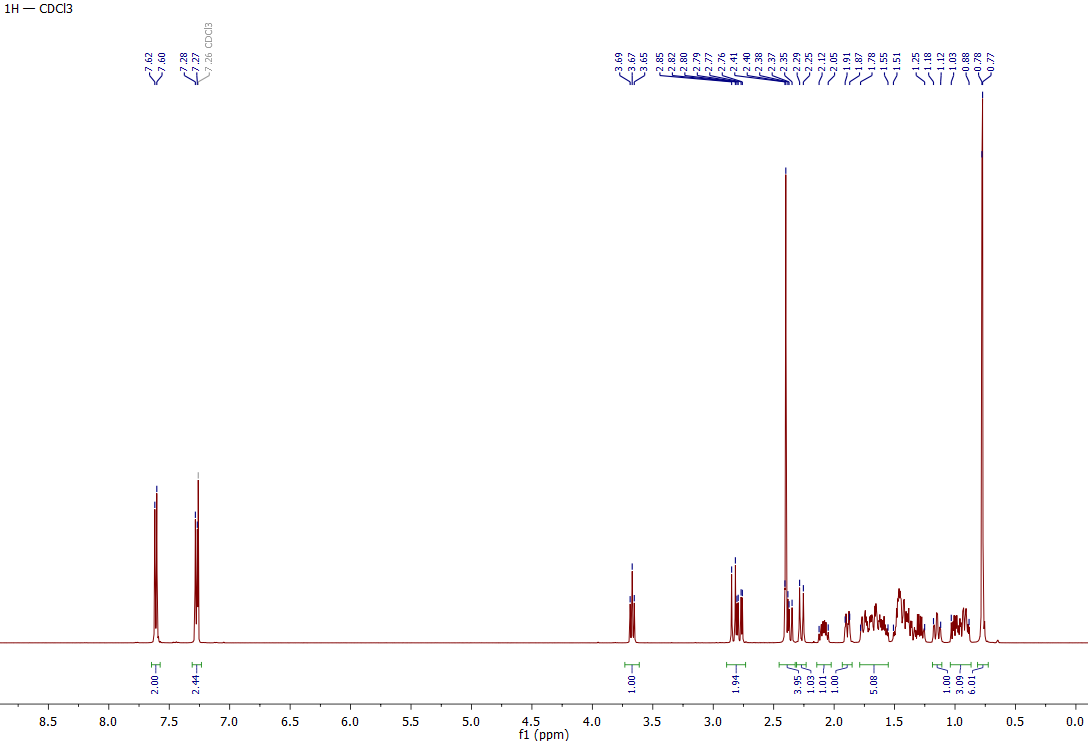

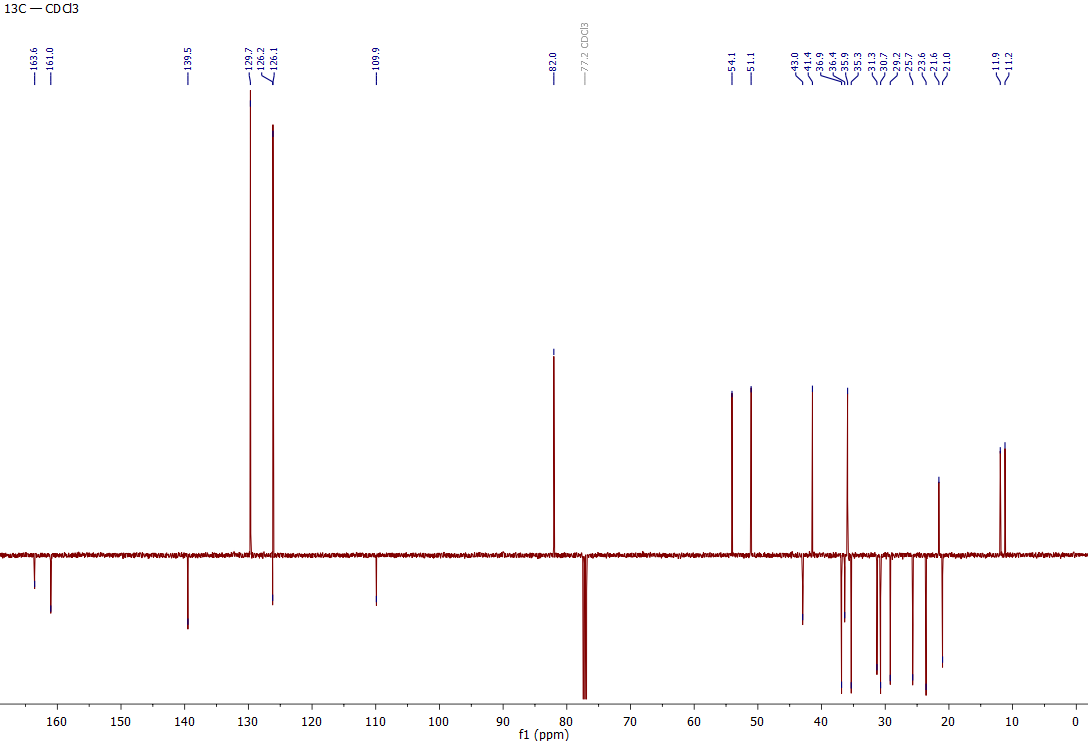

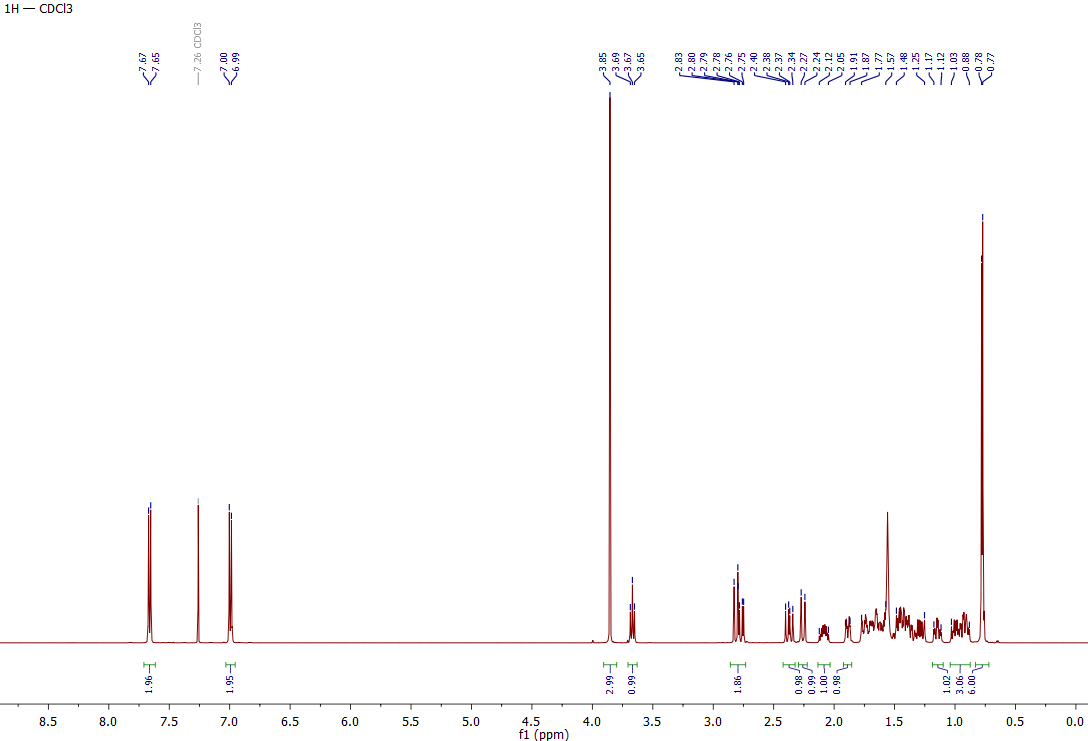

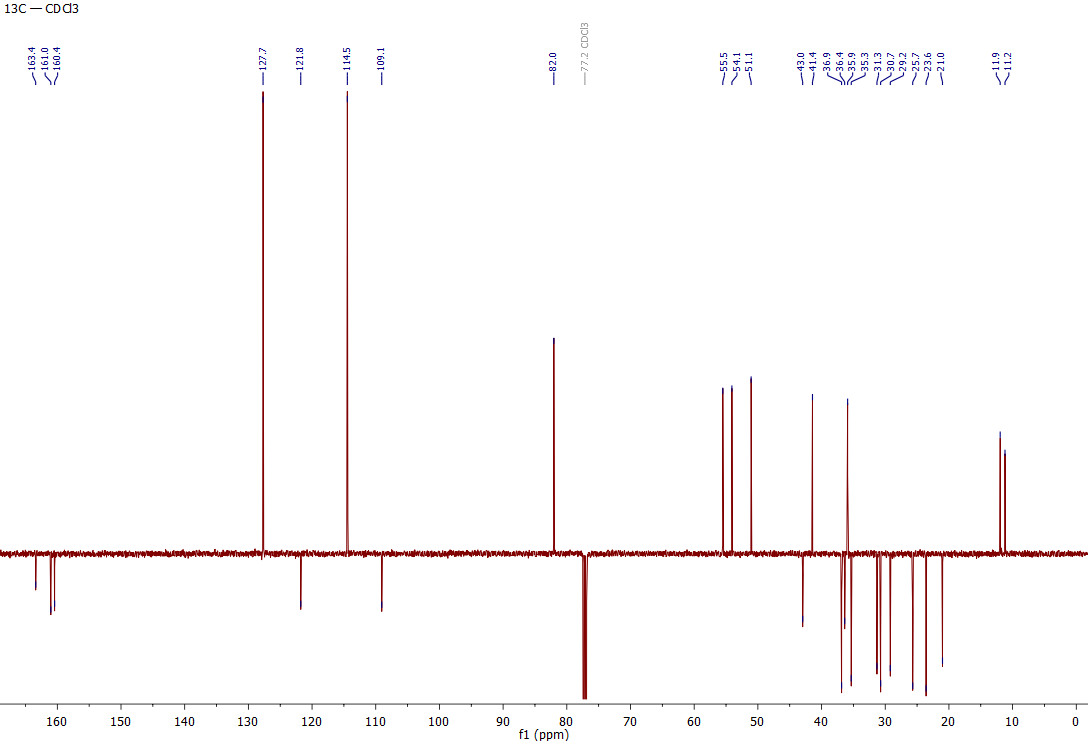


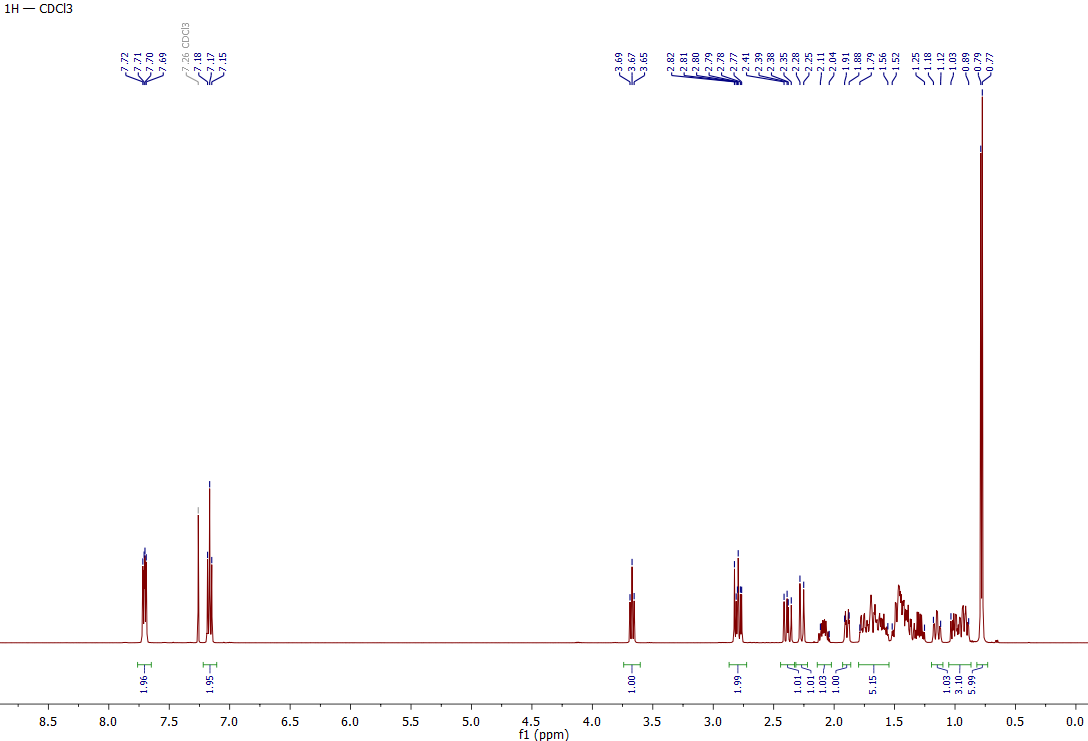

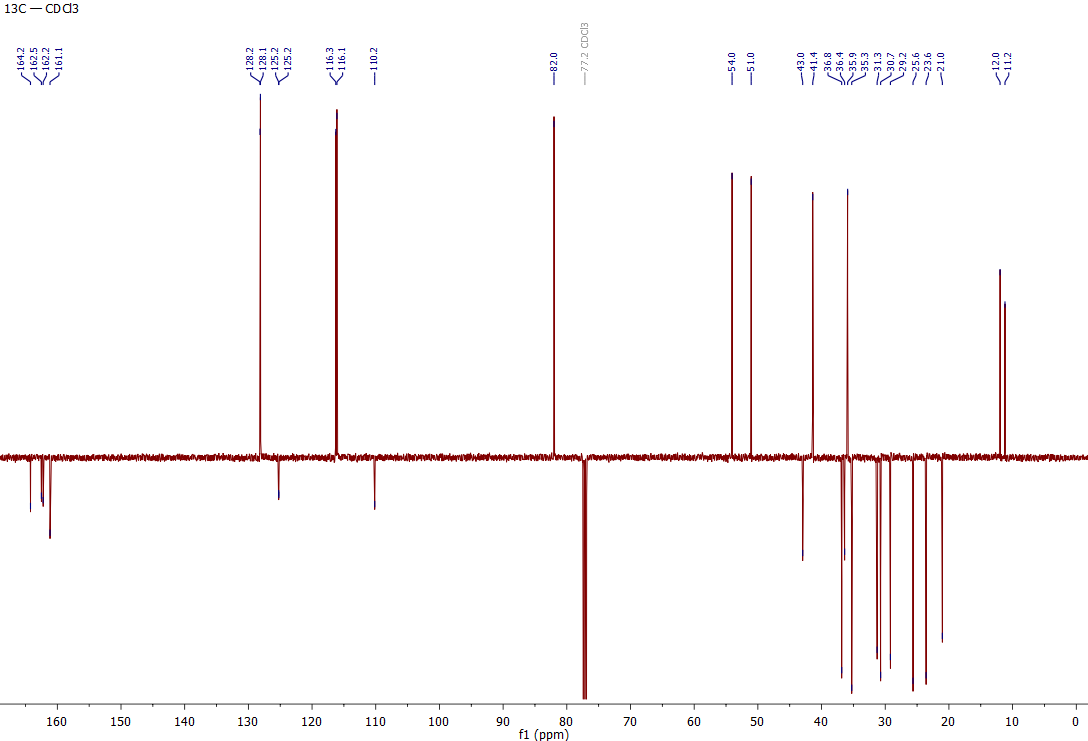


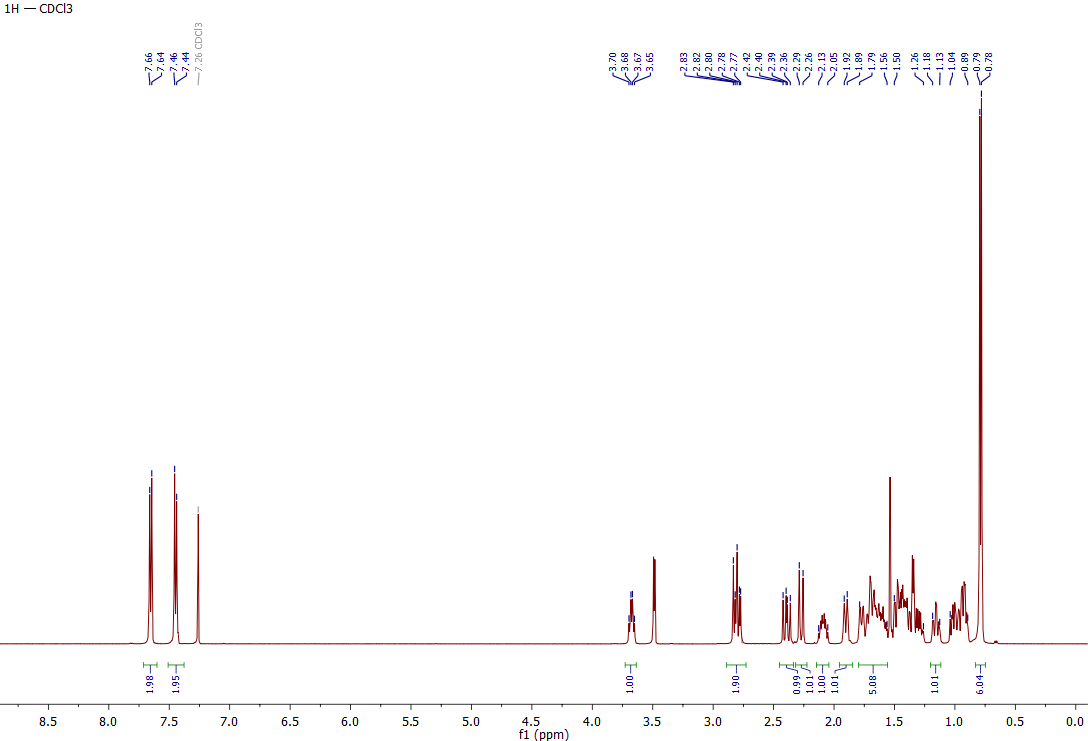

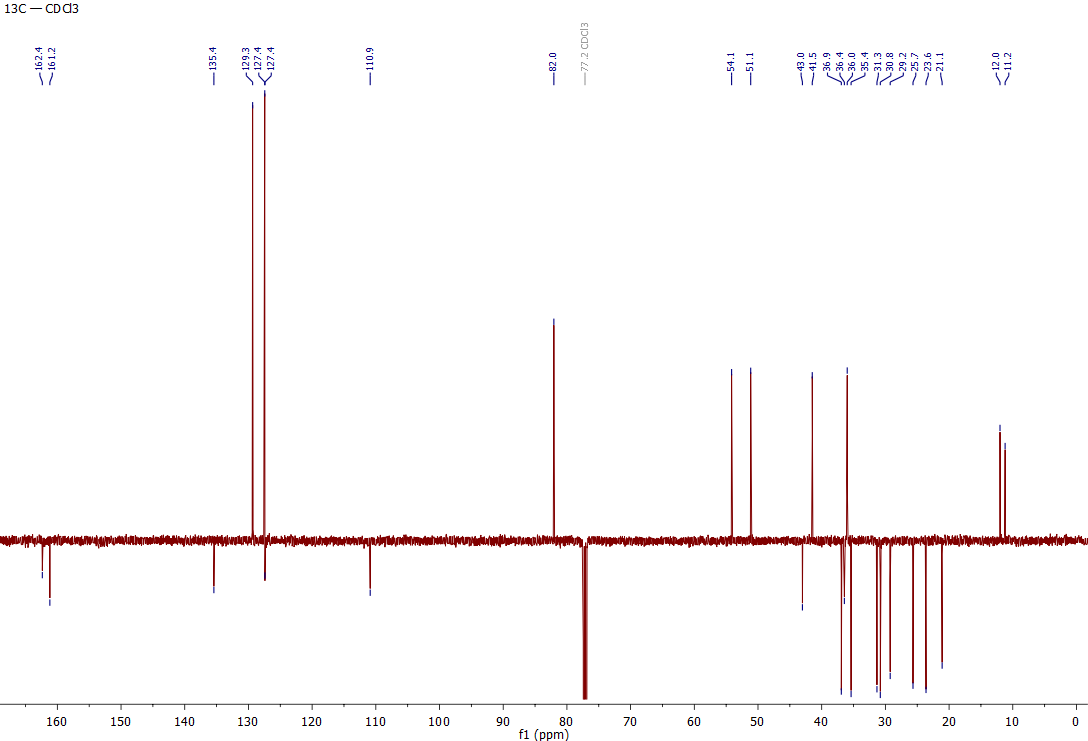


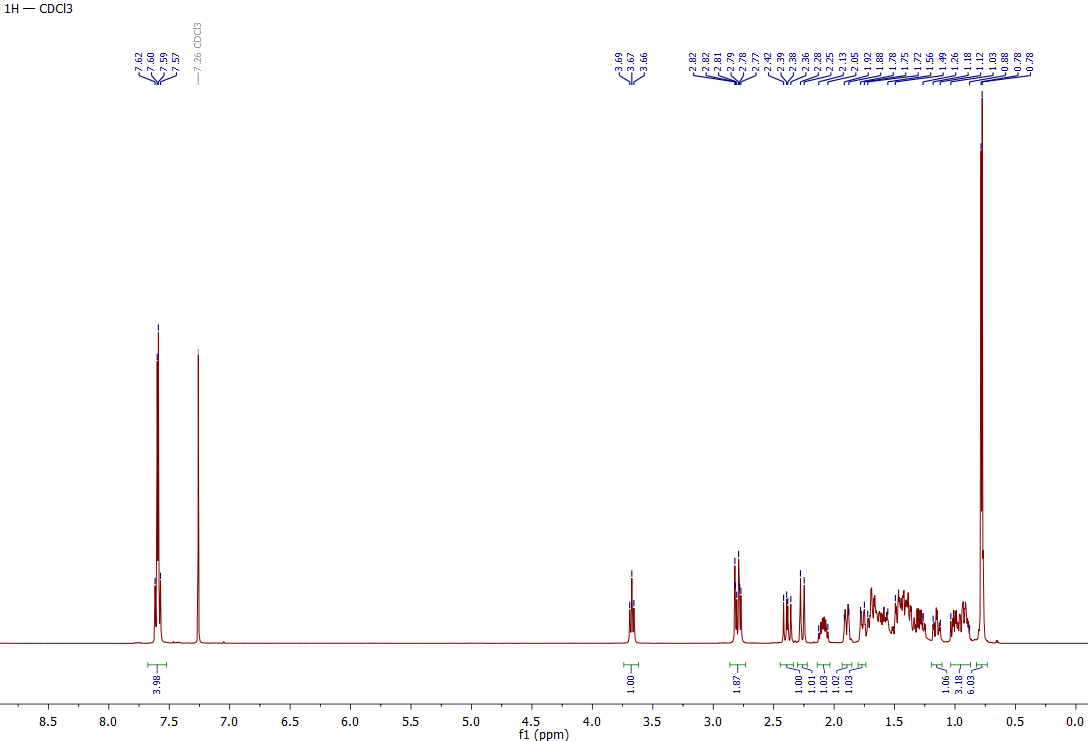

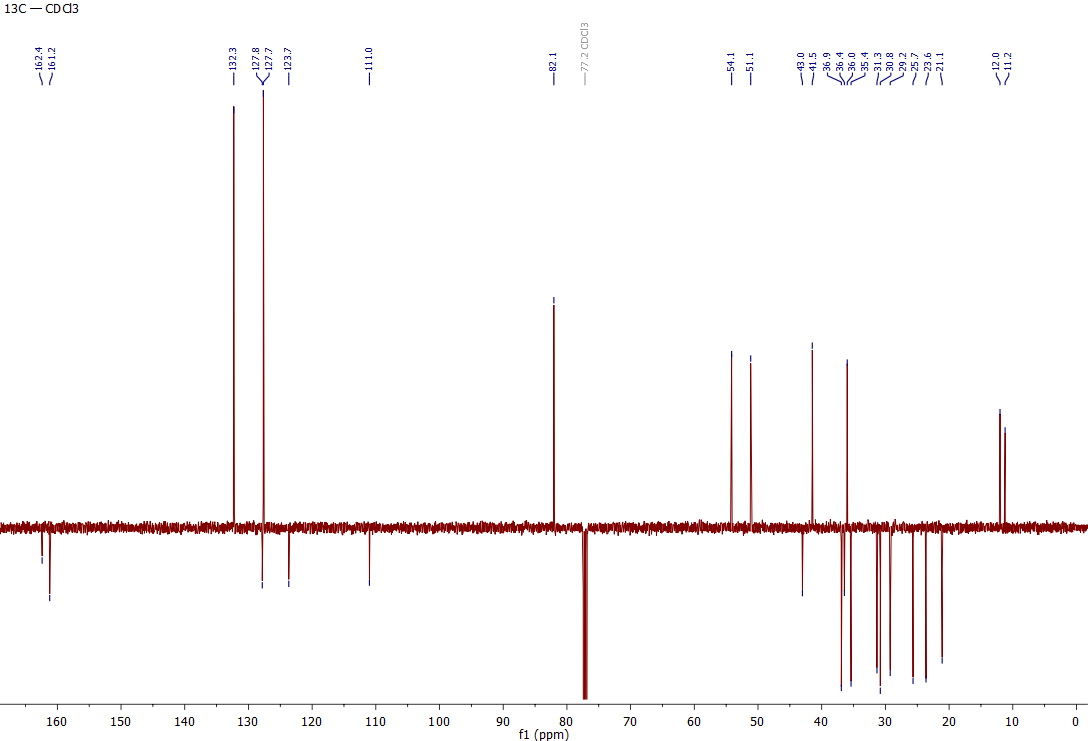


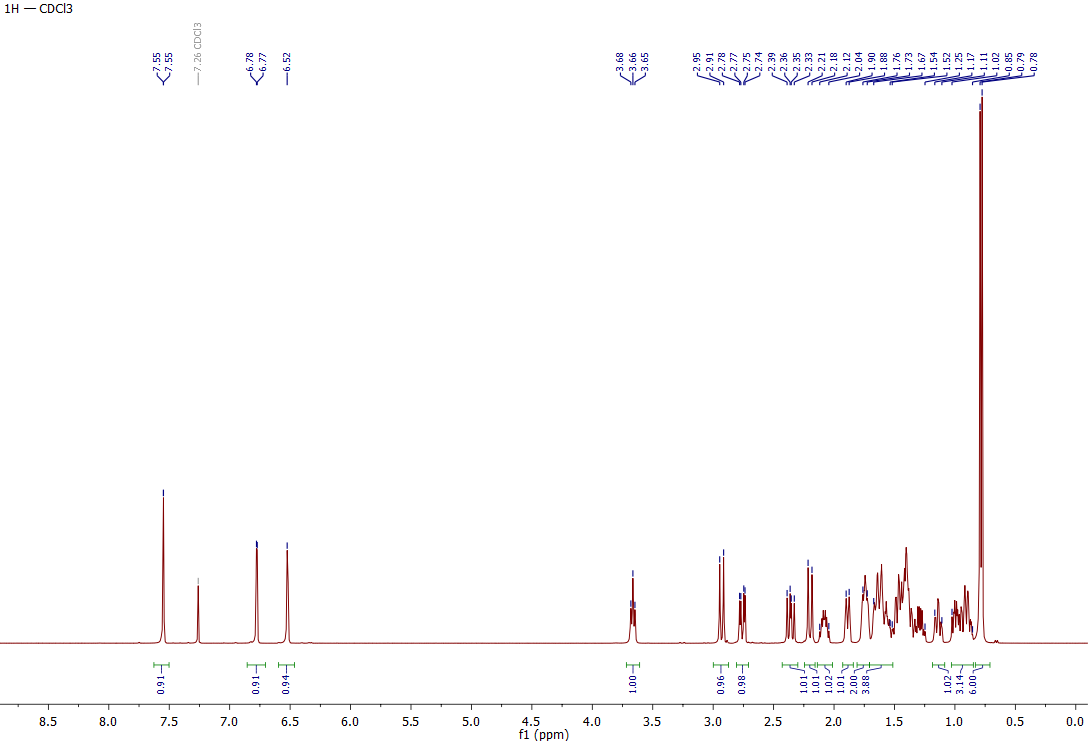

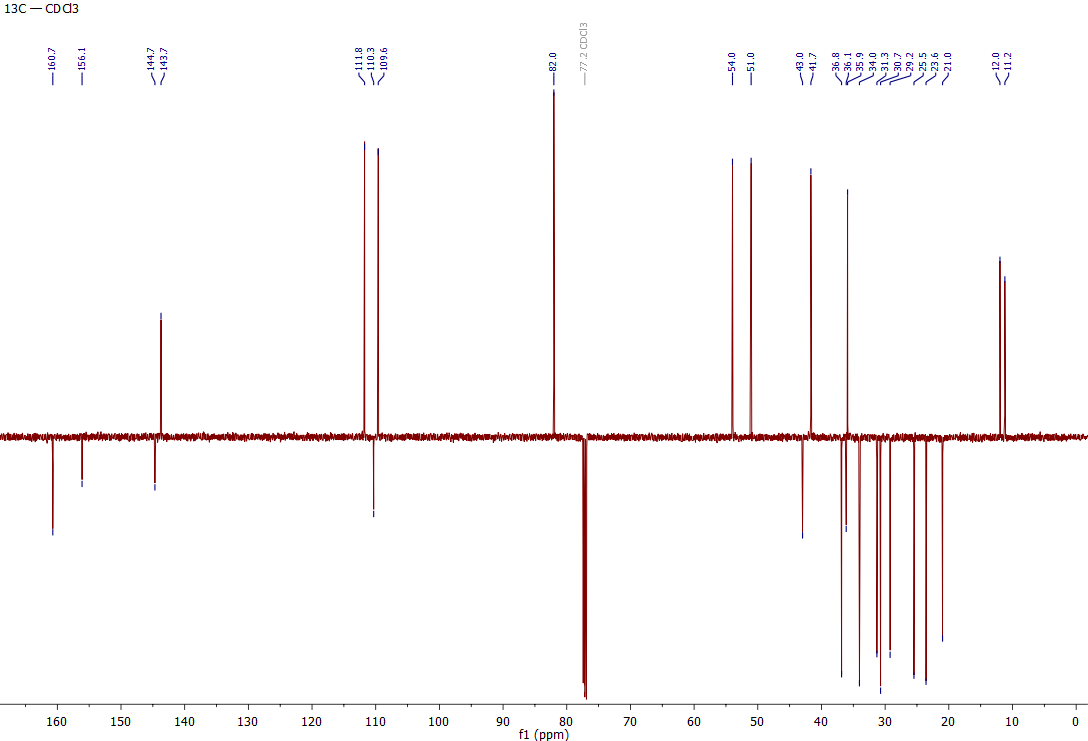


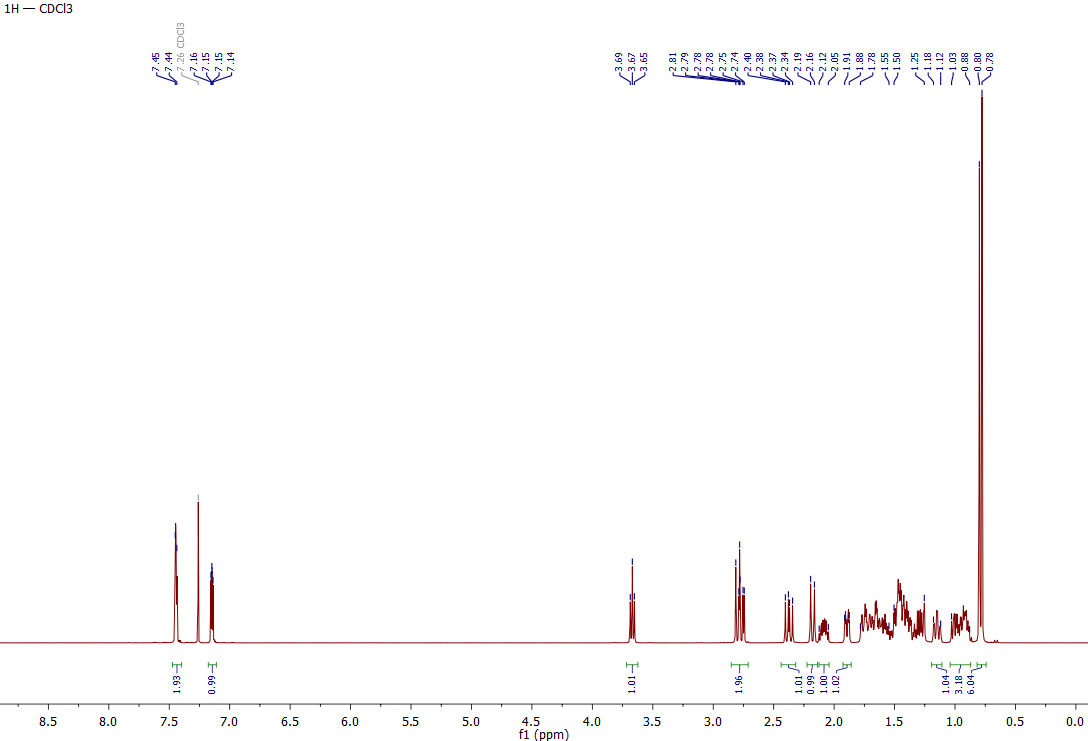

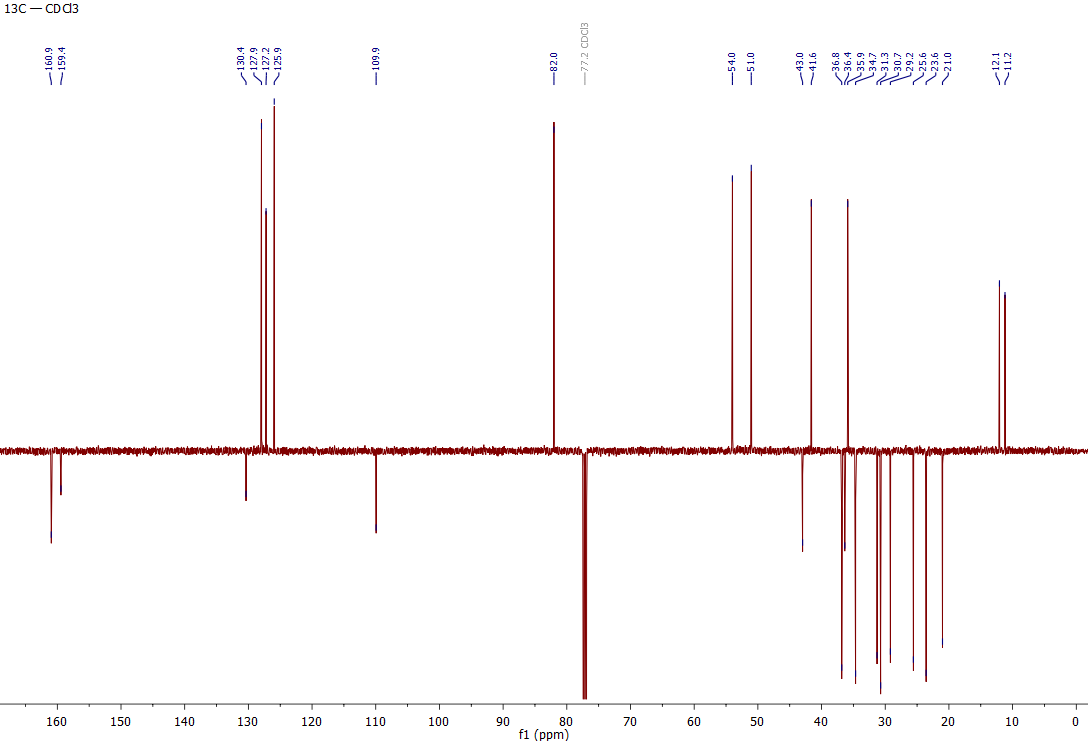


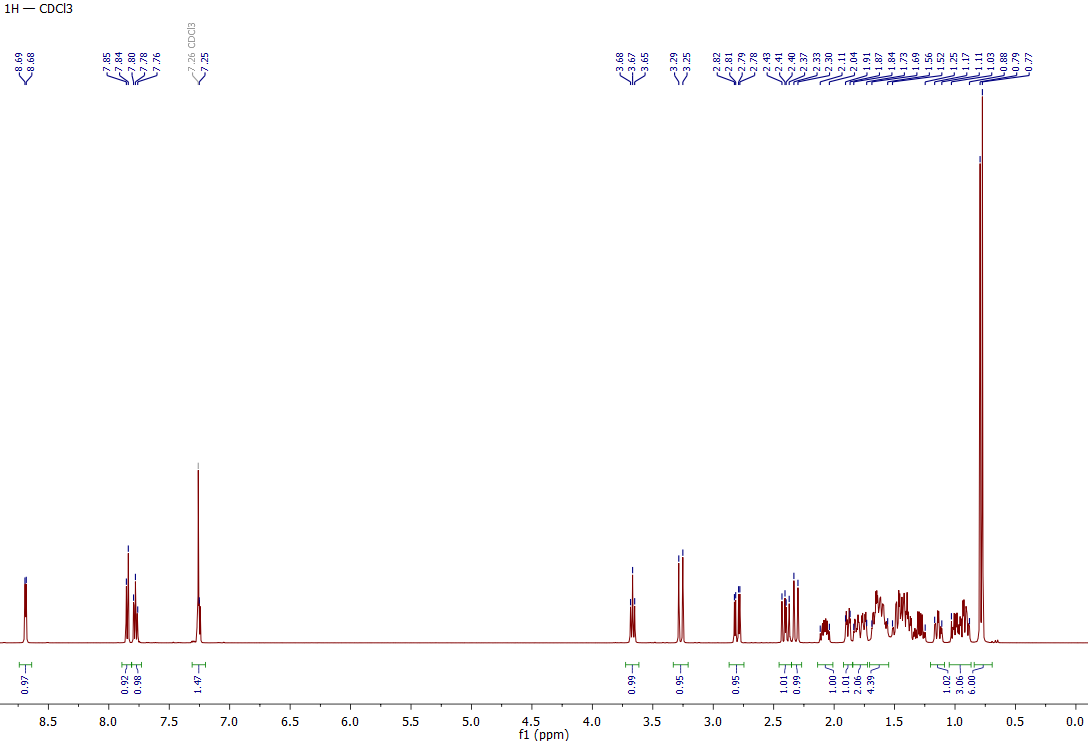

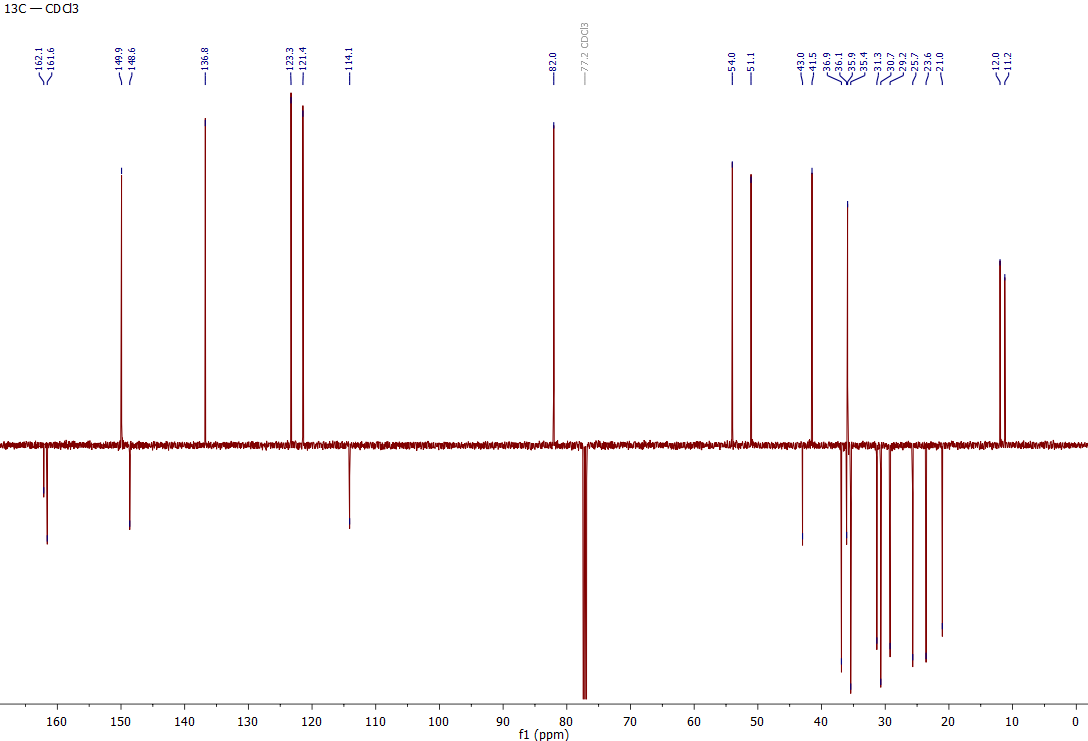


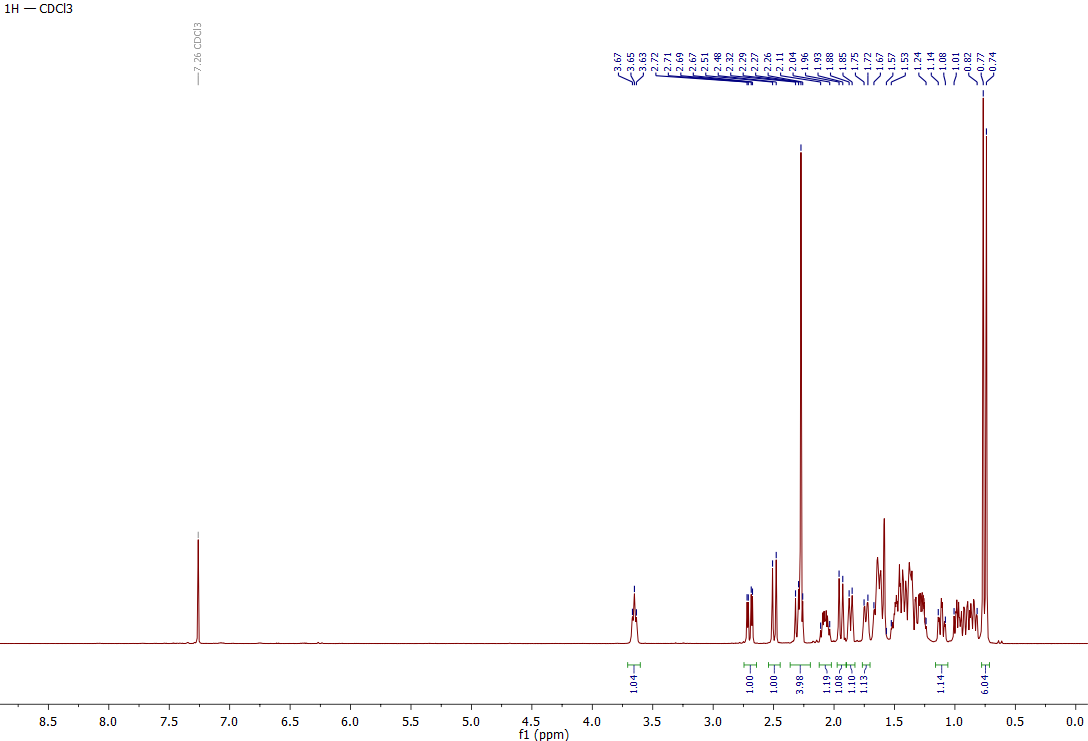

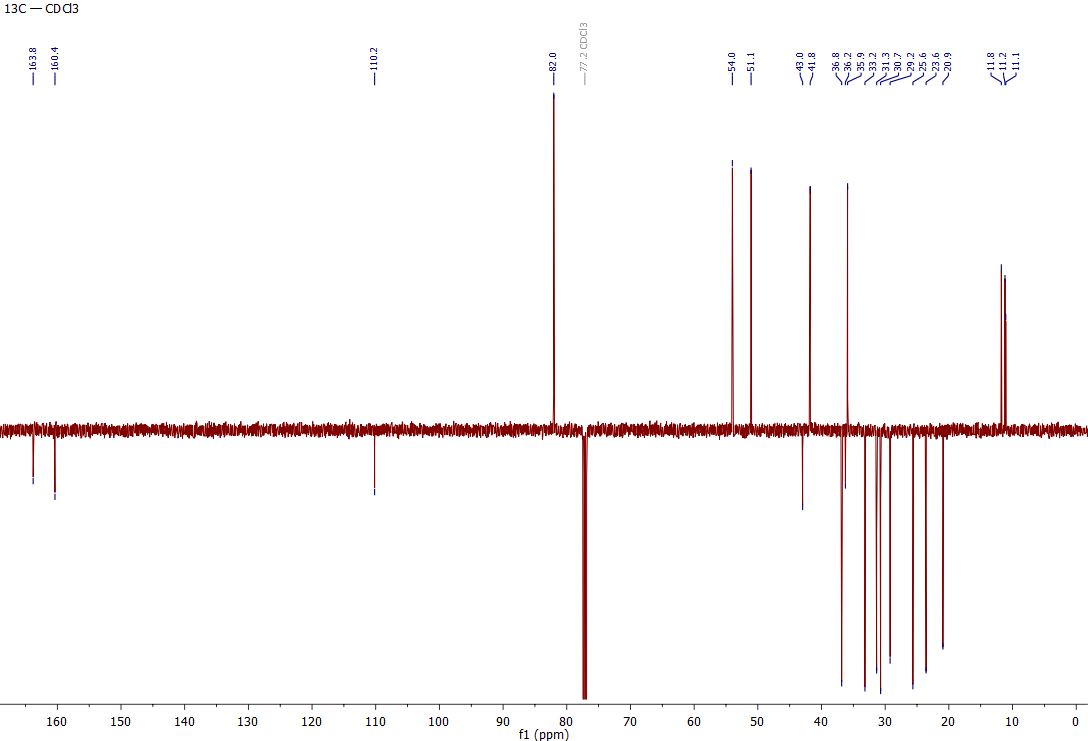


**Supplementary Table 3.** UHPLC purity and HRMS results.

| compound. | UHPLC purity (AUC, %)^1^ | Exact *m/z* calculated [M+H]^+^ | HRMS *m/z* measured^2^ [M+H]^+^ |
| --- | --- | --- | --- |
| **1i** | 96.81 | 380.2584 | 380.2584 |
| **2a** | 100 | 392.2585 | 392.2584 |
| **2b** | 100 | 406.2741 | 406.2741 |
| **2c** | 100 | 422.2690 | 422.2690 |
| **2d** | 100 | 410.2490 | 410.2490 |
| **2e** | 100 | 426.2194 | 426.2194 |
| **2f** | 100 | 470.1690 | 470.1689 |
| **2g** | 97.13 | 382.2377 | 382.2377 |
| **2h** | 100 | 398.2149 | 398.2148 |
| **2i** | 98.39 | 393.2537 | 393.2537 |
| **2j** | 96.51 | 330.2428 | 330.2428 |

^1^ LC/MS analyses were carried out using UPLC-MS system consisting of UPLC chromatograph Acquity with photodiode array detector and single quadrupole mass spectrometer (Waters), using C18 X-Select HSS T3 column (see the detailed method in the Experimental section) Purity was calculated as a percent of AUC of the peak representing analysed compound from the integration of the chromatogram. Chromatograms are shown below in the **Supplementary information**.

^2^ HRMS analysis was performed using LC-MS (Dionex Ultimate 3000, Thermo Fischer Scientific, USA) with Exactive Plus Orbitrap high-resolution mass spectrometer (Thermo Exactive plus, Thermo Fischer Scientific, USA) (see the detailed method in the Experimental section) Purity was calculated as a percent of AUC of the peak representing analysed compound from the integration of the chromatogram. Chromatograms are shown below in the **Supplementary information**.

**1i**

UHPLC


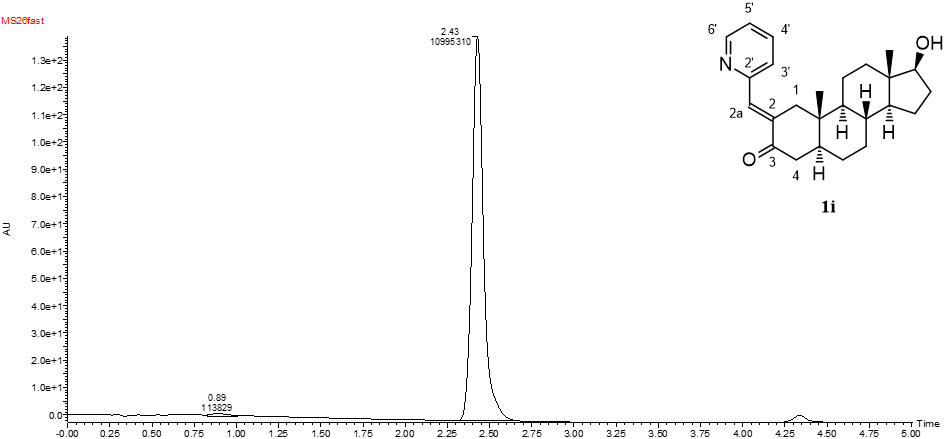


ESI+ HRMS

**2a**

UHPLC


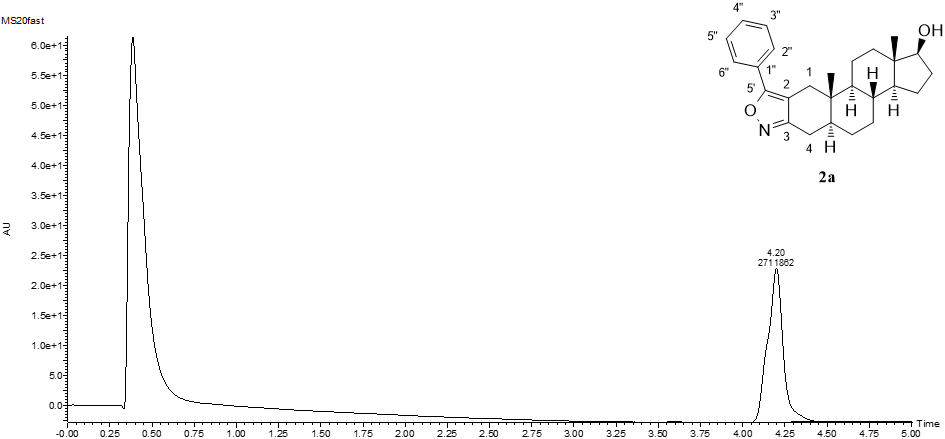


Note: Peak at 0.39 belongs to co-solvent (DMSO)

ESI+ HRMS

**2b**

UHPLC


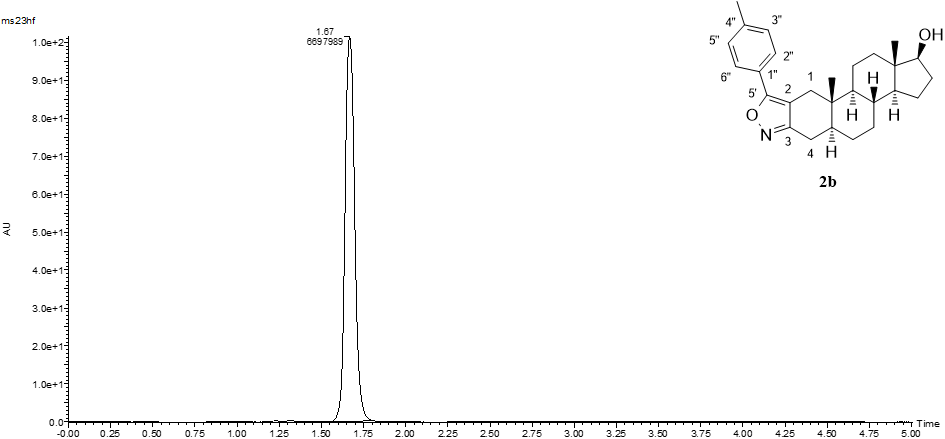


ESI+ HRMS

**2c**

UHPLC


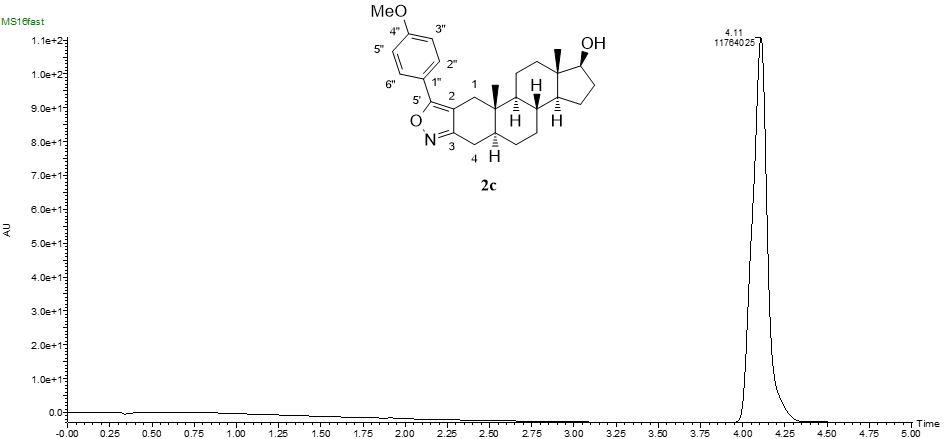


ESI+ HRMS

**2d**

UHPLC


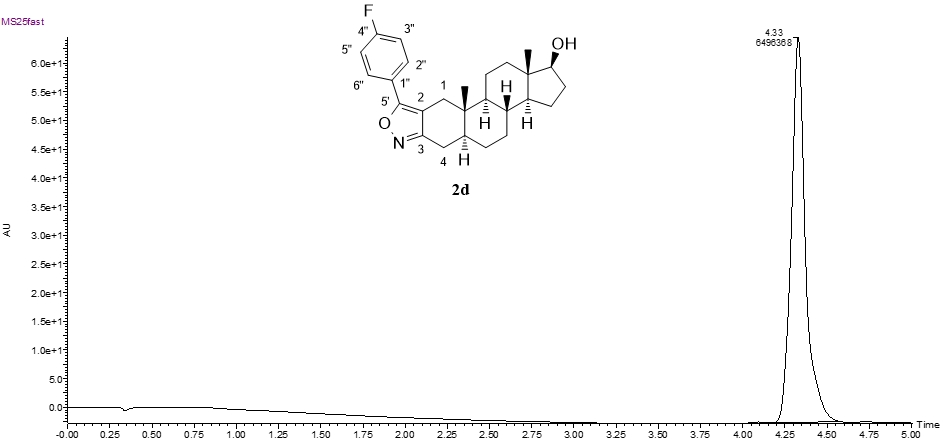


ESI+ HRMS

**2e**

UHPLC


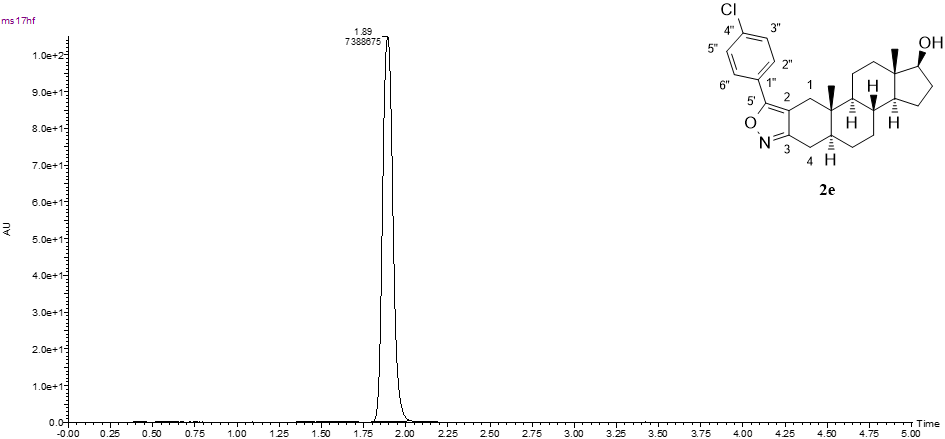


ESI+ HRMS

**2f**

UHPLC


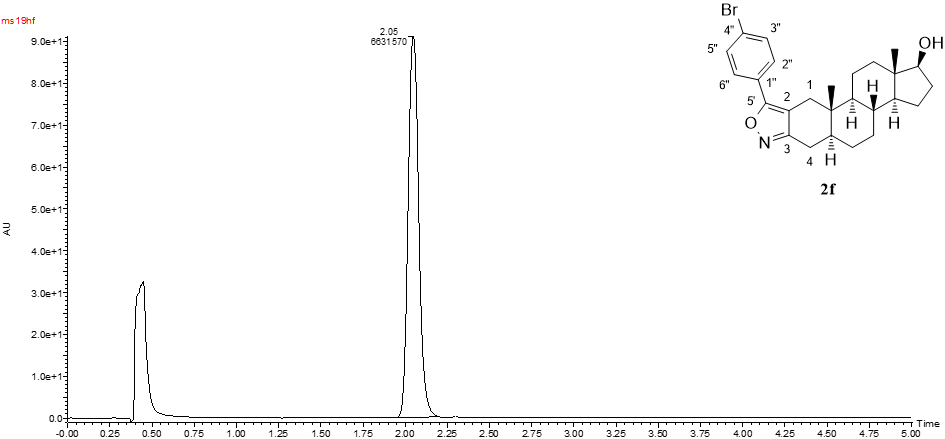


**Note: Peak at 0.49 min. belongs to co-solvent (DMSO)**

ESI+ HRMS

**2g**

UHPLC


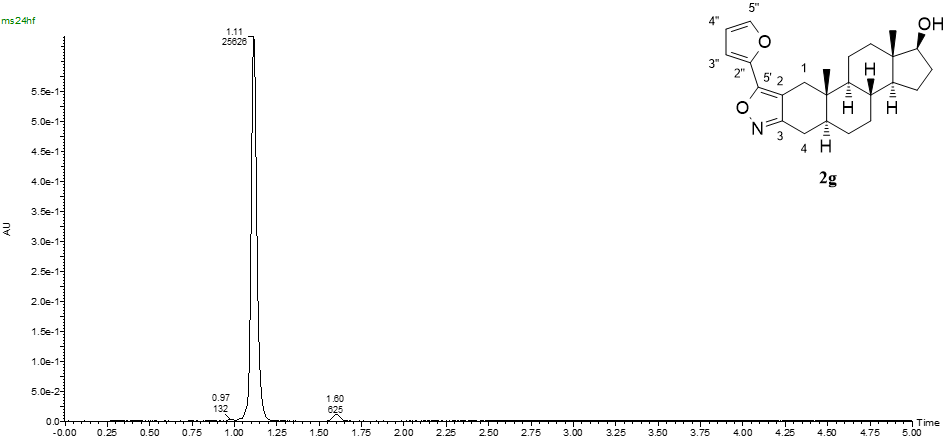


ESI+ HRMS

**2h**

UHPLC


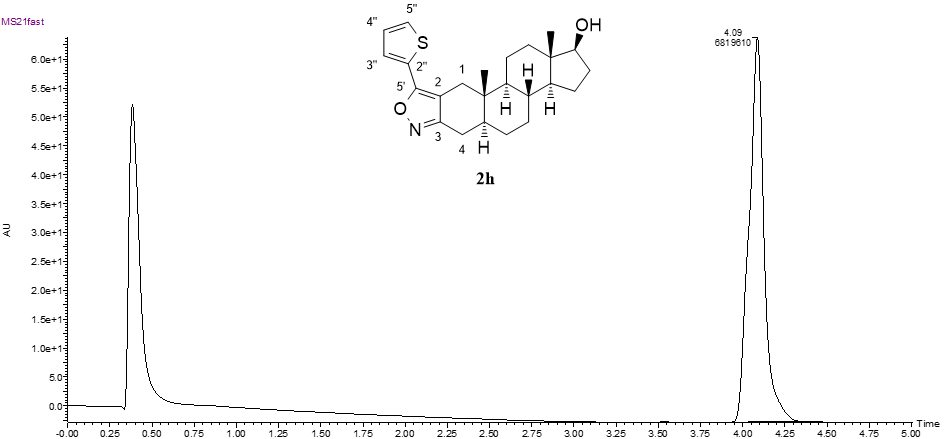


Note: Peak at 0.39 belongs to co-solvent (DMSO)

ESI+ HRMS

**2i**

UHPLC


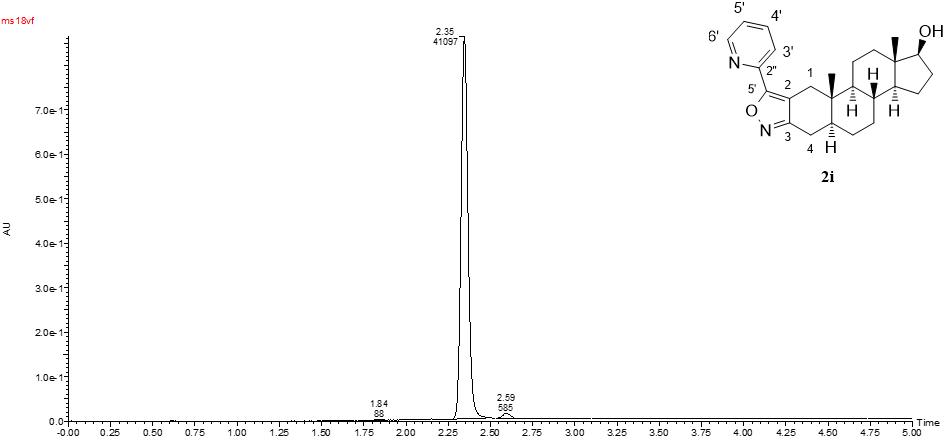


ESI+ HRMS

**2j**

UHPLC


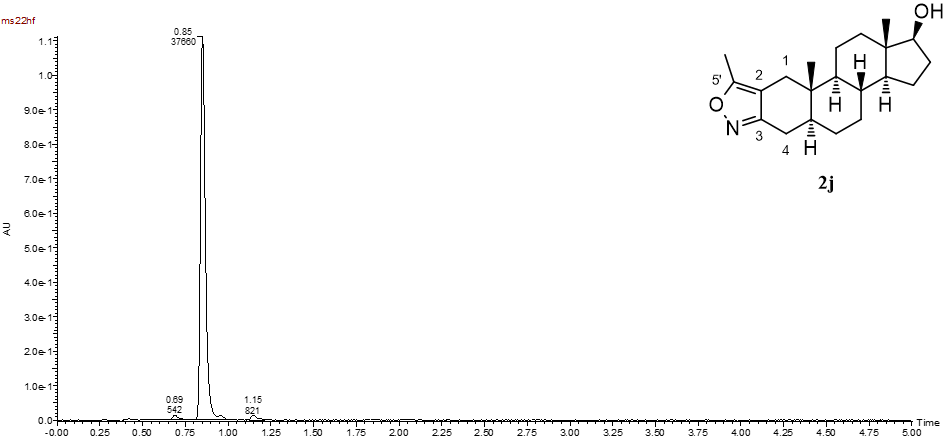


ESI+ HRMS
